# Mitochondrial membrane lipid cardiolipin controls fiber-type adaptations in aging muscle via estrogen-related receptor γ

**DOI:** 10.1038/s43587-026-01227-7

**Published:** 2026-09-29

**Authors:** Fabian Finger, Mikkel Frost, Shinya Watanabe, Tao Ma, Taewook Kang, Dipsikha Biswas, Ruben Zapata-Perez, Vincent Anton, Lucile Dollet, Lasse K. Markussen, Nanami Senoo, Iuliia Karavaeva, Astrid Linde Basse, Oksana Dmytriyeva, Tara TeSlaa, Jesper F. Havelund, Amy M. Ehrlich, Marta Moreno-Torres, Caio Yogi Yonamine, Nicoline R. Andersen, Ida Blom, Francisco Garcia-Carrizo, Piyarat Siripoksup, Magnus Asping, Thomas S. Nielsen, Marcus Nygård, Anders B. Klein, Andrey Tvardovskiy, Trine S. Nicolaisen, Carlos Durantez-Fernández, Klaus Qvortrup, Clara Prats, Joshua D. Rabinowitz, Christoffer Clemmensen, Lykke Sylow, Juleen R. Zierath, Jonas T. Treebak, Tim J. Schulz, Trisha J. Grevengoed, Steven M. Claypool, Julien Prudent, Rob C. I. Wüst, Susanne Mandrup, Riekelt H. Houtkooper, Kei Sakamoto, Nils J. Færgeman, Martin R. Larsen, Katsuhiko Funai, Steen Larsen, Zachary Gerhart-Hines

**Affiliations:** 1 Novo Nordisk Foundation Center for Basic Metabolic Research, Faculty of Health and Medical Sciences, University of Copenhagen, Copenhagen, Denmark; 2 ADIPOSIGN, Center for Adipocyte Signaling, Department of Biochemistry and Molecular Biology, University of Southern Denmark, Odense, Denmark; 3 Diabetes and Metabolism Research Center, University of Utah, Salt Lake City, UT USA; 4 Department of Biochemistry and Molecular Biology, VILLUM Center for Bioanalytical Sciences, University of Southern Denmark, Odense, Denmark; 5 Laboratory Genetic Metabolic Diseases, Amsterdam Gastroenterology, Endocrinology and Metabolism, Amsterdam UMC, University of Amsterdam, Amsterdam, Netherlands; 6 Group of Metabolism and Gene Regulation, UCAM HiTech Sport & Health Innovation Hub, Universidad Católica de Murcia, Murcia, Spain; 7 Medical Research Council Mitochondrial Biology Unit, University of Cambridge, Cambridge, UK; 8 Department of Molecular Medicine and Surgery, Karolinska Institute, Stockholm, Sweden; 9 Functional Genomics & Metabolism Research Unit, Department of Biochemistry and Molecular Biology, University of Southern Denmark, Odense, Denmark; 10 Department of Physiology, Pharmacology and Therapeutics, The Johns Hopkins University School of Medicine, Baltimore, MD USA; 11 Lewis Sigler Institute for Integrative Genomics, Princeton University, Princeton, NJ USA; 12 Department of Molecular and Medical Pharmacology, David Geffen School of Medicine, University of California, Los Angeles, Los Angeles, CA USA; 13 Department of Biochemistry and Molecular Biology, University of Valencia, Valencia, Spain; 14 Experimental Hepatology and Liver Transplant Unit, Health Research Institute La Fe, Valencia, Spain; 15 CIBER of Hepatic and Digestive Diseases (CIBERehd), Instituto de Salud Carlos III, Madrid, Spain; 16 Department of Biomedical Sciences, University of Copenhagen, Copenhagen, Denmark; 17 Department of Adipocyte Development and Nutrition, German Institute of Human Nutrition Potsdam-Rehbrücke, Nuthetal, Germany; 18 German Center for Diabetes Research (DZD), Neuherberg, Germany; 19 Institute of Functional Epigenetics, Helmholtz Zentrum München, Neuherberg, Germany; 20 Core Facility for Integrated Microscopy, Department of Biomedical Sciences, University of Copenhagen, Copenhagen, Denmark; 21 Department of Chemistry, Princeton University, Princeton, NJ USA; 22 Ludwig Institute for Cancer Research, Princeton Branch, Princeton, NJ USA; 23 Institute of Nutritional Science, University of Potsdam, Nuthetal, Germany; 24 Department of Human Movement Sciences, Faculty of Behavioural and Movement Sciences, Amsterdam Movement Sciences, Vrije Universiteit Amsterdam, Amsterdam, Netherlands; 25 Clinical Research Centre, Medical University of Bialystok, Bialystok, Poland; 26 Institute of Sports Medicine Copenhagen, Department of Orthopedic Surgery M, Copenhagen University Hospital–Bispebjerg and Frederiksberg, Copenhagen, Denmark

**Keywords:** Ageing, Mitochondria

## Abstract

Aging paradoxically leads to both a decline in skeletal muscle mitochondrial function and a shift in muscle composition that favors fibers rich in mitochondria. Yet the biological rationale and mechanism underlying this phenomenon remain largely unknown. Here we show that synthesis of the mitochondrial membrane lipid, cardiolipin, causally links mitochondrial dysfunction to fiber-type adaptations in aging mouse and human skeletal muscle. By mimicking the aging decline of skeletal muscle cardiolipin levels in young mice using inducible tissue-specific cardiolipin synthase 1 (*Crls1*) deletion, we could reproduce key aging hallmarks, including the shift from glycolytic to oxidative fibers. This shift is mediated by mitochondria-to-nucleus signaling through the nuclear receptor, estrogen-related receptor γ, which promotes reactive oxygen species-sensitive glucose uptake and enhanced glycolytic rerouting to sustain antioxidant defenses. Restoring *Crls1* expression in adult *Crls1* knockout mice reestablishes cardiolipin levels, initiates reversal of muscle atrophy and fully rescues premature mortality. These findings reveal how changes in a mitochondrial membrane lipid cell autonomously orchestrate fiber-type adaptations in aging and myopathies.

## Abstract (web summary)

Finger et al. report that declining synthesis of the mitochondrial membrane lipid cardiolipin is a feature of aging in mouse and human skeletal muscle, driving age-related fiber-type and metabolic adaptations through mitochondria-to-nucleus communication.

## Main

Skeletal muscle is composed of fiber types that span a continuum of contractile and metabolic properties, enabling the tissue to meet diverse functional demands. Along this spectrum, slow-twitch (type I) fibers show high mitochondrial oxidative capacity, whereas fast-twitch fibers comprise both oxidative (type IIa) and glycolytic (type IIx and IIb) subtypes that rely differently on oxidative versus glycolytic metabolism for force generation^1,2^. Diminished mitochondrial function disrupts systemic energy homeostasis and is a hallmark of myopathies^3^ and aging^4,5^. Yet, seemingly paradoxically, myopathies and aging also lead to selective atrophy of glycolytic fibers and a shift toward oxidative metabolism^6,7^. The timing and mechanisms driving this fiber‑type‑specific shift remain unclear. Whether diminished mitochondrial function has a causal role in eliciting adaptive measures or is merely a marker of age- and disease-related decline is unknown.

Mitochondrial function is heavily shaped by the inner mitochondrial membrane (IMM), which coordinates cellular and organismal homeostasis by modulating macronutrient exchange, bioenergetic capacity, fusion and fission, and interactions with other organelles^8^. The IMM’s distinct curvature is conferred by a unique lipid composition, most notably the phospholipid cardiolipin (CL)^9,10^. CL is nearly confined to the IMM and central to almost every facet of mitochondrial structure and function^9,11,12^. Its diverse contributions are made possible by its four acyl chain tails, remodeled to varying saturation levels to confer distinct biochemical properties^11^. All diverse CL species originate from the rate-limiting synthesis reaction of a single CL synthase, cardiolipin synthase 1 (CRLS1).

The disruption of CL is associated with a myriad of diseases^10,13–16^, including cardiomyopathy, neurodegeneration and Barth syndrome^17^. Previous analysis of a single muscle type in mice observed CL deficiency in aged animals^18^. Yet whether such changes are consequences of or actively participate in aging and myopathic development remains unclear, as do the age-related fiber-type switching more broadly. We therefore investigated whether CL loss is a conserved signature of aging across fiber types and species and how this critical mitochondrial membrane lipid causally contributes to the fundamental adaptations and pathological consequences of aging and disease.

## Results

### Loss of CL synthesis drives fiber-type-specific hallmarks of skeletal muscle aging

**Fig. 1:**
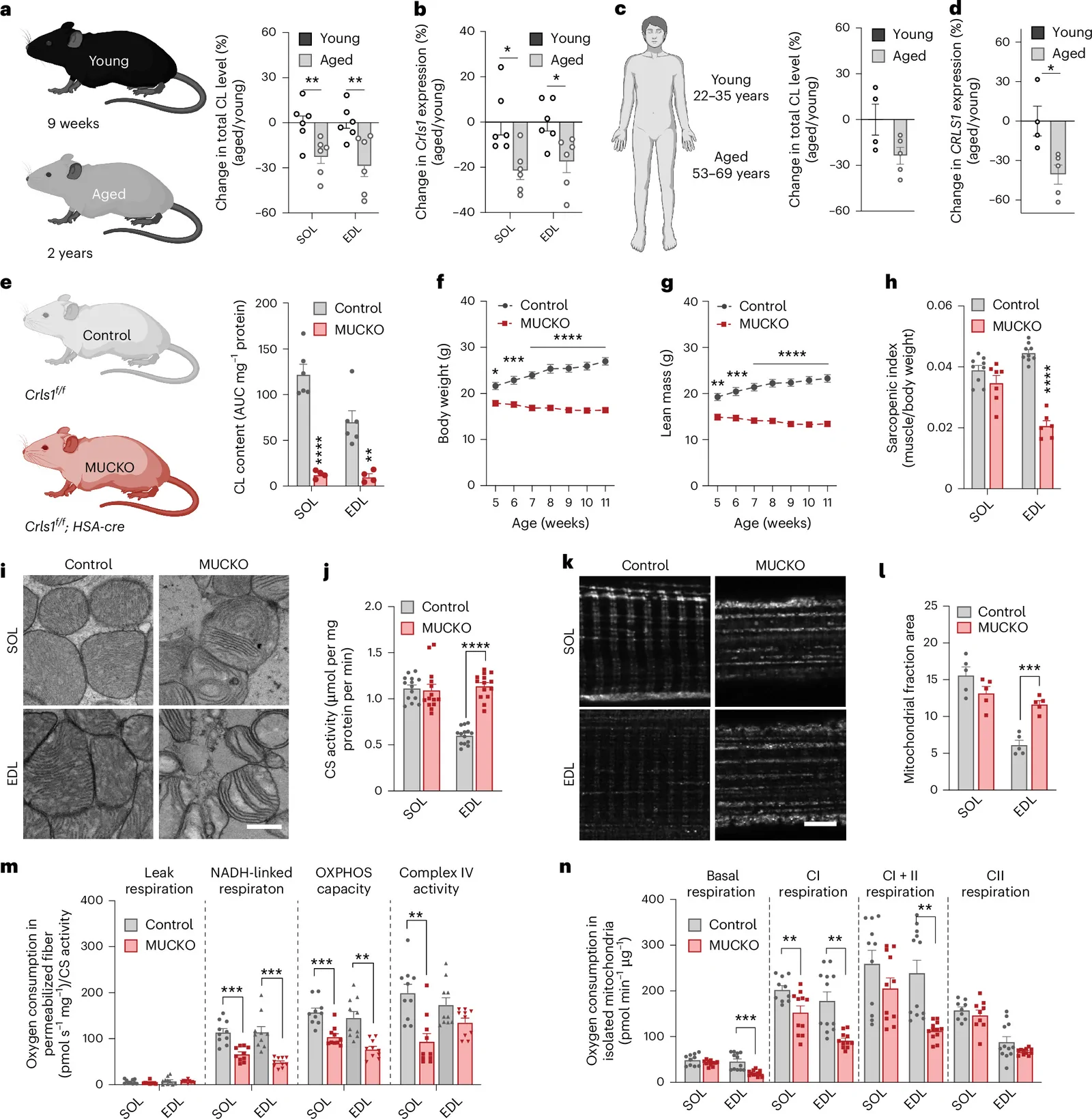
Loss of CL synthesis drives fiber-type-specific hallmarks of skeletal muscle aging. **a**, Schematic depicting young (9 weeks of age) and aged (2 years of age) mice and percentage changes of SOL and EDL total CL levels in aged compared with young mice (*n* = 6). **b**, Percentage changes of *Crls1* expression in SOL and EDL in aged compared with young mice (*n* = 6). **c**, Schematic of human cohort consisting of young (23–35 years of age) and aged (53–69 years of age) individuals and percentage changes of SOL and EDL total CL levels in aged (*n* = 5) compared with young (*n* = 4) human individuals. **d**, Percentage changes in *CRLS1* expression in aged (*n* = 5) compared with young (*n* = 4) human individuals. **e**, Schematic depicting mouse lines for constitutive skeletal muscle-specific *Crls1* knockout (MUCKO) and changes in SOL and EDL total CL levels (*n* = 6 control, 4 MUCKO). **f**,**g**,**h**, Body weight trajectory (*n* = 10) (**f**), lean mass (*n* = 10) (**g**) and sarcopenic index of SOL and EDL muscle from MUCKO mice and control littermates (*n* = 9 control, 7 MUCKO) (**h**). **i**,**j**,**k**,**l**, TEM (scale bar, 0.5 µm; representative images from 3 individuals per group) (**i**), CS activity (*n* = 14) (**j**), representative COX IV staining (scale bar, 5 µm; representative images related to **l**) (**k**) and quantification of COX IV-positive mitochondria (*n* = 5) (**l**) in SOL and EDL from MUCKO mice and control littermates. **m**,**n**, CS-corrected mitochondrial respiration of permeabilized muscle (*n* = 10) (**m**) and isolated mitochondria (*n* = 12) (**n**) of SOL and EDL MUCKO mice and control littermates. Data are presented as mean ± s.e.m. *P < 0.05; **P < 0.01; ***P < 0.001; ****P < 0.0001. Illustrations in **a**, **c** and **e** created in BioRender; Finger, F. https://biorender.com/m3x8qqk (2026). Source data

**Extended Data Fig. 1:**
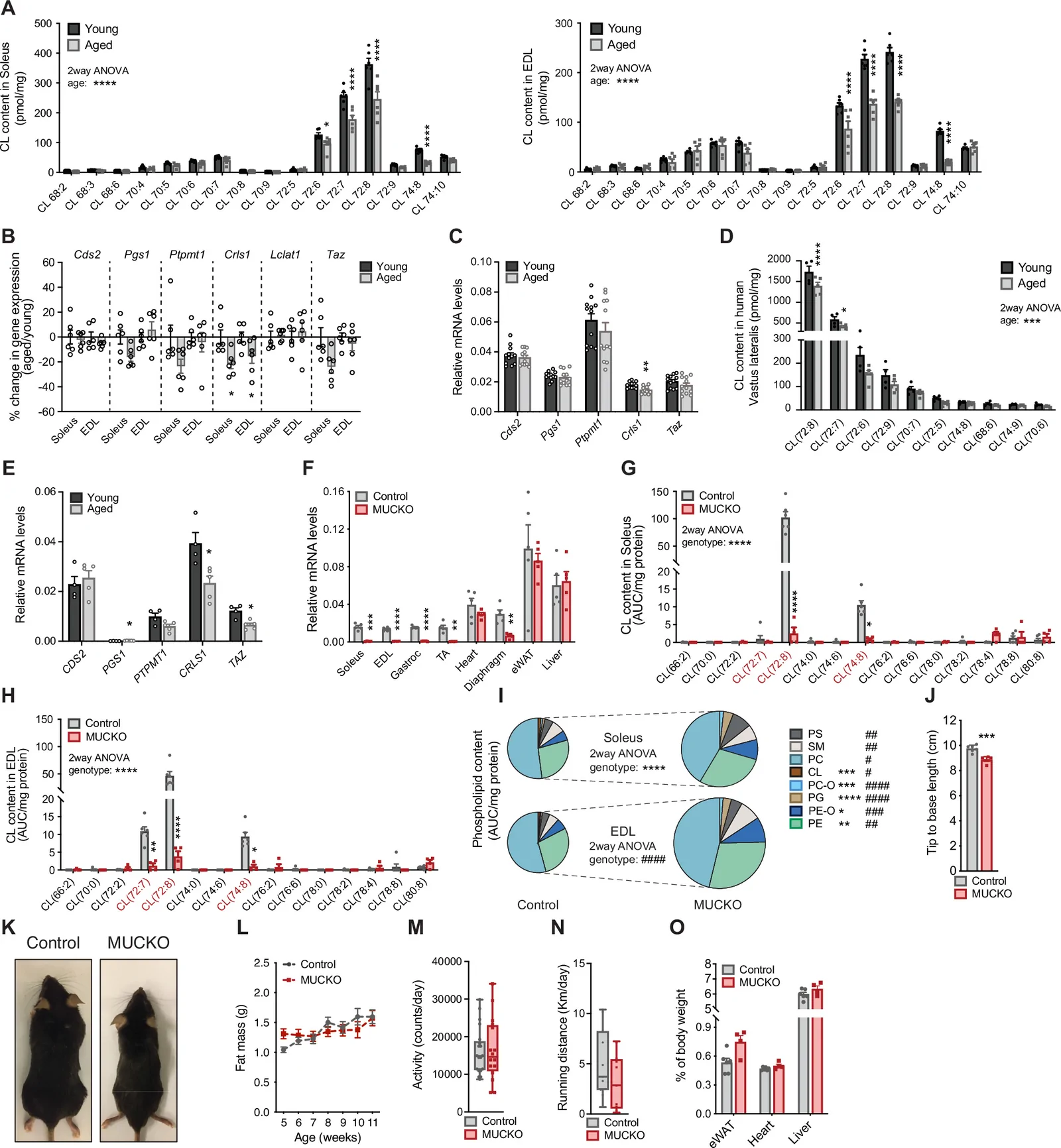
Cardiolipin remodeling in aged muscle and MUCKO mice. (**A**) Cardiolipin species in Soleus and EDL from young and aged mice (*n* = 6). (**B-C**) Percent changes (B) and relative mRNA level (C) of CL biosynthesis factors in Soleus and EDL in aged compared to young mice (*n* = 6 for B; *n* = 12 for C). (**D-E**) Top 10 most abundant CL species (D) and relative mRNA level (E) of CL biosynthesis factors in young vs aged human Vastus lateralis (*n* = 4 young, 5 aged). (**F**) *Crls1* gene expression across tissues in MUCKO mice compared to control littermates (*n* = 5). (**G-H**) Soleus (G) and EDL (H) cardiolipin species in MUCKO mice compared to control littermates (*n* = 6 control, 4 MUCKO). Red font: significantly changed in aged mice. (**I**) Changes in combined phospholipid composition in MUCKO mice (*n* = 6) compared to control littermates (*n* = 4). (**J**) body length (*n* = 6), (**K**) representative images, (**L**) fat mass (*n* = 10), (**M**) locomotion (*n* = 21 control, 16 MUCKO), (**N**) free wheel running (*n* = 11 control, 9 MUCKO), and (**O**) tissue weights (*n* = 6 control, 4 MUCKO) from MUCKO mice and control littermates. Data are presented as mean ± SEM, *p < 0.05; **p < 0.01; ***p < 0.001; ****p < 0.0001. Source Data

We first assessed aging in both oxidative soleus (SOL) and glycolytic extensor digitorum longus (EDL) muscles and found that CL levels declined similarly with age across muscle types (Fig. 1a and Extended Data Fig. 1a). We then compared the expression of CL synthesis and remodeling enzymes. Despite trends for *Pgs1* and *Taz* to be decreased (Extended Data Fig. 1b), the gene most significantly reduced in SOL and EDL encoded the CL synthase, *Crls1* (Fig. 1b and Extended Data Fig. 1c). We found a similar pattern in aged human muscle, with total CL levels declining in aged (53–69 years) versus young (22–35 years) participants, alongside significant age-dependent alterations in individual CL species composition (Fig. 1c and Extended Data Fig. 1d). Transcriptional analyses similarly revealed a pronounced decrease in genes linked to CL synthesis (Fig. 1d and Extended Data Fig. 1e), pointing to a conserved age-associated regulation of CL biology.

We selectively ablated *Crls1* in the skeletal muscle of mice (hereby referred to as MUCKO) to interrogate its contribution to age- and disease-associated outcomes. In MUCKO mice, *Crls1* mRNA was significantly decreased in oxidative and glycolytic skeletal muscles, but not in the heart, epididymal white adipose tissue (eWAT) or liver (Supplementary Fig. 1f). The reduction in *Crls1* expression led to a global reduction in CL species comparable across skeletal muscle types (Fig. 1e), with the observed decrease in the most abundant CL species (Extended Data Fig. 1g,h) mirroring the pattern observed in aged mice (Extended Data Fig. 1a). Despite representing less than 2% of the phospholipidome, the loss of CLs profoundly altered the skeletal muscle phospholipid profile (Extended Data Fig. 1I). MUCKO mice also failed to match control littermates in weight gain shortly after weaning (Fig. 1f), reflected also by smaller body length (Extended Data Fig. 1j,k) and due entirely to a failure to gain lean mass (Fig. 1g). Levels of adiposity were indistinguishable from controls (Extended Data Fig. 1l). Mice lacking skeletal muscle CL retained similar physical activity levels as controls in both free cage movement and voluntary wheel running (Extended Data Fig. 1m,n). While the blunted weight gain was attributable to loss of skeletal muscle, this effect was not uniformly distributed: despite oxidative muscle being more CL-enriched than glycolytic muscle (Fig. 1e), it was EDL, not SOL, that showed more severe atrophy and a lower sarcopenic index in MUCKOs (Fig. 1h and Extended Data Fig. 1o).

**Extended Data Fig. 2:**
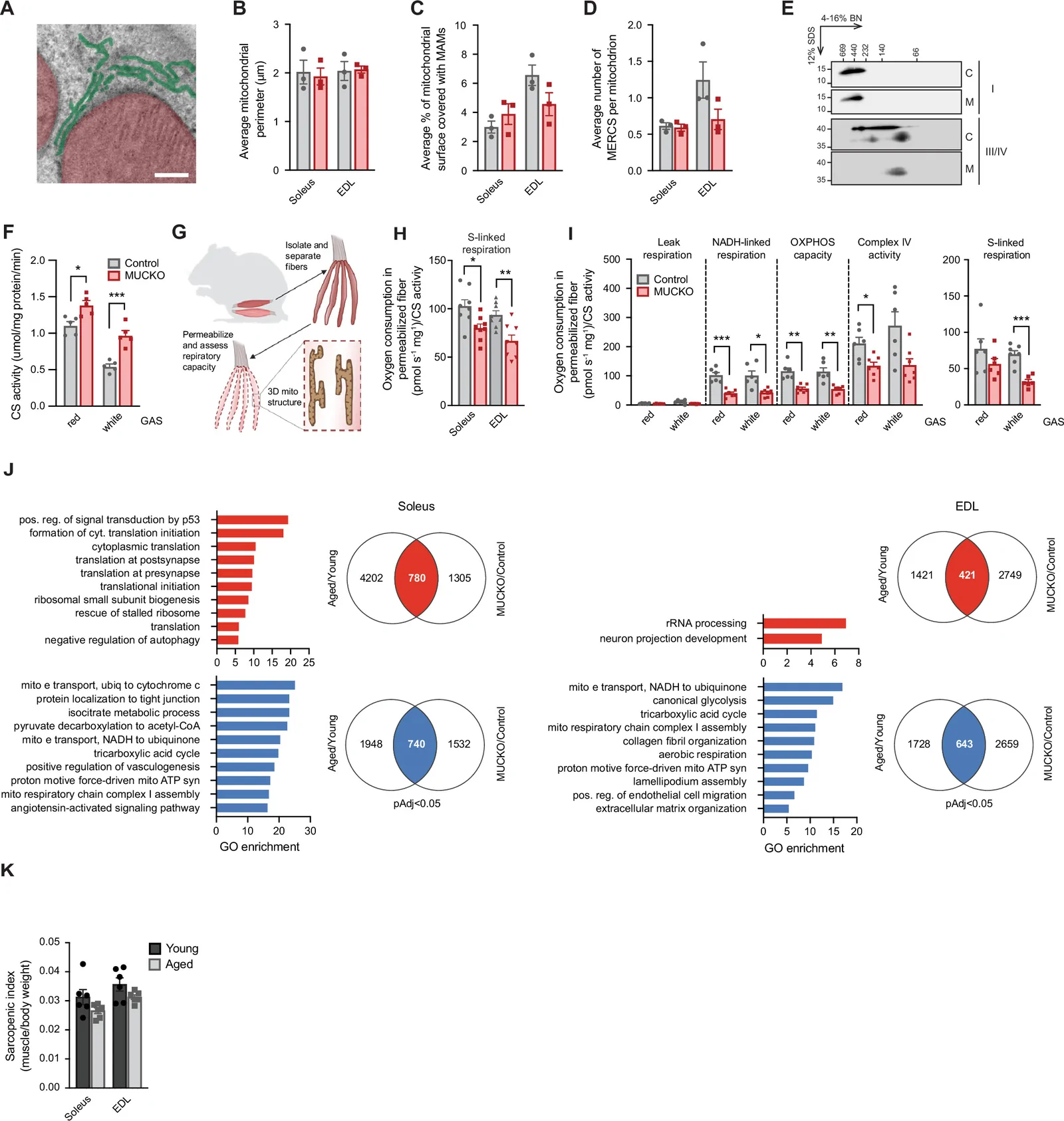
Mitochondrial contacts and respiratory function upon CL depletion. (**A-D**) Representative transmission electron microscopy image of mitochondria-ER contact sites (MERCS) with mitochondria colored in red and the ER in green. Scale bar: 200 nm. Morphometric analysis of mitochondria from Soleus and EDL muscles comparing MUCKO and control, showing (B) mitochondrial perimeter, (C) number of MERCS per mitochondrion and, (D) percentage of mitochondrial perimeter closely associated with ER membrane ( < 50 nm). Quantification was performed in EDL and SOL muscles from three different animals (*n* = 3). Number of mitochondria analyzed: EDL Control: 78, 27, 39 (total = 144); EDL MUCKO: 54, 66, 45 (total = 165); SOL Control: 59, 44, 36 (total = 139); SOL MUCKO: 56, 53, 36 (total = 145). (**E**) mitochondrial supercomplex formation (C; control, M; MUCKO). (**F**) Citrate synthase activity in red and white portions of GAS from MUCKO mice and control littermates (*n* = 5). (**G**) Schematic of the skeletal muscle permeabilization procedure. (**H**) S-linked respiration corrected for CS activity in Soleus and EDL from MUCKO mice and control littermates (*n* = 8). (**I**) Respirometry of red (oxidative) and white (glycolytic) portions of GAS from MUCKO mice and control littermates (*n* = 5). (**J**) Overlap of transcriptional changes in Soleus and EDL from aged/young and MUCKO/Control mice with corresponding GO term enrichment analysis (*n* = 6). Red: up-regulated. Blue: down-regulated. (**K**) sarcopenic index of Soleus and EDL from young and aged mice (*n* = 6). Data are presented as mean ± SEM, *p < 0.05; **p < 0.01; ***p < 0.001; ****p < 0.0001. Illustration in **g** created in BioRender; Finger, F. https://biorender.com/7d9pb8c (2026). Source Data

Given CL’s integral roles in mitochondrial structure and function^9,11,12^, we next evaluated its ablation’s effect on mitochondria using transmission electron microscopy (TEM). CL deficiency similarly disrupted cristae architecture in both SOL and EDL (Fig. 1I), a structural alteration commonly associated with aging^19^. We further quantified mitochondrial morphometric parameters by TEM but did not detect changes in mitochondrial perimeter or in mitochondria–endoplasmic reticulum contact sites (MERCS) in CL-deficient tissues (Extended Data Fig. 2a–d), suggesting that CL loss primarily affects cristae integrity rather than overall organelle shape or contacts. Supercomplex formation was uniformly diminished following CL depletion in isolated mitochondria from pooled hind limb muscles (Extended Data Fig. 2e), consistent with reports of age-associated supercomplex destabilization^20^. We explored whether loss of CL differentially affected mitochondrial abundance between these two metabolically distinct muscles, using the activity of the tricarboxylic acid (TCA) cycle enzyme, citrate synthase (CS), a strong correlate of skeletal muscle mitochondrial content^21^. As expected, SOL CS activity was higher than EDL CS activity in controls (Fig. 1j). While CL depletion had no impact on SOL CS activity, it increased EDL CS activity to the level of SOL from either genotype (Fig. 1j). This pattern was also observed in oxidative (red) versus glycolytic (white) portions of the gastrocnemius from controls and MUCKOs (Extended Data Fig. 2f). We further assessed mitochondrial content by immunostaining of single muscle fibers from EDL and SOL. Consistent with the changes observed in CS activity, EDL muscle lacking CL had a substantially higher mitochondrial content than EDL from littermate controls and more closely resembled the mitochondrial abundance seen in the SOL (Fig. 1k,l) and reminiscent of the glycolytic-to-oxidative fiber-type shift observed in the human muscle during aging^7^.

To determine whether these changes affected function, we assessed respiratory capacity in oxidative and glycolytic muscle of MUCKO mice and control littermates. Permeabilized muscle fibers were used to preserve native mitochondrial organization and obtain physiologically relevant measurements^22^ (Extended Data Fig. 2g). Consistent with our previous findings in brown adipocyte mitochondria^23^, the loss of CL significantly reduced respiratory capacity of both oxidative and glycolytic muscle when corrected for mitochondrial amount (CS activity): NADH-linked respiration, oxidative phosphorylation capacity, succinate-linked respiration and complex IV activity were all significantly lower in MUCKO SOL and EDL (Fig. 1m and Extended Data Fig. 2h). The same pattern was observed in isolated mitochondria from EDL and SOL or permeabilized muscle from red (oxidative) and white (glycolytic) portions of the gastrocnemius from both genotypes (Fig. 1n and Extended Data Fig. 2I). Thus, while *Crls1* ablation drove glycolytic muscle to resemble oxidative muscle in mitochondrial abundance, the loss of CL uniformly diminished respiratory capacity across fiber types, in line with the established reduction in mitochondrial activity with aging^4^. We further performed mRNA sequencing on SOL and EDL muscles from young and aged (Fig. 1a) as well as young MUCKO and control littermates and compared the significantly regulated genes. In SOL, 16% of upregulated and 28% of downregulated genes overlapped between aged and MUCKO mice, while in EDL, 23% of upregulated and 28% of downregulated genes were shared. Gene ontology (GO) enrichment analysis revealed that overlapping upregulated genes were primarily associated with alterations in protein translation, whereas downregulated genes were enriched for pathways related to mitochondrial function (Extended Data Fig. 2j). These findings indicate that mitochondrial dysfunction emerges as an early feature of muscle aging, preceding more severe manifestations such as disproportionate muscle atrophy, not yet evident in 24-month-old mice (Extended Data Fig. 2k). CL reduction alone appears sufficient to drive transcriptional and functional changes mirroring physiological aging.

### CL deficiency induces an age-associated fiber-type shift through nuclear receptor ERRγ

**Fig. 2:**
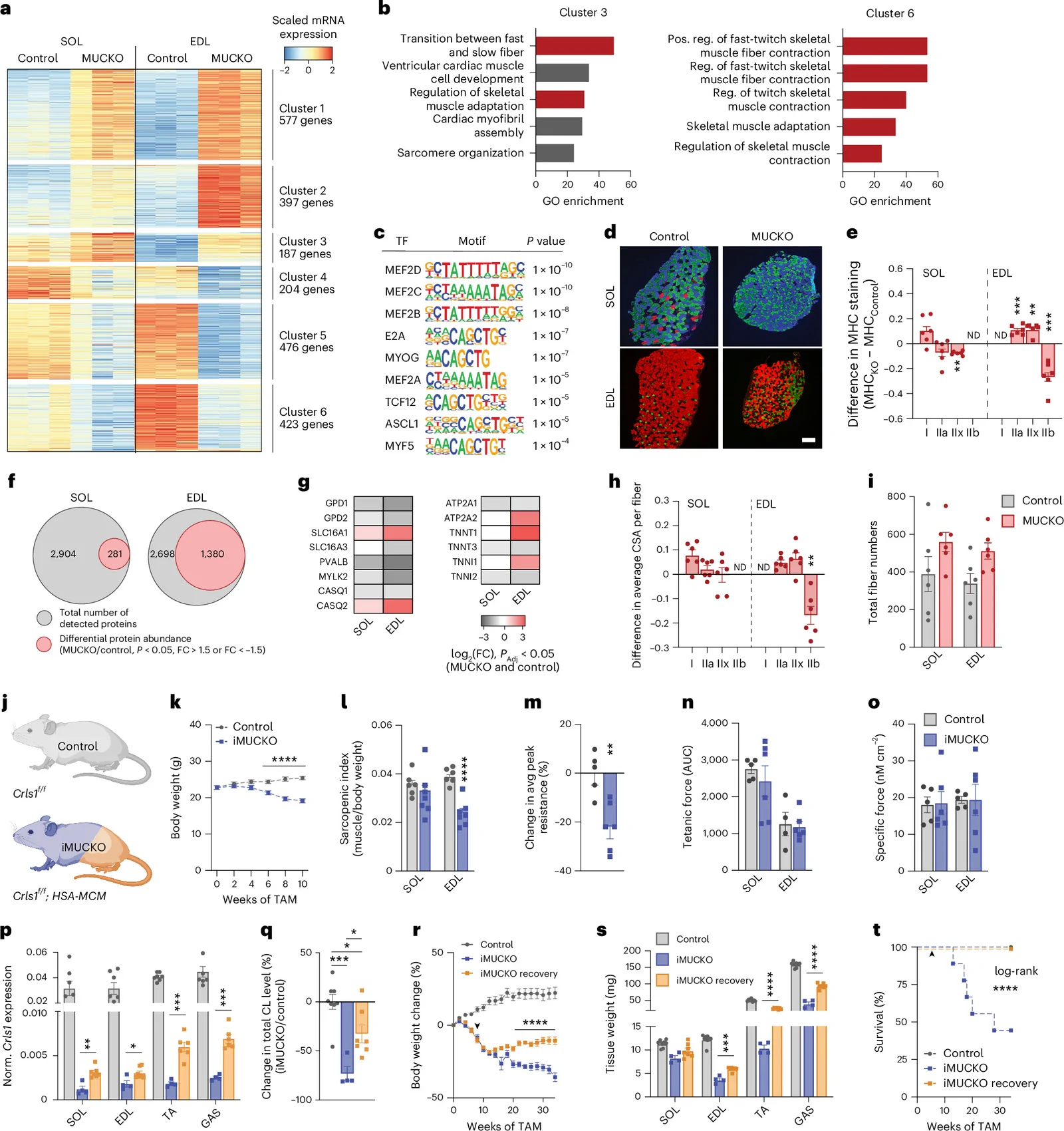
CL deficiency induces an age-associated fiber-type shift. **a**, *K*-means cluster analysis from RNA sequencing of SOL and EDL from MUCKO mice and control littermates (*n* = 3). **b**, GO term enrichment analysis of clusters 3 and 6 from the RNA sequencing. Red, skeletal muscle-specific pathways. Pos., positive; reg., regulation. **c**, Promoter motif enrichment analysis of genes in the skeletal muscle that were significantly altered by CL deletion. **d**, Immunohistochemical fiber-type-specific staining (representative images related to **e**). MHC I, blue; MHC IIa, green; MHC IIx, black; MHC IIb, red (scale bar, 100 μm). **e**, Quantification from SOL and EDL from MUCKO mice and control littermates (*n* = 6). ND, not detected. **f**,**g**, Global proteomic changes (FC > 1.5 or FC < −1.5 (MUCKO/control), *P* < 0.05) (**f**) and fiber-type-specific protein level (log_2_(FC) (MUCKO/control), *P*_Adj_ < 0.05) (**g**) of MUCKO mice and control littermates (*n* = 4 control, 5 MUCKO). **h**,**i**, Changes in average fiber-type cross-sectional area (CSA) (**h**) and fiber type counts in SOL and EDL from MUCKO mice and control littermates (*n* = 6) (**i**). **j**, Schematic depicting mouse lines for tamoxifen (TAM) inducible skeletal muscle-specific *Crls1* knockout (iMUCKO). **k**,**l**, Body weight (**k**)and sarcopenic index (**l**) of SOL and EDL muscle from iMUCKO mice and control littermates (*n* = 6 control, 7 iMUCKO). **m**,**n**,**o**, Percentage change of average (avg) peak resistance (grip strength) (**m**) and ex vivo, tetanic (**n**) and specific force production (**o**) in iMUCKO mice and control littermates (*n* = 5 control, 6 iMUCKO). **p**,**q**, *Crls1* expression in skeletal muscle (*n* = 6 control, 4 iMUCKO, 6 iMUCKO recovery) (**p**) and percentage change of total CL levels in GAS muscle from iMUCKO mice maintained on TAM and iMUCKO recovery mice in which TAM has been removed (*n* = 8 control, 4 iMUCKO, 7 iMUCKO recovery) (**q**). Norm, normalized. **r**,**s**,**t**, Body weights (*n* = 8 control, 7 iMUCKO, 8 iMUCKO recovery) (**r**), tissue weights (**s**) and survival curves in control, iMUCKO and iMUCKO recovery animals (*n* = 8 control, 4 iMUCKO, 7 iMUCKO recovery) (**t**). The arrow indicates the point at which TAM was removed. Data are presented as mean ± s.e.m. *P < 0.05; **P < 0.01; ***P < 0.001; ****P < 0.0001. Illustrations in **j** created in BioRender; Finger, F. https://biorender.com/0sym3po (2026). Source data

**Extended Data Fig. 3:**
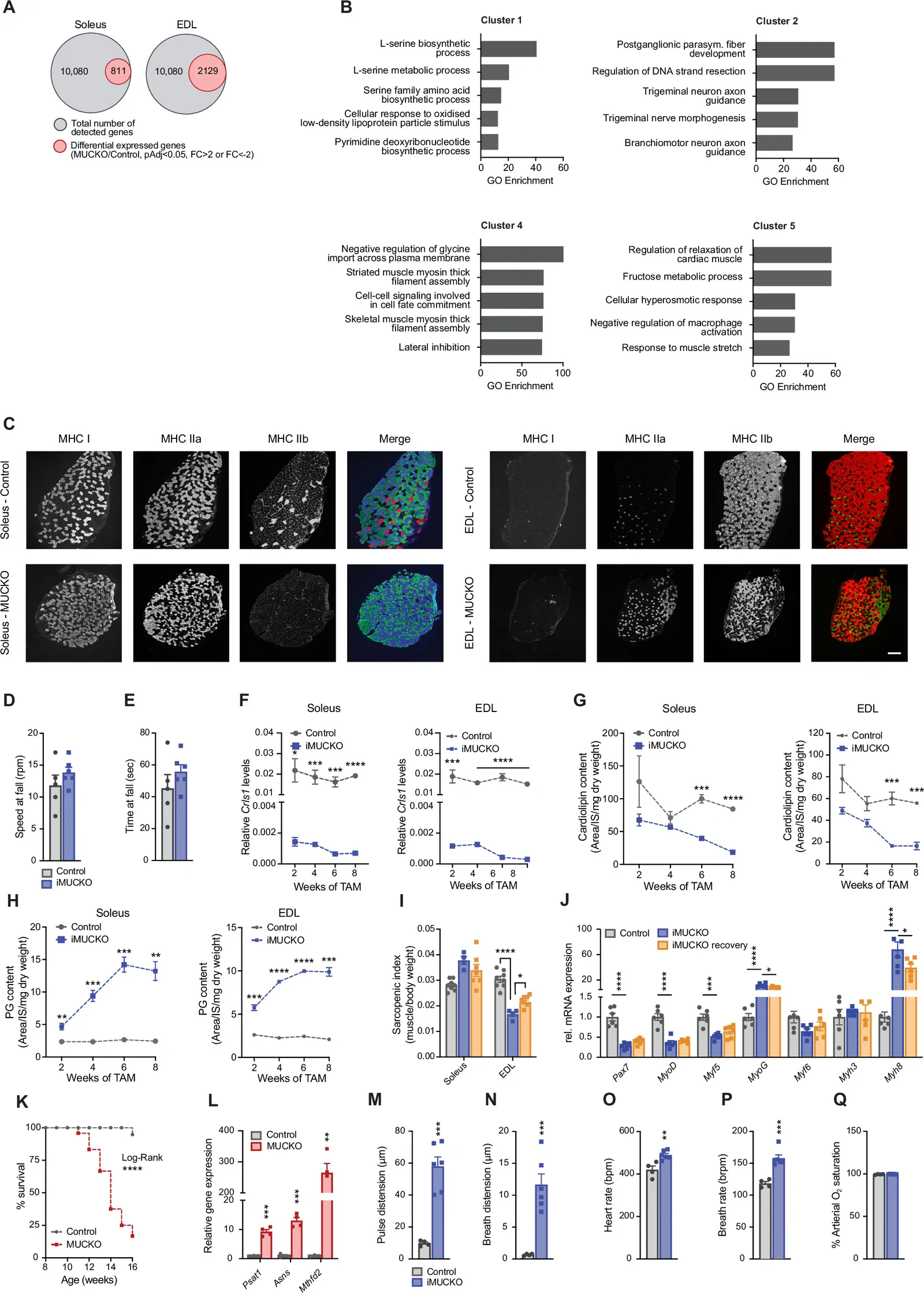
Transcriptional, fiber-type, and physiological changes in Crls1 KO mice. (**A**) Summary of global changes from RNA-sequencing analysis of Soleus and EDL from MUCKO mice and control littermates and (**B**) corresponding GO term enrichment analysis of k-means cluster 1, 2, 4, and 5 (*n* = 3). (**C**) Immunohistochemical fiber-type specific staining (representative images, *n* = 6 per group). Greyscale single channel images for MHC I, MHC IIa, MHC IIb. Coloured merge: MHC I – blue, MHC IIa – green, MHC IIb – red. Absence of staining (black): MHC IIx (scale bar = 100 μm). (**D**) Speed and (**E**) time at fall comparing iMUCKO and control mice 5 weeks post *Crls1* KO induction (*n* = 5 control, 6 iMUCKO). (**F-H**) Changes in (F) *Crls1* gene expression, (G) cardiolipin level, and (H) PG level during a time course of tamoxifen-induced *Crls1* ablation in iMUCKO mice compared to control littermates (*n* = 3 per time point). (**I-J**) Sarcopenic index and (I) expression of myogenic regulatory factors (J) of Soleus and EDL muscle in control, iMUCKO, and iMUCKO recovery animals (I: *n* = 8 control, 4 iMUCKO, 7 iMUCKO recovery; J: *n* = 6). (**K**) Mortality rate in control and MUCKO animals. (**L**) Relative gene expression of *Psat1*, *Asns*, and *Mthfd2* in diaphragm of control and MUCKO animals (*n* = 3 control, 4 MUCKO). (**M-Q**) (M) pulse distension, (N) breath distention, (O) heart rate, (P) breath rate, and (Q) percent arterial oxygen saturation in iMUCKO and control animals 5 weeks post *Crls1* KO induction (*n* = 4 control, 6 iMUCKO). Data are presented as mean ± SEM, *p < 0.05; **p < 0.01; ***p < 0.001; ****p < 0.0001. Source Data

We next profiled the global transcriptomes of SOL and EDL from MUCKOs and control littermates. Loss of CL significantly changed (adjusted *P* value (*P*_Adj_) < 0.05) expression of 2,129 genes in EDL and 811 genes in SOL by more than twofold (fold change (FC) > 2 or FC < −2) (Extended Data Fig. 3a). *K*-means clustering identified six differentially regulated clusters (Fig. 2a), two of which were characterized by gene expression patterns where *Crls1* ablation caused EDL to adopt a profile similar to that of SOL muscle by inducing (cluster 3) or suppressing (cluster 6) transcription. Pathway enrichment (Fig. 2b) in both clusters revealed that EDL from MUCKO mice undergoes a global fast- to slow-fiber-type switch. Accordingly, analysis of the promoter regions of the differentially expressed genes in MUCKO muscles uncovered a significant enrichment of binding motifs of myogenic transcription factors (Fig. 2c). Upregulated genes in cluster 1 indicate enhanced serine and pyrimidine biosynthesis, cluster 2 reflects activation of neurogenic and axon guidance pathways, cluster 4 shows reduced expression of genes involved in myosin filament assembly and muscle differentiation, and cluster 5 suggests suppression of pathways linked to fructose metabolism and osmotic or mechanical stress responses (Extended Data Fig. 3b). These clusters thus reflect coordinated metabolic and structural remodeling driven by CL deficiency but unaffected by fiber-type differences.

Histological staining confirmed this remodeling, revealing a significant decrease in the most glycolytic type IIb fibers along with a concomitant increase in the more oxidative IIa and IIx subtypes (Fig. 2d,e and Extended Data Fig. 3c). We additionally mapped the proteomes of EDL and SOL from MUCKO versus control mice. Consistent with the glycolytic-to-oxidative switch, the EDL proteome was more dramatically impacted by CL deficiency with 1,380 proteins changed (FC > 1.5 or FC < −1.5 between MUCKO and control littermates compared with 281 proteins in SOL) (Fig. 2f). Protein levels of structural and contractile factors corroborated the transcriptional patterns and revealed a clear, global shift in MUCKO EDL from a glycolytic toward a slow-contracting oxidative profile (Fig. 2g). Notably, the cross-sectional area of fibers revealed a decrease in individual fiber area, consistent with the observed reduced tissue size (Fig. 2h and Fig. 1h). However, the total number of fibers was unchanged in MUCKO EDL and SOL, indicating fiber size and not cell death as the main driver of atrophy (Fig. 2I). Thus, loss of CL restructures the composition of skeletal muscle by shifting glycolytic fibers toward smaller oxidative fibers, ultimately mirroring the atrophy and muscle-type switch observed in human aging^6,7^.

Importantly, these effects of CL deficiency were not due to developmental defects as inducible knockout of *Crls1* in the skeletal muscle of adult mice (hereby referred to as iMUCKO) (Fig. 2j) produced similar phenotypes on body weight and fiber-type-specific patterns in skeletal muscle size (Fig. 2k,l). Notably, functional impairments preceded overt changes in muscle force production. Subsequently, 5 weeks after induction of *Crls1* deletion, grip strength measurements already revealed a significant reduction in muscle strength (Fig. 2m), whereas coordination and balance as well as ex vivo measurements of tetanic and specific force generation remained unaffected at this time point (Fig. 2n,o and Extended Data Fig. 3d,e). In the iMUCKO model, *Crls1* mRNA was ablated within 2 weeks following the onset of the knockout (Extended Data Fig. 3f). However, a decrease in CL levels was not observed until 4 weeks later (Extended Data Fig. 3g), which is in line with the remarkably long half-life of CL^11^. By contrast, levels of the CL precursor, phosphatidylglycerol, had already significantly accumulated after 2 weeks (Extended Data Fig. 3h), which was more consistent with the timeframe of *Crls1* depletion. The iMUCKO model additionally enabled the investigation of the consequences of ‘rescuing’ *Crls1* expression following the initial genetic ablation. When tamoxifen administration was ceased in a subset of iMUCKOs (referred to as ‘iMUCKO recovery’), we observed that *Crls1* expression was modestly but significantly reestablished (Fig. 2p). This modest recovery elevated CL levels from 27% to 67% compared with controls and could initiate a reversal of the dramatic weight loss and skeletal muscle atrophy (Fig. 2q–s and Extended Data Fig. 3I).

Given the irreversibility of Cre‑dependent excision, we investigated the potential contribution of muscle stem cells to the recovery of *Crls1* expression and CL levels in iMUCKO mice following cessation of tamoxifen. We found that markers of satellite cells and early myogenic commitment, including *Pax7*, *Myf5*, *MyoD* and *Myf6*, were significantly reduced in the gastrocnemius from iMUCKO mice compared with controls and tended to increase in iMUCKO recovery animals (Extended Data Fig. 3j), suggesting that prolonged loss of CL is associated with depletion of the stem cell pool. In parallel, *Myog* and the neonatal myosin heavy chain (MHC) gene *Myh8* were strongly upregulated in the iMUCKO gastrocnemius and significantly decreased again in iMUCKO recovery mice (Extended Data Fig. 3j), consistent with active regeneration and remodeling of myofibers under chronic CL deficiency that subsides as *Crls1* levels are restored. iMUCKO muscles thus undergo continual replenishment with non-Cre‑recombined (that is, *Crls1*‑expressing) myonuclei from satellite cells during sustained CL loss, which progressively taxes the stem cell pool. Tamoxifen withdrawal prevents further *Crls1* excision in newly contributed nuclei and thereby permits gradual restoration of the skeletal muscle CL content.

Rescuing CL ablation also completely reversed the premature mortality observed in iMUCKOs (Fig. 2t). Early mortality was similarly seen in the constitutive MUCKO model, with mice succumbing around 4 months after birth (Extended Data Fig. 3k). A comparable premature lethality has been reported for inducible skeletal muscle-specific depletion of the other conical mitochondrial membrane lipid phosphatidylethanolamine, attributed to respiratory rather than cardiac dysfunction^24^. *Crls1* expression was significantly reduced in the diaphragm muscle but not in heart tissue (Extended Data Fig. 1g), accompanied by increased expression of mitochondrial stress response genes (Extended Data Fig. 3l). Analysis of iMUCKO mice 6 weeks after KO induction revealed elevated pulse amplitude and respiratory excursion, together with increased heart and breathing rates, while arterial oxygen saturation remained preserved (Extended Data Fig. 3m–q), suggesting diaphragm dysfunction as a primary driver of premature mortality.

**Fig. 3:**
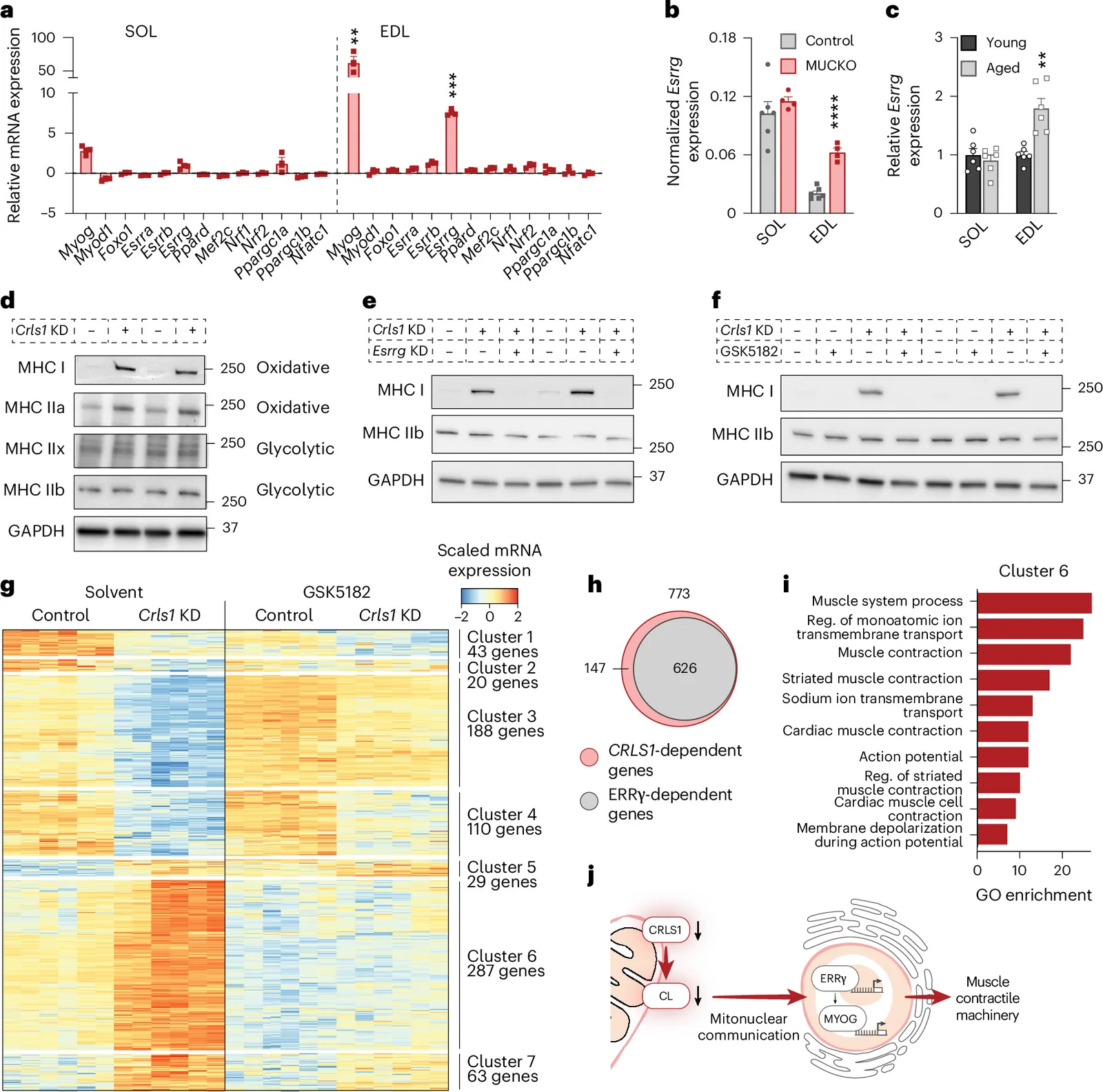
CL-dependent fiber-type shift is prompted through nuclear receptor ERRγ. **a**, Gene expression of fiber-type remodeling factors in SOL and EDL muscle (MUCKO/control) (*n* = 3). **b**, Normalized *Esrrg* expression in SOL and EDL from MUCKO and control littermates (*n* = 6 control, 4 MUCKO). **c**, *Esrrg* expression in aged compared with young mice (*n* = 6). **d**–**f**, Representative western blots showing changes in MHC types I and IIa (oxidative fiber types) and IIx and IIb (glycolytic fiber types) (GAPDH, loading control) in differentiated C2C12 myotubes upon *Crls1* knockdown (**d**), double knockdown of *Crls1* and *Esrrg* (**e**) and ERRγ inhibition (GSK5182) (**f**) (representative of three independent experiments). Positions of molecular weight markers (kDa) are indicated to the right of the blots. **g**, Hierarchical cluster analysis of global transcription profiling of control and *Crls1* knockdown myotubes treated with solvent or ERRγ inhibitor GSK5182 (*n* = 6). **h**, Significantly regulated genes upon *Crls1* knockdown (*Crls1* KD/control, FC > 2 or FC < −2, *P* < 0.05) (773 genes, red circle) and proportions of ERRγ-dependent (*Crls1* KD/control + GSK5182, non-significant, gray circle) (626 genes) and ERRγ-independent (*Crls1* KD/control + GSK5182, FC > 2 or FC < −2, *P* < 0.05) (147 genes) transcription changes. **i**, GO term enrichment analysis of cluster 6 from RNA sequencing. **j**, Schematic depicting CL-dependent mitonuclear communication inducing adaptations in the muscle contractile machinery through ERRγ and MYOG. Data are presented as mean ± s.e.m. *P < 0.05; **P < 0.01**;** ***P < 0.001; ****P < 0.0001. Source data

**Extended Data Fig. 4:**
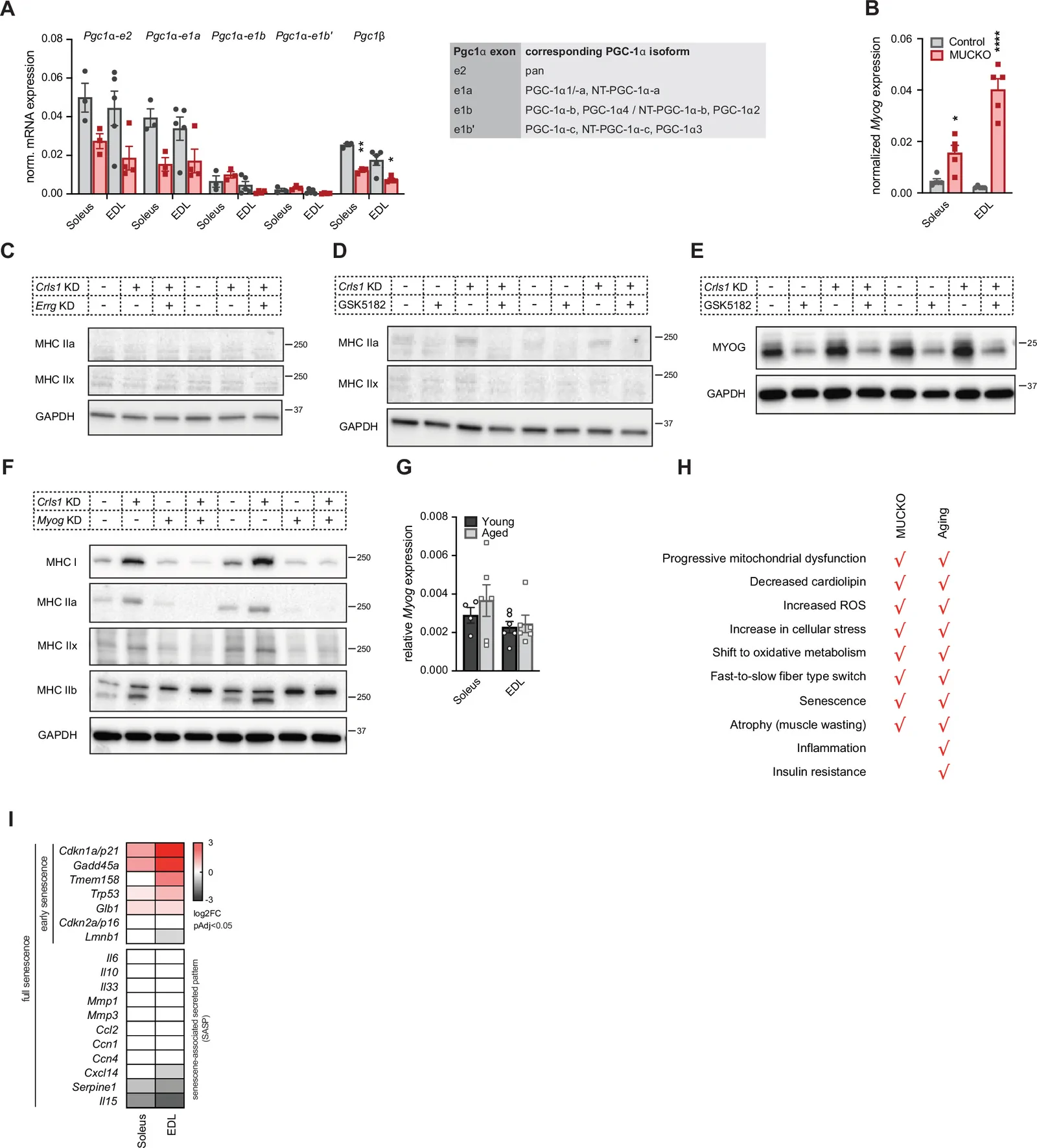
MYOG-driven fiber-type shift and senescence signatures in MUCKO muscle. (**A**) Analysis of individual *Pgc1a* exon expression in Soleus and EDL muscle from control and MUCKO animals (*n* = 5 control, 4 MUCKO). Corresponding PGC1a isoforms depicted in the grey box. (**B**) *Myog* expression level in Soleus and EDL from MUCKO mice and control littermates (n = 5). (**C-E**) Representative western blots showing changes in myosin heavy chain (MHC) types IIa (oxidative fiber-types) and IIx (glycolytic fiber-types) or MYOG protein levels (GAPDH; loading control) in differentiated C2C12 myotubes upon double knockdown of *Crls1* and *Esrrg* (C), and ERRg inhibition (GSK5182) (D-E) (representative of 3 independent experiments). (**F**) Representative western blot showing changes in MHC types I, IIa, IIx, and IIb in differentiated C2C12 myotubes upon double knockdown of *Crls1* and *Myog* (representative of 3 independent experiments). (**G**) *Myog* expression in Soleus and EDL from young (9 weeks) and aged (24 month) mice (*n* = 6). (**H**) Overview showing skeletal muscle aging hallmarks and their presence in MUCKO mice. (**I**) Transcriptional changes (log2FC, pAdj<0.05) of early and full senescence marker in Soleus and EDL muscle comparing MUCKO and control littermates (*n* = 3). Data are presented as mean ± SEM, *p < 0.05; **p < 0.01; ***p < 0.001; ****p < 0.0001. Source Data

We next compared expression of canonical transcriptional regulators of muscle fiber-type remodeling in EDL and SOL from MUCKO mice and control littermates (Fig. 3a and Extended Data Fig. 4a). Two factors, myogenin (*Myog*/MYOG) and estrogen-related receptor γ (*Esrrg*/ERRγ), were significantly increased in EDL after CL depletion, whereas none of the 12 regulators were significantly changed in SOL between genotypes. Of those two factors, *Esrrg* was the only one whose mRNA levels mirrored the patterns of mitochondrial mass (that is, CS activity and COX IV staining, Fig. 1j–l) in which EDL from MUCKOs resembled the SOL from either genotype (Fig. 3b and Extended Data Fig. 4b). We also found that *Esrrg* expression was increased in glycolytic EDL in aged mice (Fig. 3c), in line with a transcriptomic study in humans that identified *ESRRG* among the most significantly upregulated genes in skeletal muscle during healthy aging^25^.

Given that genetic and pharmacological manipulation of ERRγ is fully sufficient to promote an oxidative fiber-type switch^26–28^, we tested a potential causal role of ERRγ in CL-dependent remodeling. Consistent with our in vivo findings, ablation of *Crls1* in cultured myotubes was sufficient to selectively increase myosin heavy chain (MHC) I and IIa, but not IIx or IIb (Fig. 3d), indicating that CL depletion cell autonomously promotes a prooxidative fiber-type shift. However, the induction of MHCI following loss of CL was completely blocked by either small interfering RNA-mediated knockdown or pharmacological antagonism of ERRγ transcriptional activity (Fig. 3e,f and Extended Data Fig. 4c,d). Given the transcriptional changes in *Myog* mRNA also observed in the MUCKO muscle (Fig. 3a), we next examined whether *Myog* regulation was linked to CL–ERRγ signaling. Pharmacological inhibition of ERRγ in myotubes markedly reduced *Myog* expression (Extended Data Fig. 4e), indicating that *Myog* is dependent on ERRγ activity and placing ERRγ upstream of *Myog*. Consistent with this hierarchy, suppression of *Myog* also abrogated the *Crls1*-dependent induction of oxidative fiber-type markers (Extended Data Fig. 4f), showing that the CL-driven fiber-type switch requires *Myog*. Notably, in contrast to CL levels and *Esrrg* mRNA, *Myog* expression was not transcriptionally altered in SOL or EDL muscle from aged mice (Extended Data Fig. 4g). These data suggest that changes in CL and ERRγ signaling are the critical events for early, cell-autonomous remodeling during physiological aging. We further globally profiled the transcriptomes of control and *Crls1* knockdown myotubes that were treated with vehicle or ERRγ antagonist (Fig. 3g). Of the 773 genes that were significantly regulated (FC > 2 or FC < −2, *P* < 0.05) by loss of *Crls1*, 626 were dependent on ERRγ (Fig. 3h). This included the largest group of genes, cluster 6, which showed a pattern similar to MHCI protein, whereby CL depletion increased expression and this increase was blunted by ERRγ antagonism. Pathway analysis of genes in cluster 6 revealed a significant enrichment for muscle contractile machinery (Fig. 3i), highlighting the targeted impact of the CL–ERRγ axis on skeletal muscle function. Our data show that loss of inner membrane lipid CL is capable of cell autonomously driving the age-related fiber-type shift through retrograde mitochondria-to-nucleus (mitonuclear) signaling via the nuclear receptor, ERRγ, and MYOG (Fig. 3j). Taken together, our collective findings show that loss of CL and its synthase, CRLS1, are conserved signatures of aging in mice and humans. Mimicking this phenomenon by ablating *Crls1* in young mice was fully sufficient to causally promote several aging hallmarks, including an ERRγ-dependent glycolytic-to-oxidative fiber-type switch, and thus, identifying CL deficiency as a potential driver of aging (Extended Data Fig. 4h,i).

### Loss of CL triggers global metabolic reprogramming to fuel antioxidant defense

**Fig. 4:**
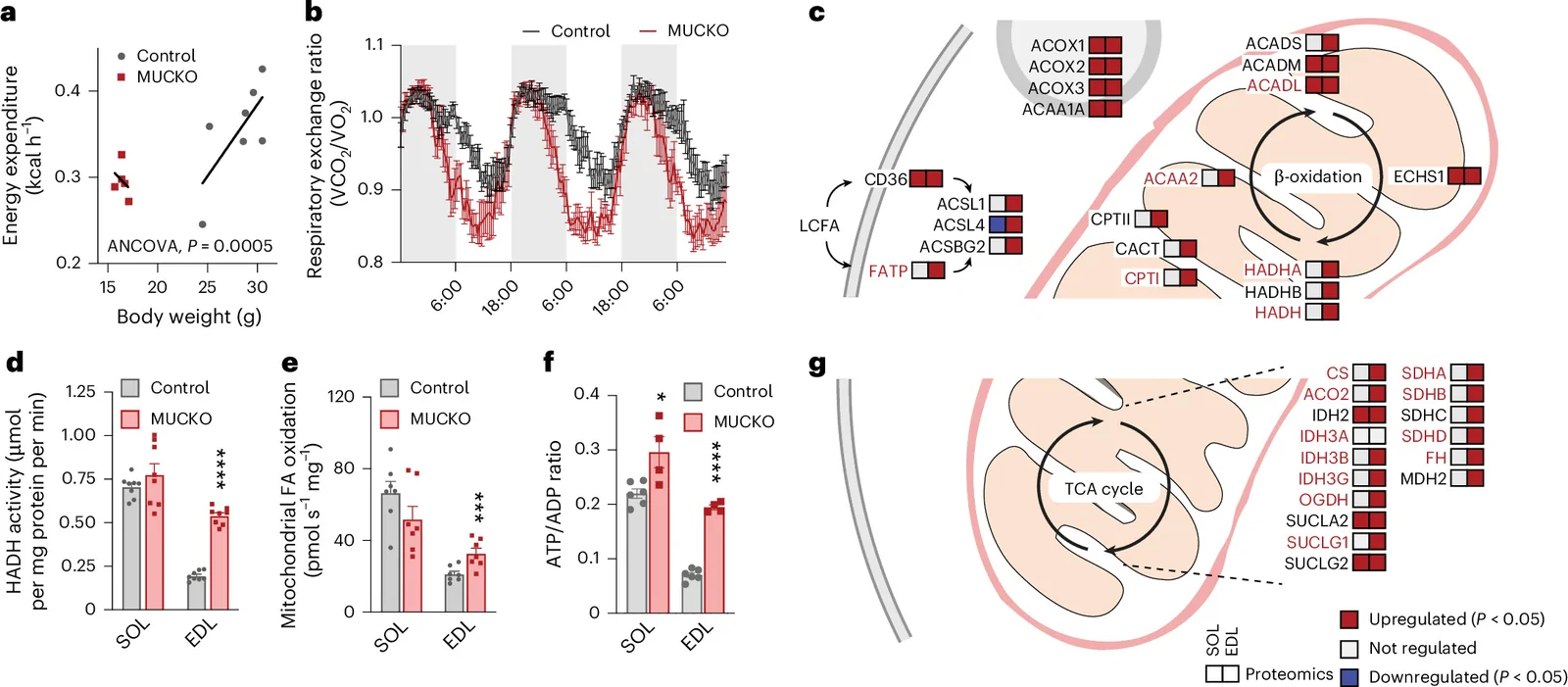
CL loss drives switch toward oxidative lipid metabolism. **a**,**b**, Energy expenditure (*n* = 7 control, 5 MUCKO) (**a**) and respiratory exchange ratio (volume (V) of CO_2_/VO_2_) comparing MUCKO mice and control littermates (*n* = 7 control, 5 MUCKO) (**b**). **c**, Changes in fatty acids using proteins (left, SOL; right, EDL; red, upregulated; blue, downregulated; *P* < 0.05) (*n* = 4 control, 5 MUCKO). Red font, ERRγ binding within promotor region (± 2,000 bp from TSS). **d**,**e**,**f**, HADH activity (*n* = 8) (**d**), mitochondrial fatty acid oxidation (*n* = 7) (**e**) and ATP/ADP ratio comparing SOL and EDL from MUCKO and control littermates (*n* = 6 control, 4 MUCKO) (**f**). **g**, Changes in TCA cycle proteins from SOL and EDL from MUCKO mice and control littermates (left, SOL; right, EDL; red, upregulated; blue, downregulated; *P* < 0.05). Red font, ERR binding within promotor region (± 2,000 bp from TSS). Data are presented as mean ± s.e.m. *P < 0.05; **P < 0.01; ***P < 0.001; ****P < 0.0001. Source data

**Extended Data Fig. 5:**
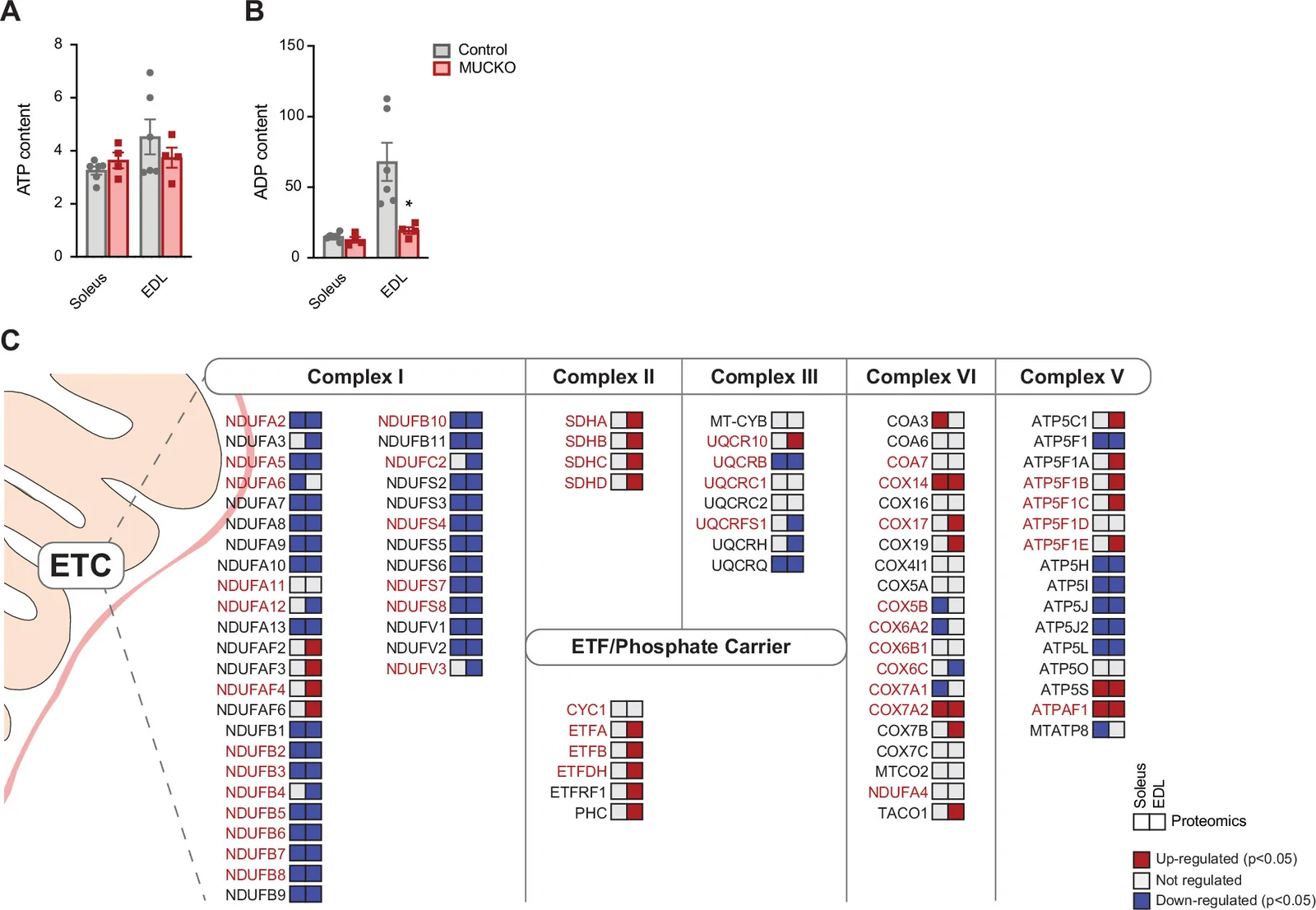
Nucleotide levels and respiratory complex remodeling in MUCKO muscle. (**A**) ATP and (**B**) ADP content in Soleus and EDL comparing control and MUCKO animals (*n* = 6 control, 4 MUCKO). (**C**) Changes in respiratory complex proteins in Soleus and EDL from MUCKO mice and control littermates (left: soleus, right: EDL; red: up-regulated, blue: down-regulated; p < 0.05) (*n* = 4 control, 5 MUCKO). Red font: ERRg binding within promotor region (+/- 2000 bp from TSS). Data are presented as mean ± SEM, *p < 0.05; **p < 0.01; ***p < 0.001; ****p < 0.0001. Source Data

**Extended Data Fig. 6:**
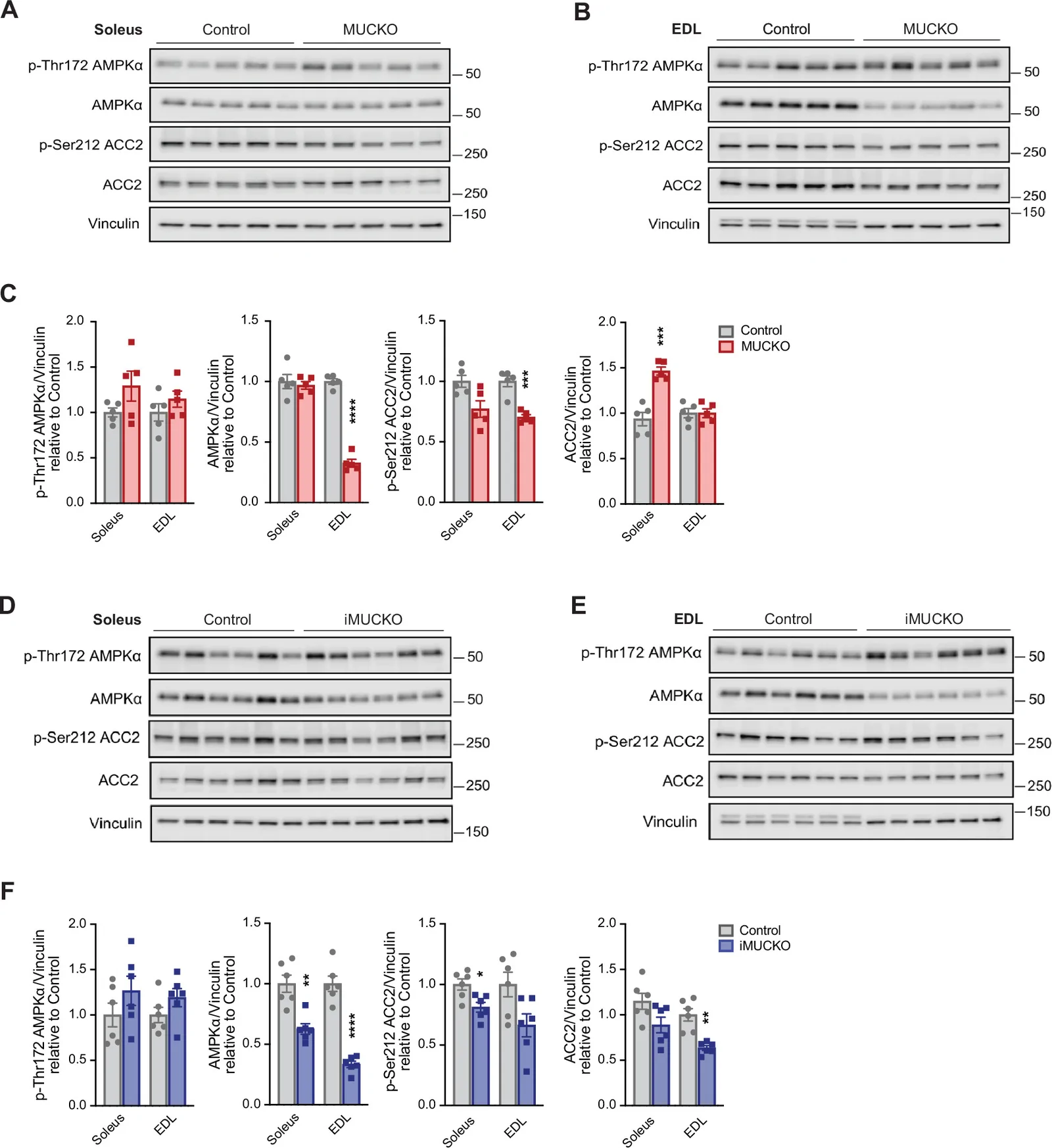
AMPK pathway activation in MUCKO and iMUCKO muscle. (**A-F**) AMPK pathway activation analysis showing representative western blots and corresponding quantifications from Soleus and EDL muscle comparing (A-C) control and MUCKO (*n* = 5) and (D-F) control and iMUCKO animals (*n* = 6). Data are presented as mean ± SEM, *p < 0.05; **p < 0.01; ***p < 0.001; ****p < 0.0001. Source Data

We next sought to explain why aging and the loss of CL would elicit an ERRγ-dependent fiber-type shift favoring oxidative muscle, assessing fiber-type-specific metabolism and whole-body energy homeostasis. In line with the oxidative shift, MUCKO mice showed elevated energy expenditure and a reduced respiratory exchange ratio, signifying a systemic preference for lipid fuels, compared with control littermates (Fig. 4a,b). Accordingly, CL depletion in EDL increased proteins involved in the uptake, intracellular trafficking, and peroxisomal and mitochondrial oxidation of fatty acids (Fig. 4c). This induction in lipid-utilizing proteins in the classically glycolytic EDL was functionally reflected in the specific activity of the fatty acid oxidation (FAO) enzyme, 3-hydroxyacyl-CoA dehydrogenase (HADH) (Fig. 4d) as well as overall mitochondrial lipid-catabolizing capacity (Fig. 4e). Consistent with a shift toward oxidative metabolism, CL deficiency increased the ATP-to-ADP ratio, an effect driven exclusively by a reduction in ADP content that was more pronounced in EDL than in SOL muscle (Fig. 4f and Extended Data Fig. 5a,b). Given these FAO changes, we next investigated how CL depletion impacted the machinery linking lipid catabolism to electron transport. FAO supplies the electron transport chain through multiple entry points, including direct NADH/FADH_2_ input to complex I and the electron transfer flavoprotein (ETF) pathway, respectively, and acetyl-CoA entry into the TCA cycle. Loss of CLs markedly increased all components of the ETF shuttle in the EDL as well as the entire cassette of TCA cycle enzymes, notably including succinate dehydrogenase (that is, complex II) (Fig. 4g and Extended Data Fig. 5c). Interestingly, most subunits of complex I were decreased in both MUCKO muscle types (Extended Data Fig. 5c) suggesting that CL-depleted SOL and EDL rely more on ETF and complex II-mediated entry into the respiratory chain. Notably, by reanalyzing published muscle ERRγ chromatin immunoprecipitation sequencing (ChIP-seq) data^27^, promotor-proximal binding of ERRγ was observed at genes encoding the majority of the mitochondrial lipid oxidation machinery, including components of the TCA cycle, electron transport chain and ETF shuttle, identifying these genes as potential direct transcriptional targets of ERRγ (red highlight, Fig. 4c,g and Extended Data Fig. 5c). ERRγ binding was also evident at the *Myog* locus, validating its regulation as a downstream target in Extended Data Fig. 4e. These findings extend the role of ERRγ in CL-dependent fiber-type remodeling to encompass coordinated metabolic adaptations. Given AMPK’s central role in driving the glucose-to-lipid oxidation shift, we assessed its activation in both knockout models but found no evidence of enhanced AMPK activity based on phosphorylation of AMPKα or its substrate acetyl-CoA carboxylase 2 (Extended Data Fig. 6a–f). Total AMPKα protein was instead reduced in *Crls1*-deficient EDL, suggesting that CL-dependent metabolic remodeling occurs independently of canonical AMPK signaling. Collectively, our findings reveal that CL deficiency comprehensively restructures the functional architecture of glycolytic muscle to promote lipid utilization.

**Fig. 5:**
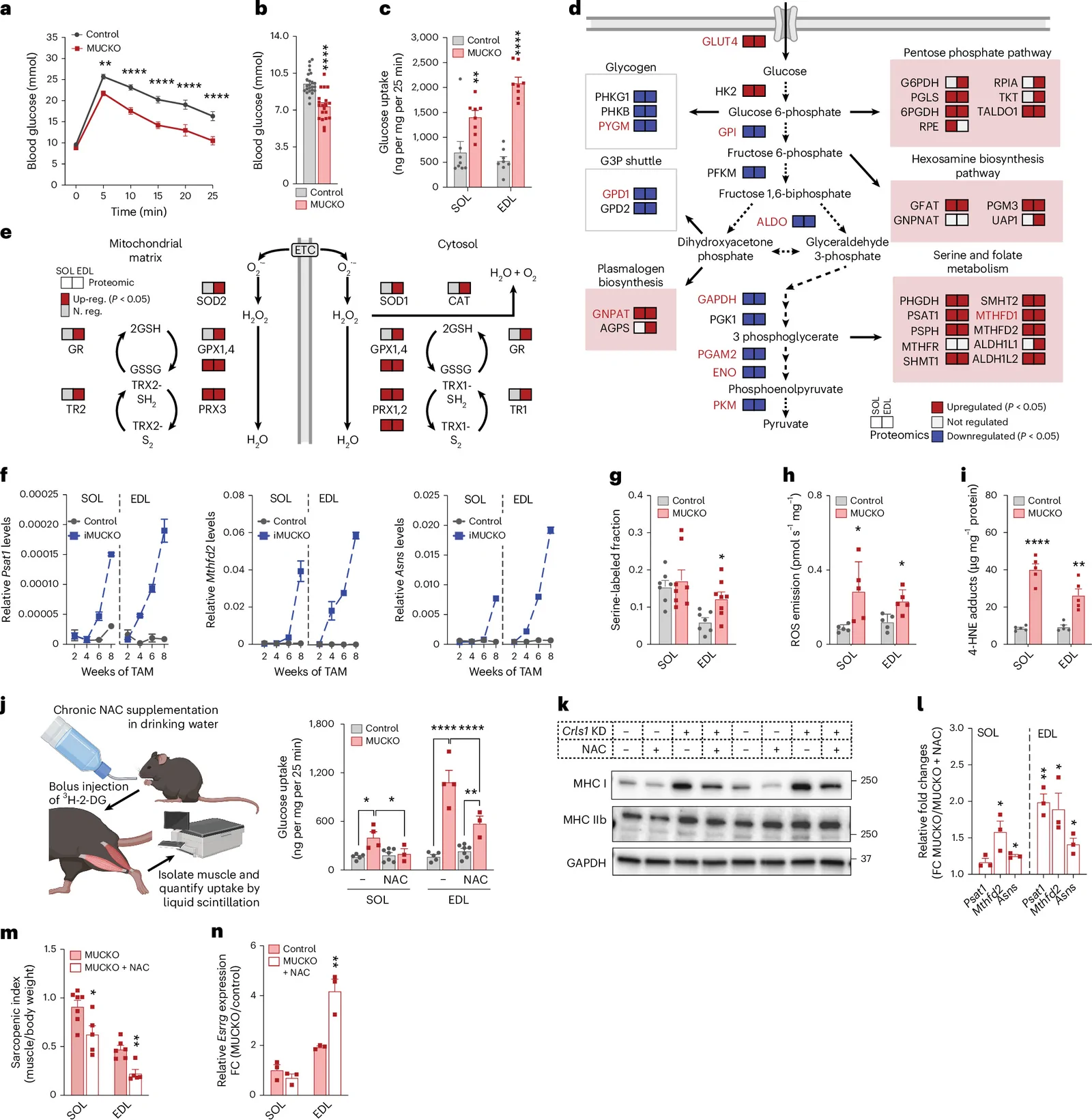
CL deficiency triggers global metabolic reprogramming to fuel antioxidant defenses. **a**,**b**,**c**, Intravenous glucose tolerance test (*n* = 8) (**a**), fasting blood glucose level (*n* = 24 control, 20 MUCKO) (**b**) and in vivo glucose uptake (**c**) in SOL and EDL from MUCKO and control littermates (*n* = 8). **d**,**e**, Proteomic analysis of glycolysis and glycolytic shunts (**d**) and of mitochondrial and cytosolic antioxidant defense pathways (**e**) (left, SOL; right, EDL; red, upregulated; blue, downregulated; *P* < 0.05) (*n* = 4 control, 5 MUCKO). Up-reg., upregulated; N. reg., not regulated. Red font, ERRγ binding within promotor region (± 2,000 bp from TSS). **f**, Time course of *Psat1*, *Mthfd2* and *Asns* expression in SOL and EDL after tamoxifen-induced *Crls1* deletion in iMUCKO mice compared with tamoxifen-treated control littermates (*n* = 3 per time point). **g**, Radiolabeled serine after glucose tracing in SOL and EDL (*n* = 7 control, 8 MUCKO). **h**,**i**, Mitochondrial ROS emission (**h**) and 4-HNE adduct quantification from SOL and EDL of MUCKO mice and control littermates (*n* = 5) (**i**). **j**, Schematic outlining in vivo glucose uptake studies where NAC was administered in the drinking water (10 mg ml^−1^) for 8 weeks and respective in vivo glucose uptake in SOL and EDL from MUCKO mice and control littermates (*n* = 5 control, 4 MUCKO, 7 control + NAC, 3 MUCKO + NAC). **k**, Representative western blot showing changes in MHC types I (oxidative) and IIb (glycolytic) protein levels (GAPDH, loading control) in differentiated C2C12 myotubes upon *Crls1* knockdown and NAC supplementation (representative of three independent experiments). **l**, Relative fold changes of *Psat1*, *Mthfd2* and *Asns* expression comparing MUCKO and MUCKO + NAC supplementation in SOL and EDL muscle (*n* = 4 MUCKO, 3 MUCKO + NAC). **m**,**n**, Sarcopenic index (*n* = 6 MUCKO, 5 MUCKO + NAC) (**m**) and *Esrrg* gene expression of SOL and EDL muscle from MUCKO mice treated with or without NAC (*n* = 3) (**n**). Data are presented as mean ± s.e.m. *P < 0.05; **P < 0.01; ***P < 0.001; ****P < 0.0001. Illustrations in **j** created in BioRender; Finger, F. https://biorender.com/x9e21xd (2026). Source data

**Extended Data Fig. 7:**
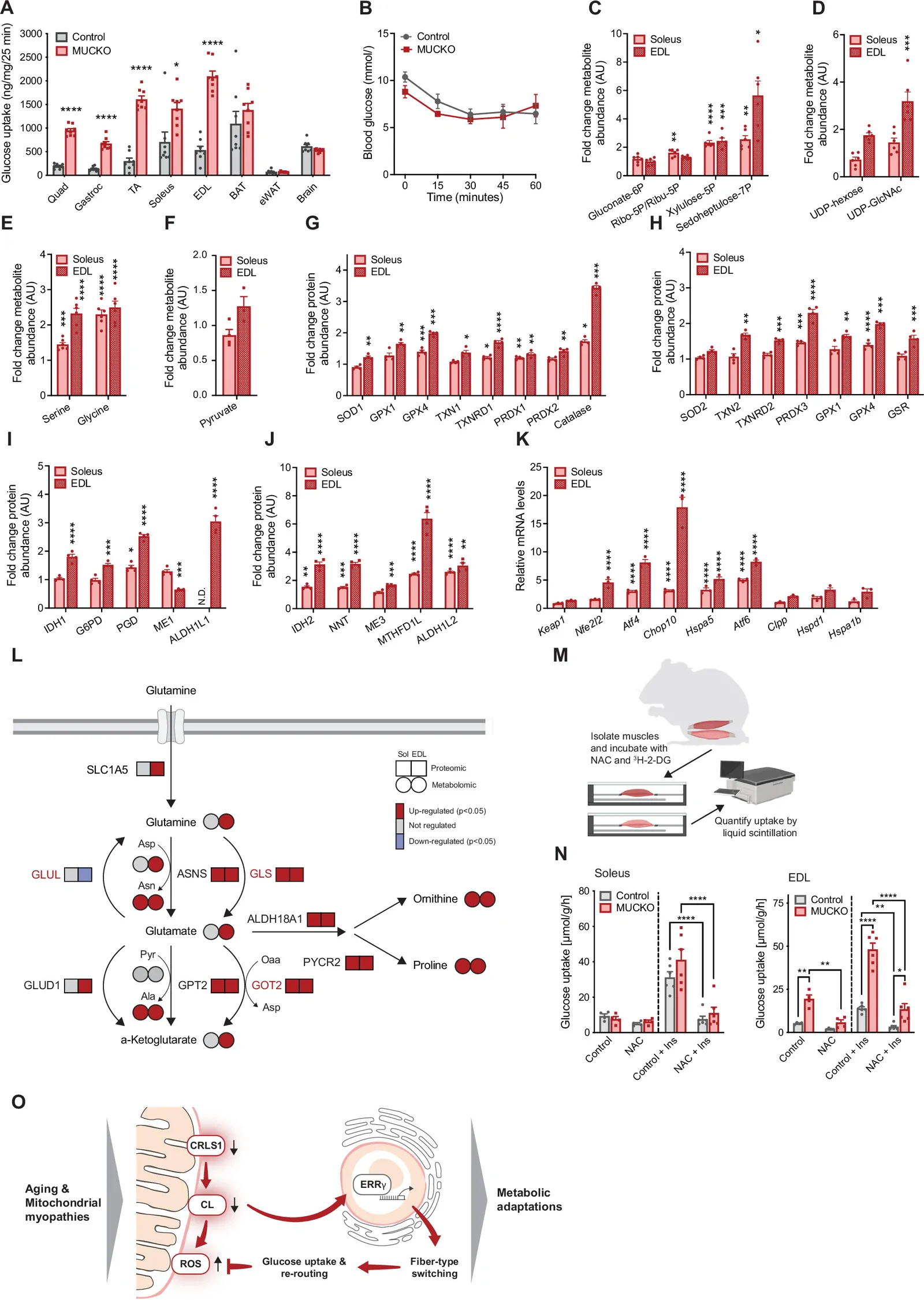
Glucose metabolism and antioxidant rewiring in MUCKO muscle. (**A**) *In vivo* glucose uptake in skeletal muscle, adipose, and brain from MUCKO and control littermates (*n* = 8). (**B**) Intraperitoneal insulin tolerance test comparing MUCKO and control littermates (*n* = 6 control, 4 MUCKO). (**C-F**) Changes in glucose shunt pathway intermediates and pyruvate in Soleus and EDL (FC (MUCKO/Control) (*n* = 6 control, 4 MUCKO). (**G-K**) Changes in protein levels of, (G) cytosolic and (H) mitochondrial antioxidant defense, (I) cytosolic and (J) mitochondrial NADPH production (*n* = 4 control, 5 MUCKO), and, (K) transcriptional changes in oxidative stress markers from MUCKO mice and control littermates (*n* = 3). (**L**) changes in proteins and metabolites in glutaminolysis from MUCKO mice and control littermates (square: proteomic, circle: metabolomic; left: soleus, right: EDL; red: up-regulated, blue: down-regulated; p < 0.05) (*n* = 4 control, 5 MUCKO). Red font: ERRg binding within promotor region (+/- 2000 bp from TSS). (**M**) Schematic outlining ex vivo glucose uptake studies in the presence of 20 mM N-acetylcysteine (NAC) with or without 10 mU/mL insulin. (**N**) *Ex vivo* glucose uptake in Soleus and EDL from MUCKO mice and control littermates (*n* = 6). (**O**) Summary graphic showing how age and myopathy-associated decreases in cardiolipin trigger an ERRg-dependent oxidative fiber-type shift and global metabolic reprogramming to fuel antioxidant defense. Data are presented as mean ± SEM, *p < 0.05; **p < 0.01; ***p < 0.001; ****p < 0.0001. Illustration in **m**created in BioRender; Finger, F. https://biorender.com/0jop38w (2026). Source Data

We next investigated how increased FAO capacity affected glucose metabolism. Unexpectedly, MUCKO mice were markedly more glucose tolerant compared with control littermates (Fig. 5a,b). Improved glucose control was primarily driven by increased glucose consumption in skeletal muscles from MUCKO mice (Fig. 5c), whereas glucose uptake remained unchanged in other peripheral organs, such as brown and white adipose tissues (Extended Data Fig. 7a). In contrast to other models of muscle mitochondrial dysfunction^29^, CL deficiency had no effect on sensitivity to an insulin bolus (Extended Data Fig. 7b). We next used our muscle-type-specific proteomic mapping to delineate the fate of the increased glucose consumed by MUCKO muscles. Interestingly, whereas the first enzyme in glycolysis, hexokinase 2, was significantly increased (Fig. 5d), all subsequent downstream enzymes were reduced (Fig. 5d). Moreover, protein changes in glycolytic shunt pathways suggested increased activity in the hexosamine biosynthesis, pentose phosphate pathway, serine metabolism and the folate cycle (Fig. 5d), which collectively fuel antioxidant response pathways^30^. Proteomic evidence for glucose rerouting was reinforced by metabolomic profiling showing an accumulation of UDP-GlcNAc, pentose phosphate pathway intermediates and serine–folate cycle metabolites, while levels of pyruvate, the end product of glycolysis, remained unchanged (Extended Data Fig. 7c–f). In addition, nearly every component of the reactive oxygen species (ROS) defense system (Fig. 5e) and an array of oxidative stress markers were increased in the CL-deficient muscle (Extended Data Fig. 7g–k).

Temporal resolution of inducible CL depletion revealed a fiber-type-specific increase in one carbon and glutamine metabolism factors (Fig. 5f), preceding in EDL by several weeks relative to SOL, thereby highlighting the demand in glycolytic muscle to reroute glucose from complete catabolism at an earlier stage. *Psat1*, *Mthfd2* and *Asns* are canonical mitochondrial integrated stress response target genes, reflecting glycolytic muscle’s higher sensitivity to CL loss (Fig. 5f). Consistently, isotopic tracing showed increased glucose-to-serine conversion in EDL from CL-depleted animals (Fig. 5g). Our proteomic and metabolomic analyses also indicate increased glutaminolysis enzymes and intermediates, through which glutamine could anaplerotically support continued TCA capacity under conditions of potential carbon deficit from glycolytic intermediate shunting (Extended Data Fig. 7l).

Consistent with the need for increased antioxidant defense, we observed elevated mitochondrial ROS production and increased lipid peroxidation in MUCKO mice (Fig. 5h,i). To determine how ROS influences MUCKO glucose consumption, we assessed glucose disposal in vivo with and without administration of the antioxidant *N*-acetylcysteine (NAC). NAC significantly blunted glucose uptake in both EDL and SOL from MUCKOs (Fig. 5j), suggesting that CL-deficiency-derived ROS itself stimulates glucose uptake. In isolated muscles, MUCKO EDL consumed significantly more glucose than controls even without insulin, an effect amplified by insulin stimulation (Extended Data Fig. 7m,n). NAC dramatically dampened glucose uptake triggered by either CL depletion or insulin stimulation (Extended Data Fig. 7n). In isolated SOL, loss of CL did not impact basal glucose uptake although NAC did abolish insulin-stimulated uptake (Extended Data Fig. 7n). These findings establish ROS as an underlying cause in driving glucose uptake in CL-deficient muscles. Notably, high NAC concentrations can directly interfere with insulin signaling^31^. Thus, the role of ROS in the glucose uptake induced by CL deficiency remains to be fully resolved.

Reducing the free radical burden in CL-depleted myotubes with NAC also blunted the induction of the oxidative fiber-type marker, MHCI (Fig. 5k), suggesting ROS contributes to CL-mediated mitonuclear signaling. Consistently, NAC supplementation resulted in a significantly lower sarcopenic index in both SOL and EDL, alongside further activation of the mitochondrial integrated stress response, enhanced glucose shunting and increased *Esrrg* expression (Fig. 5l–n). These findings indicate that chronic antioxidant treatment, probably by limiting glucose availability for metabolic and redox-supporting pathways, exacerbates rather than ameliorates the detrimental consequences of CL loss. Taken together, our data support a model in which age- and myopathy-associated declines in CL synthesis cell autonomously activate an ERRγ-dependent oxidative fiber-type switch and global metabolic reprogramming to sustain antioxidant defense, a compensatory response that cannot be rescued by nonspecific ROS scavenging (Extended Data Fig. 7o).

## Discussion

One of the major changes skeletal muscle undergoes with age is a shift in fiber composition from fast-twitch (type II) to slow-twitch (type I) fibers^7,32^, a phenomenon also observed in cancer cachexia^33^. While the cellular mechanisms underlying this switch remain unresolved, multiple upstream events contribute: physical inactivity and impairment of neuromuscular junctions collectively increase frailty and promote sarcopenia^34^, and since fast-twitch fibers are more susceptible to atrophy, this would ultimately favor slower, fatigue-resistant fibers^1^. Compensatory adaptations, including mitochondrial signals shaping intracellular responses and mitokine secretion modulating systemic homeostasis, also contribute^35–38^. Resolving primary causal events among this complex network of inputs remains key to understanding how and why our muscles change with age. Yet such knowledge is essential to enable pinpointing which underlying pathways should be targeted to improve the quality of aging (that is, health span).

Mitochondria are inextricably linked to skeletal muscle aging, yet whether dysfunction precedes or results from age-associated decline is unclear. Genetic mouse models with mitochondrial perturbations in skeletal muscle, including disruptions in respiration, fusion and fission machinery^29^, autophagy^39^, apoptosis^40^, calcium loading^41^, acetylcarnitine metabolism^42^ and phosphatidylethanolamine synthesis^24^, have provided numerous insights, and CL is linked to nearly all these features. Yet despite CL’s central importance to mitochondrial function^9–13^ and contributions to disease^17^, investigating its causal role in aging has proven challenging, partly owing to the limitations of the previously available in vivo tools. Existing models that more directly disrupt CL and IMM integrity instead rely on whole-body deletion of upstream (PTPMT1, embryonic lethal^43^) or downstream (TAFAZZIN^44^ or ALCAT1^45^) enzymes, or on tissue-specific models with broader disruption of general lipid processes such as long-chain acyl-CoA synthesis^46^ or very-long-chain fatty acid elongation^47^. Thus, our constitutive (MUCKO) and inducible (iMUCKO) models of CL depletion serve as distinct and versatile tools for investigating skeletal muscle function in aging and disease.

CL levels decline during aging in both oxidative and glycolytic muscle fibers (Fig. 1a), possibly owing to reduced expression of biosynthetic enzymes, diminished intrinsic enzymatic activity at the protein level or both. Whether this reduction is a cause or consequence of age-associated processes has remained unresolved^19^. Here we find a significant decrease in the key CL synthesis enzyme, *Crls1/CRLS1*, in mice and human aging and demonstrate that skeletal muscle-specific depletion of CL synthesis in young mice recapitulates multiple, tissue-specific aging hallmarks on a markedly accelerated timescale (Extended Data Fig. 4h). Future studies will need to dissect the relative contributions of satellite cell-mediated myogenesis versus repair and remodeling of existing myofibers in the context of CL loss. These contributions are likely to be dynamic and may shift over time from acute regenerative responses toward progressive stem cell impairment and altered repair capacity.

Aging is characterized by mitochondrial dysfunction^4^, increased ROS production^48^, cellular stress^49^, senescence^50^, muscle atrophy^51^ and fast- to slow-fiber-type shifts^6^, all in MUCKO mice by 3 months of age and in iMUCKO mice beginning approximately 6 weeks after *Crls1* deletion. More advanced pathological features of aging, including inflammation^52^ and insulin resistance^53^, were not observed at the ages studied, per the absence of a senescence-associated inflammatory signature (Extended Data Fig. 4I) and preserved insulin responsiveness in vivo and ex vivo (Extended Data Fig. 7b,m,n). We further show that the weight loss, muscle atrophy and early mortality of *Crls1* loss in the iMUCKO model are reversible even with only modest CL-level recovery, raising the intriguing possibility of boosting *Crls1* or CL levels in aged muscle as an intervention point. Future studies will be required to investigate this using *Crls1* gain of function in the context of aged mice. These findings position the MUCKO and iMUCKO models as a powerful platform to interrogate causal mechanisms underlying early-to-middle-stage skeletal muscle aging, while raising the question of whether prolonged CL depletion ultimately precipitates late-stage pathological aging phenotypes.

Our study establishes CL deficiency as a causal event of muscle fiber-type switching and other aging hallmarks. While the global impact of decreased CL includes both pathological and protective elements, the latter, previously unknown mechanism reveals a promising intervention point for mitigating age-related decline. We discovered that CL synthesis loss initiates mitonuclear signaling through ERRγ to cell autonomously drive the fast- to slow-fiber-type switch and compensatory metabolic adaptations. This ERRγ-dependent program likely reflects adaptive remodeling promoting oxidative fiber characteristics in surviving fibers, rather than directly driving selective loss of atropy-prone fast glycolytic fibers. ERRγ is a particularly appealing target, as its expression in human skeletal muscle correlates with healthy aging^25^ and it has a well-established role in beneficial oxidative exercise adaptations^26,54^. Genetic overexpression of ERRγ alone is sufficient to elicit the fast to slow shift and increase exercise performance, even without a canonical coactivator, PGC-1α^27^. As distinct PGC-1α isoforms can elicit divergent adaptations, we assessed their expression and found several isoforms reduced (Extended Data Fig. 4a), suggesting a PGC-1α-independent role for ERRγ. While multiple transcriptional regulators, including the closely related ERRα, can drive overlapping exercise-induced programs, our data identify ERRγ as the predominant mediator of age- and myopathy-associated fiber-type adaptation. Future study will need to resolve how CL loss signals to ERRγ and, more broadly, whether other disruptions to mitochondrial features or activities similarly converge on ERRγ.

The functional consequences of this ERRγ-dependent fiber-type switch on ROS protection offer a compelling mechanistic rationale for the long-standing paradox of why aging skeletal muscle would instigate an oxidative adaptation: CL-deficiency-driven remodeling dramatically alters the metabolic and enzymatic landscape to globally support antioxidant defense. Underscoring this, muscle-specific ERRγ ablation increases ROS production and impairs muscle function^28^. We also found that remodeling confers the ability to robustly increase glucose uptake in response to elevated ROS, complementing the enzymatic reprogramming and increasing the volume of glycolytic intermediates siphoned to antioxidant pathways. ROS has previously been shown to contribute to exercise-induced glucose uptake^55,56^, and patients with Barth syndrome show elevated ROS^57^ and increased glucose uptake^58,59^ in skeletal muscle, hinting at parallels between physiological and disease-related adaptations. While the exact mechanism linking oxidative stress to glucose handling post-CL depletion is unclear, ROS plays a key role in intracellular redox signaling^38^ and ERRγ has been reported to function as an ROS sensor in cancer^60^. Collectively, our findings suggest that skeletal muscle remodeling is not merely a consequence of aging but an active, adaptive response protecting cellular integrity.

## Methods

### Human samples

Biospecimens used in our study were obtained from a previously published human cohort^61^, which comprised only men who underwent an extreme endurance cycling intervention. Only pre-exercise (baseline) samples from this cohort were used in the current analyses. Race, ethnicity and other demographic characteristics of the participants were collected and are reported in the original publication^61^; the present analyses used de-identified samples without additional stratification by race or ethnicity. No new participants were recruited for this study, and all samples were obtained from this existing, previously published cohort, with recruitment procedures and informed consent described in that original publication. Collection of human samples was approved by the local ethics committee at the University of Copenhagen (H-16049145), as reported in the original publication. Our study constitutes a secondary analysis of these stored samples, conducted under the same ethical approval.

### Animals

All animal experiments were conducted in full compliance with applicable national and institutional ethical regulations. Mouse experiments performed in Denmark were approved by the Danish Animal Research Council under license number 2018-15-0201-01441. Mouse experiments involving aged C57BL/6J cohorts were approved by the Ethics Committee for Animal Welfare of the State Office of Environment, Health and Consumer Protection of the Federal State of Brandenburg, Germany.

Conditional knockout *Crls1* mice (*Crls1*^*f/f*^) were generated by flanking exon 4, which contains a part of the CDP-alcohol phosphatidyltransferase domain, with *loxP*sites as previously described^23^. Given the importance of CL in skeletal muscle, we crossed the *Crls1*^*f/f*^ mice with animals expressing Cre recombinase under the control of the human skeletal actin promoter to generate a skeletal muscle-specific *Crls1* knockout (MUCKO). Cohorts were bred crossing *Crls1*^*f/f*^ *Cre*^*−*^ mice with *Crls1*^*f/wt*^ *Cre*^*+*^ mice. Experiments were conducted in both male and female mice between 4 weeks and 16 weeks of age. To generate tamoxifen-inducible skeletal muscle-specific *Crls1* knockout mice (iMUCKO), we crossed *Crls1*^*f/f*^ mice with animals expressing MerCreMer double fusion protein under the control of the human skeletal actin promoter^62^. Knockout was induced by oral gavage with 2 mg tamoxifen (Sigma-Aldrich, T5648) in 100 µl corn oil daily for 3 consecutive days, followed by once weekly gavage for the duration of the respective study. Mice were group housed on a 12:12 h light–dark cycle (lights on at 6 a.m. and off at 6 p.m.) at room temperature in an enriched environment. Mice were fed ad libitum with a standard chow diet (1310 Altromin or SAFT D30).

Male C57BL/6J mice were used to compare young and aged cohorts. Animals were maintained as a local specific pathogen-free colony, originally obtained from Charles River Laboratories. Mice were housed under controlled environmental conditions (20 ± 2 °C, 12 h–12 h light–dark cycle) and provided ad libitum access to a standard diet (Ssniff).

### Gene expression analysis: RT qPCR and RNA sequencing

RNA was extracted using TRI Reagent (Sigma-Aldrich) and subsequent isopropanol precipitation from snap-frozen tissues. cDNA synthesis was carried out on 500–1,000 ng RNA using a High Capacity cDNA Reverse Transcription kit (Applied Biosystems). cDNA synthesis was performed on a Mastercycler Pro (Eppendorf) according to the manufacturerʼs protocol. Gene expression levels were assessed with SYBR Green (Primerdesign)-based quantitative PCR LightCycler (Roche). Gene expressions were analyzed with the DDC_T_ method and normalized to the housekeeping gene *36b4*. Oligonucleotide sequences are listed in Supplementary Table 1.

For generation of RNA-sequencing libraries, polyadenylated mRNA was isolated from 500 ng of total RNA by incubation with oligo-dT beads and prepared according to the manufacturer’s protocol (TruSeq 2, Illumina). Control and MUCKO samples (EDL and SOL) were sequenced on an Illumina HiSeq 1500 platform, with reads mapped to the mouse reference genome (mm9) using STAR^63^, tag directories generated with HOMER^64^ and exon reads counted using intron (i)RNA-seq^65^. Young and aged samples (EDL and SOL), along with a rerun of the MUCKO/control comparison for cross-platform compatibility, underwent 52-bp paired-end sequencing on a NovaSeq 6000, processed by the single-cell omics platform at the Novo Nordisk Foundation Center for Basic Metabolic Research: sample-demultiplexed FASTQ files were generated from BCL files using bcl-convert (v.4.0.3), and gene-level count matrices were produced via the nf-core RNA-sequencing pipeline (v.3.12), which trims reads with Trim Galore (v.0.6.7), deduplicates UMIs with UMI-tools (v.1.1.4), maps reads to GRCm38/GRCh38 using STAR (v.2.7.9a) and quantifies gene counts with Salmon (v.1.10.1). For both datasets, normalization and identification of differentially expressed genes were performed using DESeq2 (ref. ^65^), visualized by K-means or hierarchical clustering, and pathway analysis was conducted via GO enrichment (Fisher’s exact test, false discovery rate (FDR) correction, *P* < 0.05)^66,67^.

### ChIP-seq analysis

Raw ERRγ ChIP-seq reads were obtained from the Sequence Read Archive database, accession number SRP110311. Paired-end reads were adapter-trimmed using Trim Galore (v.0.6.7) and aligned to the mm9 reference genome using BWA-MEM (v.0.7.18), followed by sorting, indexing and filtering with BamTools (v.2.5.2) and SAMtools (v.1.15.1). Blacklist regions were excluded and peaks were called using MACS3 (v.3.0.1) with default parameters^68–72^.

### Mitochondrial respiration

Mitochondrial respiration was measured in permeabilized skeletal muscle fibers using high-resolution respirometry (Oxygraph-2k, Oroboros Instruments) as previously described^73^. Briefly, all respiratory measurements were made in duplicates in a hyperoxygenated environment ([O_2_], 450–200 nmol ml^−1^) to avoid potential oxygen limitations of the respiration. Skeletal muscle fibers were permeabilized with saponin (50 µg ml^−1^) for 30 min in BIOPS buffer followed by a 2 × 10 min wash in mitochondrial respiration medium (mir05). State 2 leak respiration was assessed after addition of malate (2 mM), glutamate (10 mM) and pyruvate (5 mM). Next, NADH-linked respiration was measured by adding ADP (5 mM) and MgCl^2+^ (3 mM) followed by cytochrome *c* (10 mM) to evaluate membrane integrity. Oxidative phosphorylation capacity with simultaneous electron input of complex I and II was assessed with succinate (10 mM). Finally, the complex III inhibitor antimycin A (1 mM) was added to shut down electron transfer to complex IV. In separate experiments, complex II respiration were assessed by addition of ADP (5 mM) and MgCl^2+^ (3 mM), followed by rotenone (1 mM) and succinate titrations (0.1–10 mM). FAO capacity was tested by addition of malate (0.1 mM), followed by octanoyl-carnitine (0.25 mM) then ADP (5 mM) and MgCl^2+^ (5 mM). All respiratory rates were measured in mir05 buffer (containing 110 mmol l^−1^ sucrose, 60 mmol l^−1^ potassium lactobionate, 0.5 mmol l^−1^ EGTA, 3 mmol l^−1^ MgCl_2_·6H_2_O, 20 mmol l^−1^ taurine, 10 mmol l^−1^ KH_2_PO_4_, 20 mmol l^−1^ HEPES, 1 g l^−1^ BSA, pH 7.1) at 37 °C.

### Mitochondrial isolation and Seahorse OCR measurements

Mitochondria were isolated from mouse skeletal muscle by differential centrifugation in ice‑cold isolation buffer containing sucrose, EDTA and protease inhibitors, followed by low‑ and high‑speed centrifugation to obtain a mitochondrial pellet, which was resuspended, kept on ice and quantified by protein assay. For oxygen consumption rate (OCR) measurements, mitochondria were diluted in assay medium containing pyruvate, malate and glutamate (PMG) and seeded into a Seahorse XF/XFe analyzer using the isolated‑mitochondria protocol. OCR was measured after sequential injections of ADP, rotenone, succinate and antimycin A to assess basal respiration, complex I‑driven (CI), combined complex I + II‑driven (CI + II) and complex II‑driven (CII) respiration before antimycin A addition, with OCR at each stage calculated as the average of two consecutive measurements and expressed per µg mitochondrial protein^74,75^.

### Mitochondrial ROS production

Mitochondrial-derived ROS were measured in permeabilized SOL and EDL muscles from MUCKO mice and control littermates. The method has been described previously^76^ and is based on the H_2_O_2_-dependent oxidation of Amplex Red to resorufin, a reaction catalyzed by horseradish peroxidase (HRP). In short, all measurements were made in duplicates in a hyperoxygenated environment ([O_2_], 200–450 nmol ml^−1^) in saponin-permeabilized (50 mg ml^−1^) fibers. The protocol focused on evaluating the ROS production from complex I and maximally ROS production from complex I + II by titrations of succinate. Before baseline measurement, Blebbistatin (25 mM) for inhibiting muscle contraction, SOD (90 U ml^−1^) for converting O_2_^*−*^ to H_2_O_2_ and Amplex Red (50 mM) were added, after which baseline was determined by addition of HRP (2 U ml^−1^). State 2_I_ ROS emission was determined after addition of malate (2 mM), glutamate (10 mM) and pyruvate (5 mM). Subsequent titration of succinate (1 mM, 3 mM, 8 mM and 16 mM) was used to estimate maximal state 2_I+II_ ROS production. State 3_I+II_ were obtained by addition of ADP (5 mM) and MgCl^2+^ (3 mM), and finally antimycin A (2.5 mM) were added. A 0.1-mM H_2_O_2_ was added before each step to correct for the decreased sensitivity. ROS measurements were carried out using buffer Z (containing 1 mmol l^*−*1^ EGTA, 5 mmol l^*−*1^ MgCl_2_, 105 mmol l^*−*1^ K-MES, 30 mmol l^*−*1^ KCl, 10 mmol l^*−*1^ KH_2_PO_4_, 5 g l^*−*1^ BSA, pH 7.1) at 37 °C.

### CS and HADH

CS and HADH activities were measured as previously described with one small modification: a TissueLyser II (Qiagen) was used for homogenization^77^. In short, snap-frozen muscles were dissected free of visible blood and connective tissue and were homogenized in 300–500 ml homogenization buffer (0.3 M K_2_HPO_4_, 10% BSA, pH 7.7) and incubated in 0.1% Triton X for 15 min. CS activity was measured at 415 nm by the CoA-SH-mediated conversion of DTBN to TNB in a solution containing 100 mM acetyl-CoA, 0.5 mM NAD, 1 mM sodium malate, 8 mg ml^−1^ malate dehydrogenase (1,200 U mg^−1^, Boehringer Mannheim), 2.5 mM EDTA and 10 mM Tris–HCl (pH 8.0). HADH was measured at 340 nm by the consumption of NADH in a solution containing 50 mM acetoacetyl-CoA, 35 mM NADH, 0.06 mM EDTA and 40 mM imidazole (pH 7.0). The enzyme activity was determined per gram of dry weight/by protein content. Both CS and HADH activities were measured at 25 °C.

### Glucose homeostasis

Glucose and insulin tolerance tests were assessed following an intraperitoneal injection of 2 g dextrose (D9434, Sigma-Aldrich) or 0.75 units insulin (Novo Nordisk, Actrapid) per kg lean mass following a 4-h fast. Blood glucose was measured from a tail vein at baseline and 15 min, 30 min, 45 min and 60 min for insulin tolerance test and also 120 min for glucose tolerance test postinjection using Contour XT blood glucose meters and test strips (Bayer). Glucose tolerance test and insulin tolerance test were performed 1 week apart in the same cohort of mice.

In vivo glucose uptake was performed in 12–13-week-old males after 4 h of fasting. Mice were anesthetized with pentobarbital (75 mg kg^−1^ body weight, intraperitoneal injection), and glucose uptake was assessed in response to a single retro-orbital injection of 20% glucose (1 g kg^−1^ lean mass) spiked with ^3^H-2-deoxyglucose tracer (^3^H-2-DG, 0.33 µCi kg^−1^ lean mass; PerkinElmer, NET549A005MC). Mice were monitored on heating pads (37 °C) during the experiment. To estimate the decay of ^3^H-specific activity in plasma, tail vein blood (25 µl) was sampled before injection (baseline) and every 5 min postinjection for 25 min in total. Blood glucose was measured at each time point using Contour XT blood glucose meters and test strips (Bayer). Blood samples were immediately mixed with 60 µl 0.3 N Ba(OH)_2_ followed by addition of 60 µl 0.3 N ZnSO_4_. Samples were centrifuged for 5 min at 14,000 *g* and 50 µl supernatant was transferred into 3 ml Ultima Gold (PerkinElmer, 6013329). After 25 min postinjection, mice were euthanized by cervical dislocation, and tissues were collected. Tissue samples were homogenized in 800 µl ice-cold lysis buffer (10% glycerol, 1% IGEPAL, 50 mmol l^−1^ HEPES, 150 mmol l^−1^ NaCl, 10 mmol l^−1^ NaF, 1 mmol l^−1^ EDTA, 1 mmol l^−1^ EGTA, 20 mmol l^−1^ sodium pyrophosphate, 2 mmol l^−1^ sodium orthovanadate, 5 mmol l^−1^ nicotinamide, 4 mmol l^−1^ thiamet G and protease inhibitors (Sigma-Aldrich), pH 7.4). Around 150 µl of the homogenate was deproteinized with 300 µl 0.3 N Ba(OH)_2_ (Sigma-Aldrich, B4059) and 300 µl 0.3 N ZnSO_4_ (Sigma-Aldrich, Z2876) to precipitate the 2-DG-6-phosphate. Another 150 µl was deproteinized in 600 µl 4.5% perchloric acid (Sigma-Aldrich, 244252). Then, 500 µl of supernatant was transferred into 6 ml of Ultima Gold (PerkinElmer, 6013329) and counted for 10 min per sample. The glucose uptake was calculated as previously described^78^. Briefly, baseline 2-deoxy-ᴅ-glucose (2-DG) count was subtracted from blood sample count and recalibrated to account for the dilution factor. AUC was calculated for both the glucose measurements and the 2-DG counts at each time point, and a conversion factor was calculated (AUC, disintegrations per minute (DPM) mg^−1^ glucose). The Ba(OH)_2_/ZnSO_4_-precipitation supernatant counts were subtracted from the perchloric acid extract to obtain the 2-DG trapped in tissues; the results were recalibrated to account for the tissue dilution factor and normalized to tissue weight and the conversion factor.

### Jugular vein catheterization

The mice were anesthetized with isoflurane in oxygen (2.5–3% for induction, 1.5–2% for maintenance). Following induction of anesthesia, the animals were placed on a 37 °C heat pad for thermal support, and this was maintained throughout the surgical procedure. Carprofen (5 mg kg^−1^; Norodyl Vet, Scanvet) was injected subcutaneously for preoperative analgesia, and the incision sites were prepared by shaving and disinfection with 70% ethanol followed by 0.5% chlorhexidine in 80% ethanol. Local anesthesia was administered preoperatively by subcutaneous injection of a lidocaine and bupivacaine cocktail at the incision sites (10 mg kg^−1^; Xylocaine, AstraZeneca, and 5 mg kg^−1^; Marcaine, Orifarm). Following preparation, the mice were moved to the surgical field and covered with sterile drapes. All surgical procedures were performed under strict aseptic conditions. Following a right ventrolateral neck incision, the external jugular vein was exposed by blunt dissection, and two 7-0 silk ligatures were placed around it. The rostral ligature was tied for occlusion of the vein, and a 2-Fr PU catheter (C20PU-MJV1458; Instech) was inserted 1 mm rostral from the clavicle and secured with both ligatures. The catheter was externalized through a dorsal incision in the interscapular region using a Vascular Access Button (VABM1B/25; Instech), and the catheter was locked with heparinized saline (200 IU ml^−1^). The incisions were closed with resorbable suture (7-0 Vicryl, Ethicon), anesthesia was discontinued and the mice were moved to clean cages for recovery. Following surgery, the mice had voluntary access to thermal support for 24 h from a 37 °C heat pad placed under approximately half of the cage.

### Voluntary running

Mice were housed individually in cages with a running wheel and they were allowed free access to the running wheel for 3 weeks. Each wheel was equipped with a magnetic switch and counter. Daily running distance and time spent in the running wheel were logged every morning. Single-housed sedentary mice were kept in similar cages without running wheels. The mice were kept at a 12:12 light–dark cycle (light at 6 a.m. to 6 p.m. and dark from 6 p.m. to 6 a.m.).

### Rotarod and grip strength measurements

Motor coordination was assessed using an accelerating rotarod (4–40 rpm over 200 s). The mice were subjected to three trials, each ending when the animal fell from the rod, and the best latency to fall was used for analysis.

Grip strength was measured using a force transducer with a horizontally oriented grid. The mice grasped the grid with both forepaws and hind limbs and were gently pulled backward by the tail at a constant speed until grip release. Only valid pulls showing resistance and correct limb use were included. Three measurements were performed at 30-s intervals, averaged and normalized to body weight.

### Ex vivo muscle contractions

SOL and EDL muscle was rapidly dissected, as previously described^79,80^, and incubated into warmed (30 °C) and oxygenated (95% O_2_, 5% CO_2_) Krebs–Ringer buffer (117 mM NaCl, 4.7 mM KCl, 2.5 mM CaCl_2_, 1.2 mM KH_2_PO_4_, 1.2 mM MgSO_4_, 24.6 mM NaHCO_3_, 0.1% bovine serum albumin and 5 mM glucose) in a myograph system (820MS DT). Before the experiment, optimal muscle length was determined by finding the length of maximal isometric twitch response (pulse voltage 14 V, pulse width 0.5 ms). Single-twitch assessment was conducted (3 stimuli of 14 V and 0.5 ms pulse width separated by >30 s and then averaged) to determine maximal twitch force. For tetanic contractions (pulse voltage 14 V, pulse width 0.2 ms, interval 6.50 ms, frequency 150 Hz, 75 pulse counts in each train, 500 ms total train time, 5,000 ms pause between trains for a total of 110 trains), the area under the curve was measured to assess total force production by summating the products of peak force and contraction duration from each tetanic contraction. Absolute force measurements were normalized to muscle cross-sectional area to find the specific force (muscle mass divided by the product of the muscle density coefficient (1.06 g cm^−^^3^), optimal muscle length and the muscle-specific fiber length coefficient (0.71 for SOL and 0.44 for EDL^81^)).

### Indirect calorimetry

Indirect calorimetry was performed using Phenomaster Home Cage Systems (TSE). Mice were acclimated in training cages for 3–4 days before initiation and transferred to the TSE cabinets 2–3 days before baseline measurements. The cabinets were set to a 12:12 light–dark cycle (light at 6 a.m. to 6 p.m. and dark from 6 p.m. to 6 a.m.). All data were recorded in 5-min intervals. Data collection was integrated into TSE software. Data analysis followed the consensus guide given in a previous study^82^.

### Body composition analysis

Mouse body composition was measured using nuclear magnetic resonance technology with an EchoMRI 4-in-1 body composition analyzer.

### Pulse oximetry measurements

Physiological parameters were measured in sedated mice using the MouseOx pulse oximetry system (STARR Life Sciences) with a paw-mounted sensor. Arterial oxygen saturation (SpO_2_), heart rate, breath rate and pulse and breath distension were recorded noninvasively from the plethysmographic signal. The mice were maintained on a heating pad throughout the measurements to preserve body temperature. Data were continuously acquired and analyzed using the manufacturer’s software.

### TEM

Samples were fixed with 2% v/v glutaraldehyde in 0.05 M sodium phosphate buffer (pH 7.2). Following isolation of suitable specimen blocks, the samples were rinsed three times in 0.15 M sodium phosphate buffer (pH 7.2) and subsequently postfixed in 1% w/v OsO_4_ with 0.05 M K_3_Fe(CN)_6_ in 0.12 M sodium cacodylate buffer (pH 7.2) for 2 h. The specimens were dehydrated in graded series of ethanol, transferred to propylene oxide and embedded in Epon according to standard procedures. Sections, approximately 60 nm thick, were cut with a Ultracut 7 microtome (Leica) and collected on copper grids with Formvar supporting membranes, stained with uranyl acetate and lead citrate, and subsequently examined with a Philips CM-100 TEM (Philips), operated at an accelerating voltage of 80 kV. Digital images were recorded with an OSIS Veleta digital slow scan 2k × 2k CCD camera and the ITEM software package.

Morphometric quantification of TEM images from the EDL and SOL muscles was performed manually using Fiji ImageJ^83^. MERCS were identified as regions where the ER and outer mitochondrial membrane ran parallel and in close apposition, at a distance of less than 50 nm, and their number was quantified per mitochondrion. The percentage of mitochondrial perimeter covered by MERCS was calculated manually and normalized to total mitochondrial perimeter.

### Immunohistochemistry

#### Mitochondrial content

SOL and EDL muscles were freshly dissected and fixed as previously described. Briefly, muscles were immersed into 1.17 mg ml^−1^ procaine in Krebs buffer for 5 min followed by fixation for 4 h (30 min at room temperature and 3.5 h at 4 °C) in 2% paraformaldehyde with 0.15% picric acid. Next, the muscles were then transferred into phosphate buffer (PBS) and teased into smaller bundles, transferred to 50% glycerol in PBS (v/v) and kept at 4 °C overnight before storage at −20 °C until analysis. For staining, the muscles were teased into single fibers using fine forceps and transferred to immunobuffer (IB; 0.25% BSA, 50 mM glycine, 0.033% saponin, 0.05% sodium azide). Single fibers were stained overnight with primary anti-COXIV (16056, Abcam) antibody diluted 1:500 and anti-MHC I (Sigma-Aldrich, M8421) diluted 1:500 in IB, washed for 3 h with IB and incubated overnight at room temperature with a goat anti-rabbit conjugated to Alexa Fluor 488 and goat anti-mouse conjugated to Alexa Fluor 568 (Molecular Probes, Life Technologies) diluted both 1:400 in IB. Next day, the fibers were washed in IB for 3 h and washed with PBS before being mounted on a glass slide in Vectashield mounting medium (Vector Lab, H-1000) and covered with a coverslip number 1.5. Image acquisition was performed on a Zeiss LSM700, with a Plan-Apochromat ×63/1.4 objective. Z-stacks of minimum 15 planes from the muscle fibers’ surface were recorded in at least 15 fibers for each muscle. Voxel size was set according to Nyquist criteria (*x* =90 nm, *y* = 90 nm and *z* = 280 nm). Mitochondrial staining was segmented using the machine learning module Intellesis, and factional area was quantified using the image analysis module from the Zeiss ZEN software (Carl Zeiss).

#### MHC

SOL and EDL muscles were freshly dissected and snap frozen in isopentane cooled by dry ice and kept at −80 °C. Then, 8-µm cryo sections were fixed in a mixture of acetone and 100% alcohol (1:1) and incubated with 5% goat serum to block unspecific binding. Next, muscle sections were stained with monoclonal anti-MHC antibodies: BA-D5 (IgG2b, supernatant, 1:100) specific for MHC I, SC-71 (IgG1, supernatant, 1:100) specific for MHC IIa and BF-F3 (IgM, purified antibody, 1:100) specific for MHC IIb overnight. Type IIx fibers are not recognized by these antibodies, so all black fibers were counted as MHC IIx. Three different secondary antibodies (Jackson ImmunoResearch) were used to selectively bind to each primary antibody: goat anti-mouse IgG1, conjugated with DyLight 488 fluorophore (to bind to SC-71); goat anti-mouse IgG2b, conjugated with DyLight 405 fluorophore (to bind to BA-D5); and goat anti-mouse IgM, conjugated with DyLight 549 fluorophore (to bind to BF-F3). Following 1 h of incubation with secondary antibodies, sections were mounted with Dako fluorescent media. Images were recorded with an epifluorescence Olympus BX61 microscope equipped with an Olympus DP71 camera.

### Metabolomics

Metabolomics analyses were performed in 1–3 mg of freeze-dried EDL or SOL, following a two-phase extraction protocol^84^. Samples were disrupted using a TissueLyser II homogenizer (Qiagen; 5 min at 30 pulses s^−1^) in 425 µl water, 500 µl methanol and 175 µl internal standards mixture. Once homogenization was complete, 1,000 µl chloroform was added to each sample, followed by thorough mixing and centrifugation (5 min, 16,000 × *g*, 4 °C). The top polar phase of the two-phase system generated was then transferred to clean tubes and dried under vacuum at 60 °C. The pellets obtained after evaporation were redissolved in 100 µl methanol–water (6/4; v/v) and analyzed in an Acquity UPLC system (Waters) coupled to an Impact II ultra-high-resolution Qq-time-of-flight MS (Bruker). Chromatographic separation of metabolites was performed in a SeQuant ZIC-cHILIC column (PEEK 100 × 2.1 mm, 3 µm particle size; Merck) at 30 °C using a method consisting of a gradient running at 0.25 ml min^−1^ from 100% mobile phase B (9:1 acetonitrile–water containing 5 mM ammonium acetate, pH 6.8) to 100% mobile phase A (1:9 acetonitrile–water containing 5 mM ammonium acetate, pH 6.8) for 28 min, followed by a re-equilibration step at 100% B for 5 min. MS data were acquired over the range of 50–1,200 m/z in negative and positive ionization modes and analyzed using Bruker TASQ software (v.2.1.22.1 1065). All reported metabolite abundances were normalized to total dry weight as well as to internal standards with comparable retention times and response in the MS.

### Lipidomics

Lipidomics analyses were performed in 1–3 mg of freeze-dried EDL or SOL. Samples were disrupted using a TissueLyser II device (Qiagen; 5 min at 30 pulses s^−1^) in 1.5 ml methanol–chloroform containing 100 µl internal standard mix. After clearance by centrifugation, 1.25 ml of each homogenized sample was transferred to clean glass vials and evaporated at 45 °C under a nitrogen flow. The pellets obtained were dissolved in 150 µl chloroform–methanol (1/1; v/v) and analyzed in a Thermo Fisher Scientific Ultimate 3000 binary UPLC coupled to a Q Exactive Plus Orbitrap mass spectrometer using nitrogen as a nebulizing gas. The spray voltage was set at 2,500 V, and the capillary temperature at 256 °C. For normal-phase separation, a Phenomenex LUNA silica, 250 × 2 mm, 5 µm and 100 Å was used. Mobile phase consisted of (A) 85:15 (v/v) methanol–water containing 0.0125% formic acid and 3.35 mM ammonia and (B) 97:3 (v/v) chloroform–methanol containing 0.0125% formic acid. The LC gradient (flow rate 0.3 ml min^−1^; column temperature 25 °C) was as follows: 10% A from 0–1 min, increasing to 20% A at 4 min, 85% A at 12 min and 100% A at 12.1 min; held at 100% A from 12.1–14 min; returned to 10% A at 14.1 min and held at 10% A from 14.1–15 min. For reverse-phase separation, a Waters HSS T3 column, 150 × 2.1 mm and 1.8 µm, was used. Mobile phase consisted of (A) 4:6 (v/v) methanol–water and (B) 1:9 (v/v) methanol–isopropanol, containing 0.1% formic acid and 10 mM ammonia in both cases. The LC gradient (flow rate 0.4 ml min^−1^; column temperature 60 °C) was as follows: 100% A at 0 min, decreasing to 80% A at 1 min and 0% A at 16 min; held at 0% A from 16–20 min; returned to 100% A at 20.1 min and held at 100% A from 20.1–21 min. MS data were acquired using negative and positive ionization using continuous scanning over the range of 150–2,000 m/z. Data were analyzed using an in-house-developed pipeline written in the R programming language^84^. All reported lipid abundances were normalized to total dry weight as well as to internal standards with comparable retention times and response in the MS.

### Proteomics

#### Sample preparation and TMT labeling

The extracted proteins from muscle tissue (EDL or SOL) from each group were incubated in denaturation buffer (6 M urea, 2 M thiourea, 10 mM dithiothreitol (Sigma-Aldrich), phosphatase–protease inhibitor cocktails) for 2 h at room temperature (RT). Following incubation, the samples were diluted tenfold with 20 mM triethylammonium bicarbonate (TEAB, Sigma-Aldrich), pH 7.5, and 20 mM iodoacetamide (Sigma) was subsequently added and incubated for 20 min in the dark at RT. After incubation, 2% (w/w) trypsin was added to the samples, which were then incubated overnight at RT. Following this proteolysis, the samples were lyophilized before tandem mass tag (TMT) labeling. A total of 100 µg of sample from each group was labeled with the TMT10plex kit (Thermo Scientific) based on the quantification achieved from Qubit Fluorometric Quantitation (Thermo Scientific) (‘control’ (126, 127 C, 128 N and 128 C), ‘CRLS KO’ (129 N, 129 C, 130 N, 130 C and 131)). The TMT-labeled peptides were mixed in equal ratios and analyzed simultaneously.

#### Liquid chromatography–tandem mass spectrometry analysis

All fractions were redissolved in buffer A (0.1% TFA) and analyzed using a liquid chromatography–mass spectrometer system (LC–MS) consisting of an Easy-nLC (Thermo Scientific) and an Orbitrap Fusion Lumos MS or a QE-HF MS (Thermo Scientific). The samples were loaded directly onto a two-column system containing a 3-cm pre-column and a 17-cm analytical column (75 μm inner diameter) and separated on an analytical column packed with ReproSil-Pur C18-AQ 3 μm RP material (Dr. Maisch). The peptides were eluted using 60–120 min gradients from 1% to 40% buffer B (95% ACN, 0.1% TFA) at a flow rate of 300 nl min^−1^ and introduced into the MS instrument via nano-electrospray. For the Lumos instrument, the automatic gain control target value of 1.5 × 10^6^ ions and a maximum fill time of 50 ms were used. Each MS scan was acquired at high resolution (120,000 full width half maximum) at 200 m/z in the Orbitrap with a mass range of 400–1,400 Da. The instrument was set to select as many precursor ions as possible in 3 s between the MS analyses.

#### Protein identification and quantification

The raw MS datasets were processed for protein identification using the Proteome Discoverer (PD, v.2.3, Thermo Scientific) and subjected to database searching using both an in-house Mascot server (Matrix Science) and Sequest HT algorithm with a peptide mass tolerance of 10 ppm, fragment m/z tolerance of 0.05 Da and a FDR of 1% for proteins and peptides. All peak lists were searched against the UniProtKB/Swiss-Prot database (v.2017-07-17) of mouse sequences using the following parameters: enzyme, trypsin; maximum missed cleavages, 2; fixed modification, carbamidomethylation (C), TMT tags (K, peptide N-termini) and variable modifications, oxidation (M) and phosphorylation (S, T, Y). For relative protein quantification, the output file was exported into Microsoft Excel from PD. Only proteins with more than two unique peptides (only in a single protein) were considered for further analysis in the proteomics analysis. Protein relative expression values from the respective unique peptides were calculated by summing all peptide intensities of each protein and normalized to the number of the total intensity of each group to estimate the relative amounts of the different proteins within the sample. The in-built ANOVA test in PD was used to define differentially expressed proteins (*P*_Adj_ < 0.05 with ± 1.4-fold changes).

#### Bioinformatics analysis of proteomics data

GO annotation enrichment analysis was performed using PANTHER (http://www.pantherdb.org/) and Metacore (Clarivate analytics). These GO analyses allowed the enrichment of biological process, cellular components and signaling pathway. Protein–protein interaction analysis was searched against the STRING database (v.11.0) (https://string-db.org/). Results were produced and visualized using GraphPad Prism 8 (GraphPad Software) and Photoshop (Adobe)^85,86^.

### In vitro *Crls1* knockdown

C2C12 myoblasts were grown (high-glucose DMEM + 10% fetal bovine serum + 100 μg ml^−1^ of penicillin–streptomycin) and differentiated into myotubes (low glucose DMEM (1 g l^−1^ glucose, L-glutamine, 110 mg l^−1^ sodium pyruvate) + 2% horse serum + 100 μg ml^−1^ of penicillin–streptomycin). HEK 293T cells were maintained in high-glucose DMEM + 10% fetal bovine serum + 100 μg ml^−1^ of penicillin–streptomycin. Lentivirus-mediated knockdown of *Crls1*, *Esrrg* and *Myog* genes was performed as previously described^24^. Vectors were purchased from Sigma for shRNA targeting mouse *Crls1* (clone ID TRCN0000123937) and mouse *Esrrg* (clone ID TRCN0000027078) or cloned with *Myog* shRNA (clone ID TRCN0000428112), and Addgene for psPAX2 (ID 12260), pMD2.G (ID 12259) and scrambled shRNA plasmid (SC ID 1864). ERRγ inhibitor (GSK5182) was purchased from MedChemExpress (catalog number HY-11226A) and dissolved in DMSO at 10 mM concentration. Scramble and *Crls1* knockdown C2C12 myoblasts were treated with either GSK5182 or DMSO (vehicle) at the final concentration of 10 µM or 0.1%, respectively, during differentiation for 72 h from day 1 to day 4. NAC was purchased from Sigma and dissolved in ddH_2_O at 200 mM concentration. Scramble and *Crls1* knockdown C2C12 myoblasts were treated with either NAC or ddH_2_O (vehicle) at the final concentration of 2 mM or 1%, respectively, during differentiation for 4 days.

### Western blotting

C2C12 myotubes were homogenized and Western blotting was performed as previously described. Protein homogenates were analyzed for abundance of MHC type I (DSHB, A4.840), MHC type IIa (DSHB, SC-71), MHC type IIx (DSHB, 6H1), MHC type IIb (DSHB, BF-F3), MyoG (DSHB, F5D) and GAPDH (Cell Signaling Technology, 14C10). Blue native-PAGE analysis was performed using the following antibodies: NDUFB6 (Abcam, 21C11BC11) for complex I, UQCRC2 (Abcam, 13G12) for complex III and MT-CO1 (Thermo Fisher Scientific, 1D6E1A8) for complex IV.

#### Preparation of mouse tissue extracts for protein analysis

SOL and EDL muscles were homogenized in ice-cold lysis buffer (270 mM sucrose, 50 mM Tris–HCl (pH 7.5), 1 mM EDTA, 1 mM EGTA, 1% (v/v) Triton X-100, 20 mM glycerol-2-phosphate, 50 mM NaF, 5 mM Na_4_P_2_O_7_, 1 mM DTT, 0.1 mM PMSF, 1 mM benzamidine, 1 μg ml^−1^ microcystin-LR, 2 μg ml^−1^ leupeptin and 2 μg ml^−1^ pepstatin A) using a TissueLyser II (Qiagen) at 30 Hz for 1 min. Tissue lysates were centrifuged at 6,000 × *g* for 10 min at 4 °C, and total protein concentration in the clarified lysate was determined by a microplate reader (Hidex Sense) using Bradford reagent (23200, Thermo Fisher) and BSA as standard.

#### Immunoblotting

Protein extracts were denatured in Laemmli buffer (50 mM Tris, 0.5 mM EDTA, 2% w/v SDS, 10% w/v glycerol and 0.01% w/v bromophenol blue) at 95 °C for 5 min. Subsequently, 12–15 µg of protein was separated by SDS–PAGE on 4–20% gradient gels (Criterion TGX Stain-Free, 5678095, BioRad) using the Tris-glycine running buffer (25 mM Tris, 192 mM glycine and 0.1% w/v SDS) and transferred onto nitrocellulose membranes (926-31090, LI-COR) using Tris-glycine transfer buffer (48.2 mM Tris and 40 mM glycine). Molecular weight marker (Precision Plus protein dual color standards, Biorad 1610374) ranging from 10–250 kD was used as reference for protein sizes. Membranes were blocked in 5% skimmed milk for 1 h at RT followed by incubation in TBST (10 mM Tris–HCl (pH 7.6), 137 mM NaCl and 0.1% (v/v) Tween-20) containing 5% (w/v) BSA and primary antibody overnight at 4 °C. The membranes were extensively washed with TBST before incubation with HRP-conjugated secondary antibodies diluted 1:10,000 for 45 min. Signal detection of the blots was performed using enhanced chemiluminescence substrate (Millipore, WBKLS0500), and protein bands were visualized by Odyssey XF imaging system (LICORbio). The volume densities of the protein bands were quantified with the Image Studio Lite Ver 5.2 software. Specific band signals in each lane alongside housekeeping protein (vinculin) were obtained, and the background signal was subtracted. The following antibodies were used for the analysis: phospho-ACC (Ser79) (Cell Signaling Technology, 3661), ACC (Cell Signaling Technology, 3662), phospho-AMPKα (Thr172) (Cell Signaling Technology, 2535), AMPKα (Cell Signaling Technology, 2532), vinculin (Cell Signaling Technology, 13901), Peroxidase AffiniPure Donkey Anti-Rabbit IgG (H + L) (Jackson ImmunoResearch, 711-035-152).

### Blue native-PAGE

Mitochondria (70 μg) were solubilized in lysis buffer (20 mM Tris-Cl, 10% (v/v) glycerol, 100 mM NaCl, 20 mM imidazole, 1 mM CaCl_2_, pH 7.4) containing 1% (w/v) digitonin (Biosynth International) and supplemented with protease inhibitors (1 mM PMSF, 2 mM pepstatin A, 10 mM leupeptin). Protein extracts were collected as supernatant following centrifugation (21,000 *g* for 30 min, 4 °C) and mixed with 10× blue native-PAGE sample buffer (5% (w/v) Coomassie brilliant blue G-250 (Serva), 0.5 M 6-aminocaproic acid and 10 mM bis-tris/HCl (pH 7.0)). The extracts were resolved by 4% to 16% house-made 1D blue native-PAGE gel. The two-dimensional blue native-SDS–PAGE analysis was performed as previously described^87^.

### NAC treatment

Group-housed mice were given free access to water supplemented with 10 mg ml^−1^ NAC for 8 weeks. The water bottles were changed twice per week.

### Glucose uptake ex vivo in incubated skeletal muscles

EDL and SOL muscles were dissected from MUCKO mice and control littermates, and glucose uptake were measured ex vivo as previously described with small modifications^88^. Muscles were applied to a tension of 5 mN per muscle and pre-incubated in a glucose-based Krebs–Ringer buffer containing 117 mM NaCl, 4.7 mM KCl, 2.5 mM CaCl_2_, 1.2 mM KH_2_PO_4_, 1.2 mM MgSO_4_, 24.6 mM NaHCO_3_ supplemented with 0.1% bovine serum albumin, 8 mM mannitol and 5 mM glucose, with 20 mM NAC or 10 mM NaCl (control). After 1 h, the buffer was changed to a pyruvate-based Krebs–Ringer buffer: 117 mM NaCl, 4.7 mM KCl, 2.5 mM CaCl_2_, 1.2 mM KH_2_PO_4_, 1.2 mM MgSO_4_, 24.6 mM NaHCO_3_ supplemented with 0.1% bovine serum albumin, 8 mM mannitol and 2 mM pyruvate, with 20 mM NAC or 10 mM NaCl, and with 10 mU ml^−1^ insulin (Actrapid, Novo Nordisk) or vehicle as indicated. Glucose uptake was assessed using 1 mM 2-DG together with [^3^H]-2-deoxy-ᴅ-glucose (0.0185 MBq ml^−1^, PerkinElmer, NET549A005MC) as a tracer, and [^14^C]-mannitol (0.0167 MBq ml^−1^, PerkinElmer, NEC314250UC) to determine extracellular space, as described in the original protocol.

### Lipid peroxidation

The level of 4-HNE in muscle tissue lysates was measured with the 4-HNE Assay Kit (Abcam, catalog number ab238538), according to the manufacturer’s instructions.

### Statistics and reproducibility

Unless otherwise stated, data are presented as mean ± s.e.m. No statistical methods were used to predetermine sample sizes; however, sample sizes are comparable to those reported in previous publications in the field^23^. No method of randomization was used to assign animals to groups at the onset of the study. Data collection and analysis were not performed blind to experimental conditions, and blinding was not applied in any of the depicted studies. Data distribution was assumed to be normal but was not formally tested. No data were excluded from analyses or figure depictions. At least three biologically independent experiments were performed in each case unless otherwise stated in the respective figure legend.

Unless otherwise noted, statistical analyses were performed using GraphPad Prism 10. Differences between two groups were assessed with an unpaired, two-tailed Student’s *t*-test. Differences in gene expression between control and MUCKO animals were assessed with *t*-tests corrected for multiple comparisons using the Bonferroni method. Data with two factors and repeated measurements were analyzed by two-way repeated-measures ANOVA with Bonferroni’s post hoc correction. Data with two independent factors were assessed by two-way ANOVA with Bonferroni’s post hoc correction. For RNA-seq and proteomics data, differential features were considered significant at an *P*_Adj_ < 0.05 (Fisher’s exact test with FDR correction). For all other analyses, *P* < 0.05 was considered statistically significant.

### Reporting summary

Further information on research design is available in the Nature Portfolio Reporting Summary linked to this article.

## Supplementary information

Reporting Summary

Supplementary Data 1 Lipidomics (normalized lipid species) in SOL and EDL from control and MUCKO mice.

Supplementary Data 2 Proteomics (normalized abundance) in SOL and EDL from MUCKO versus control mice.

Supplementary Data 3 Metabolomics (protein corrected) in SOL and EDL from control and MUCKO mice.

Supplementary Table 1 Oligonucleotide sequences.

## Source data

Source Data Figs. 1–5 and Extended Data Figs. 1–7 Statistical source data.

Source Data Figs. 3 and 5 and Extended Data Figs. 2, 4 and 6 Uncropped western blots.

## Data Availability

Data that support the findings of this study are available in the Article and Supplementary Information. The RNA-sequencing datasets generated in this study are deposited at ArrayExpress under accession numbers E-MTAB-17333 (MUCKO/control, SOL and EDL), E-MTAB-17320 (aged and young mice, SOL and EDL) and E-MTAB-17526 (C2C12 *Crls1* KD with ERRγ inhibition). Additional lipidomic, proteomic and metabolomic data from the MUCKO versus control comparison in SOL and EDL are available in Supplementary Data 1–3. Source data are provided with this paper.
