# Supplementary material for: Mitochondrial membrane lipid cardiolipin controls fiber-type adaptations in aging muscle via estrogen-related receptor γ
Source: Nat Aging. 2026 Sep 29;6(10):2092–109. doi: 10.1038/s43587-026-01227-7 (PMC13661251; doi:10.1038/s43587-026-01227-7)
Supplement: Supplementary file 1 [file 43587_2026_1227_MOESM1_ESM.pdf]

nature portfolio

Corresponding author(s): Zachary Gerhart-Hines, Fabian Finger

Last updated by author(s): Aug 18, 2026

# Reporting Summary

Nature Portfolio wishes to improve the reproducibility of the work that we publish. This form provides structure for consistency and transparency in reporting. For further information on Nature Portfolio policies, see our Editorial Policies and the Editorial Policy Checklist.

## Statistics

For all statistical analyses, confirm that the following items are present in the figure legend, table legend, main text, or Methods section.

| n/a | Confirmed | |
|---|---|---|
| ☐ | ☒ | The exact sample size ($n$) for each experimental group/condition, given as a discrete number and unit of measurement |
| ☐ | ☒ | A statement on whether measurements were taken from distinct samples or whether the same sample was measured repeatedly |
| ☐ | ☒ | The statistical test(s) used AND whether they are one- or two-sided<br>*Only common tests should be described solely by name; describe more complex techniques in the Methods section.* |
| ☒ | ☐ | A description of all covariates tested |
| ☐ | ☒ | A description of any assumptions or corrections, such as tests of normality and adjustment for multiple comparisons |
| ☐ | ☒ | A full description of the statistical parameters including central tendency (e.g. means) or other basic estimates (e.g. regression coefficient) AND variation (e.g. standard deviation) or associated estimates of uncertainty (e.g. confidence intervals) |
| ☐ | ☒ | For null hypothesis testing, the test statistic (e.g. $F$, $t$, $r$) with confidence intervals, effect sizes, degrees of freedom and $P$ value noted<br>*Give P values as exact values whenever suitable.* |
| ☒ | ☐ | For Bayesian analysis, information on the choice of priors and Markov chain Monte Carlo settings |
| ☒ | ☐ | For hierarchical and complex designs, identification of the appropriate level for tests and full reporting of outcomes |
| ☒ | ☐ | Estimates of effect sizes (e.g. Cohen's $d$, Pearson's $r$), indicating how they were calculated |

*Our web collection on statistics for biologists contains articles on many of the points above.*

## Software and code

Policy information about availability of computer code

| | |
|---|---|
| Data collection | Fiji ImageJ, Odyssey XF imaging system (LICORbio), no custom code was used |
| Data analysis | GraphPad Prism 8 & 10 (GraphPad Software), ImageStudio Lite Ver 5.2, no custom code was used |

For manuscripts utilizing custom algorithms or software that are central to the research but not yet described in published literature, software must be made available to editors and reviewers. We strongly encourage code deposition in a community repository (e.g. GitHub). See the Nature Portfolio guidelines for submitting code & software for further information.

## Data

Policy information about availability of data

All manuscripts must include a data availability statement. This statement should provide the following information, where applicable:

- Accession codes, unique identifiers, or web links for publicly available datasets
- A description of any restrictions on data availability
- For clinical datasets or third party data, please ensure that the statement adheres to our policy

RNA-seq datasets (MUCKO soleus/EDL: E-MTAB-17333; young vs. aged mouse muscle: E-MTAB-17320; Crls1-KD C2C12 ERRγ inhibition: E-MTAB-17526) are deposited in ArrayExpress.

# Research involving human participants, their data, or biological material

Policy information about studies with human participants or human data. See also policy information about sex, gender (identity/presentation), and sexual orientation and race, ethnicity and racism.

| | |
|---|---|
| Reporting on sex and gender | Biospecimens were obtained from a previously published human study (PMID: 34273538) that included both men and women; detailed sex distribution is reported in that publication. |
| Reporting on race, ethnicity, or other socially relevant groupings | Race/ethnicity and other demographic characteristics were collected and are reported in the original study (PMID: 34273538); the present analyses used de-identified samples without additional stratification by race/ethnicity. |
| Population characteristics | See above |
| Recruitment | No new participants were recruited. Samples were obtained from an existing, previously published cohort (PMID: 34273538); recruitment procedures and consent are described in that publication. |
| Ethics oversight | Collection of human samples was approved by the local ethics committee at the University of Copenhagen (H-3-2012-024), as reported in PMID: 34273538. The present work is a secondary analysis of those stored samples under the same approval. |

Note that full information on the approval of the study protocol must also be provided in the manuscript.

# Field-specific reporting

Please select the one below that is the best fit for your research. If you are not sure, read the appropriate sections before making your selection.

☒ Life sciences ☐ Behavioural & social sciences ☐ Ecological, evolutionary & environmental sciences

For a reference copy of the document with all sections, see nature.com/documents/nr-reporting-summary-flat.pdf

# Life sciences study design

All studies must disclose on these points even when the disclosure is negative.

| | |
|---|---|
| Sample size | Sample sizes were determined on the basis of estimates from preliminary experiments. |
| Data exclusions | No data were excluded from analysis or depictions within the manuscript. |
| Replication | At least three biologically independent experiments were performed in each case, unless otherwise stated in the respective figure legend. |
| Randomization | Randomization most directly applies to the animal studies in the manuscript. Experimental cohorts were assigned at random at the onset of the study. |
| Blinding | Blinding was not performed in the depicted studies. |

# Reporting for specific materials, systems and methods

We require information from authors about some types of materials, experimental systems and methods used in many studies. Here, indicate whether each material, system or method listed is relevant to your study. If you are not sure if a list item applies to your research, read the appropriate section before selecting a response.

## Materials & experimental systems

| n/a | Involved in the study | |
|---|---|---|
| ☐ | ☒ | Antibodies |
| ☐ | ☒ | Eukaryotic cell lines |
| ☒ | ☐ | Palaeontology and archaeology |
| ☐ | ☒ | Animals and other organisms |
| ☒ | ☐ | Clinical data |
| ☒ | ☐ | Dual use research of concern |
| ☒ | ☐ | Plants |

## Methods

| n/a | Involved in the study | |
|---|---|---|
| ☒ | ☐ | ChIP-seq |
| ☒ | ☐ | Flow cytometry |
| ☒ | ☐ | MRI-based neuroimaging |

## Antibodies

| | |
|---|---|
| Antibodies used | MHC type I (DSHB, A4.840), MHC type IIa (DSHB, SC-71), MHC type IIx (DSHB, 6H1), MHC type IIb (DSHB, BF-F3), MyoG (DSHB, F5D), |

| | |
|---|---|
| Antibodies used | GAPDH (Cell Signaling Technology, 14C10), Phospho-ACC(Ser79) (Cell Signaling Technology, #3661), ACC (Cell Signaling Technology, #3662), Phospho-AMPKa(Thr172) (Cell Signaling Technology, #2535), AMPKa (Cell Signaling Technology, #2532), Glut4 (AbCam, #ab654), Phospho-STBD1(Ser175) (Custom-made, YenZym,#YZ7509), STBD1 (ProteinTech, #11842-1-AP), Vinculin (Cell Signaling Technology, #13901), Peroxidase AffiniPure™ Donkey Anti-Rabbit IgG (H+L) (Jackson ImmunoResearch, #711-035-152). |
| Validation | The listed primary antibodies have been validated by the stated commercial provider. |

## Eukaryotic cell lines

Policy information about cell lines and Sex and Gender in Research

| | |
|---|---|
| Cell line source(s) | C2C12 murine muscle myoblasts |
| Authentication | None of the cell lines used were authenticated. |
| Mycoplasma contamination | All cell lines used were tested negative for mycoplasma contamination. |
| Commonly misidentified lines (See ICLAC register) | No commonly misidentified cell lines were used in this study. |

## Animals and other research organisms

Policy information about studies involving animals; ARRIVE guidelines recommended for reporting animal research, and Sex and Gender in Research

| | |
|---|---|
| Laboratory animals | species: mus musculus; strains: Crls1f/f; Crls1f/f, HSA-cre; Crls1f/f, HSA-MCM |
| Wild animals | The study did not involve wild animals. |
| Reporting on sex | The phenotypes described apply to both sexes. |
| Field-collected samples | The study did not involve samples collected from the field. |
| Ethics oversight | All animal experiments were conducted in accordance with the Danish Animal Research Council under licence no. 2018-15-0201-01441 or were approved by the Ethics Committee for Animal Welfare of the State Office of Environment, Health, and Consumer Protection of the Federal State of Brandenburg, Germany. |

Note that full information on the approval of the study protocol must also be provided in the manuscript.

## Plants

| | |
|---|---|
| Seed stocks | No seed stock or other plant materials were used in this study. |
| Novel plant genotypes | No novel plant genotypes were produced in this study. |
| Authentication | No authentication was needed in this study. |
