# Supplementary material for: Mitochondrial membrane lipid cardiolipin controls fiber-type adaptations in aging muscle via estrogen-related receptor γ
Source: Nat Aging. 2026 Sep 29;6(10):2092–109. doi: 10.1038/s43587-026-01227-7 (PMC13661251; doi:10.1038/s43587-026-01227-7)

Extended Data Fig. 2E

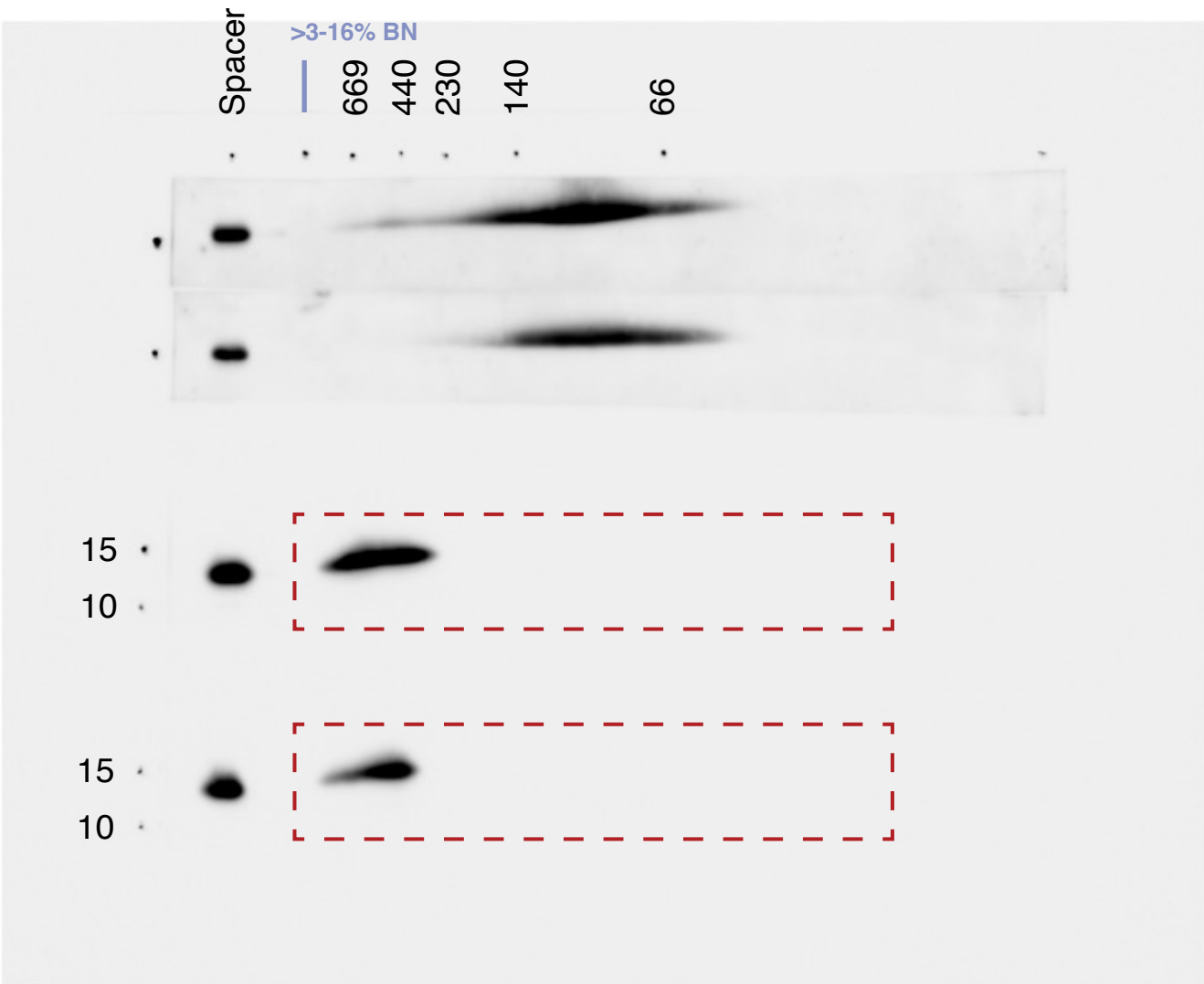

CI: NDUFB6

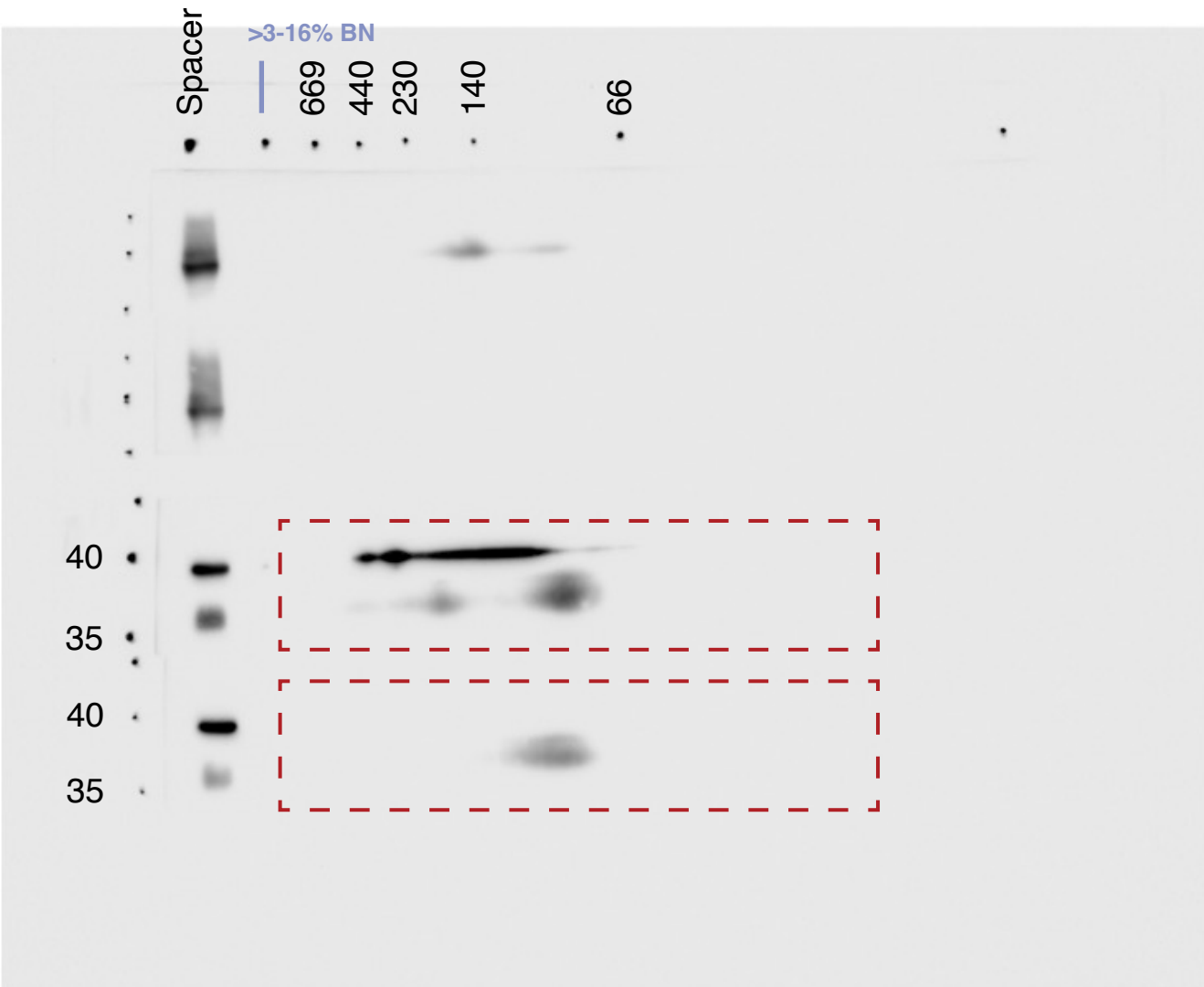

CIII: UQCRC2
CIV: MT-CO1

Extended Data Fig. 6A+D

p-Thr172 AMPKα

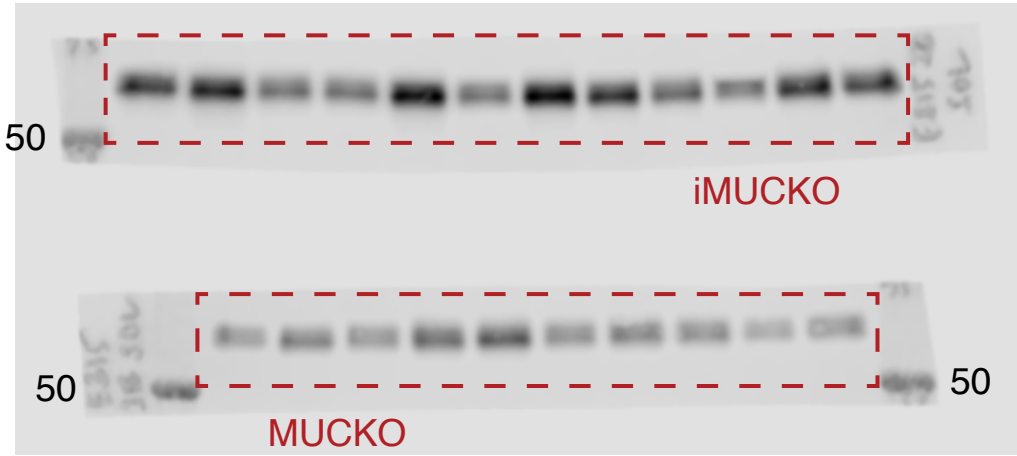

AMPKα

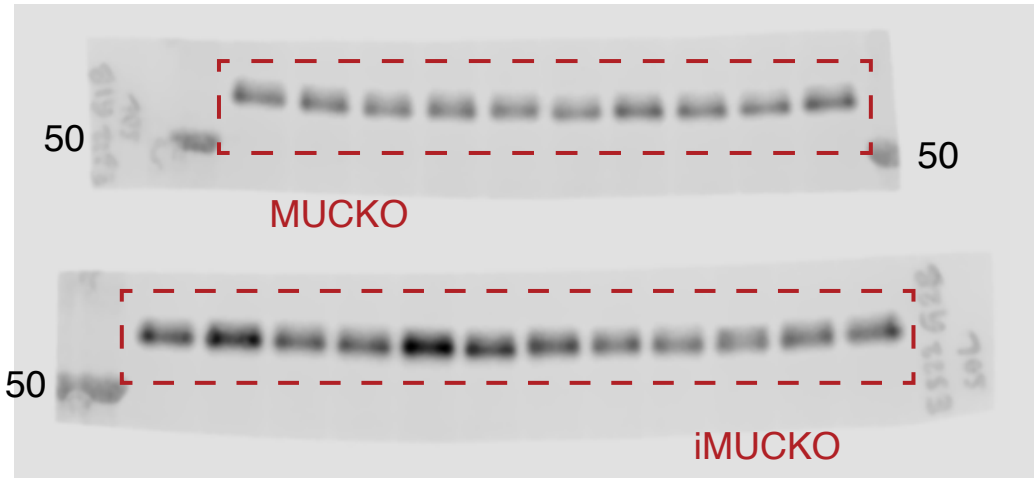

p-Ser212 ACC2

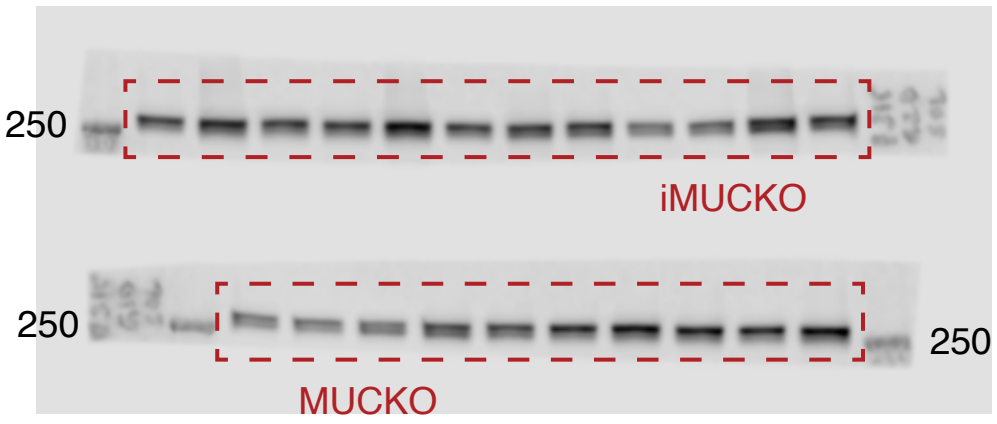

ACC2

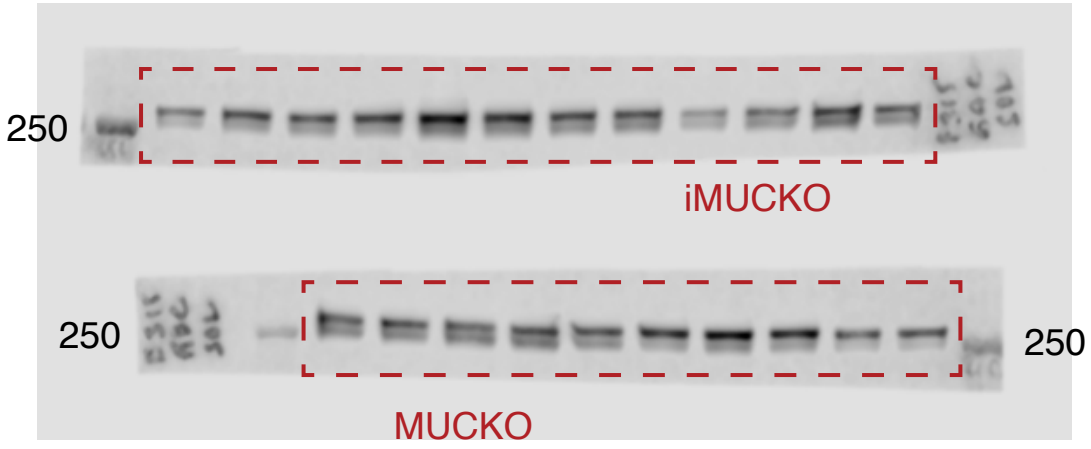

Vinculin

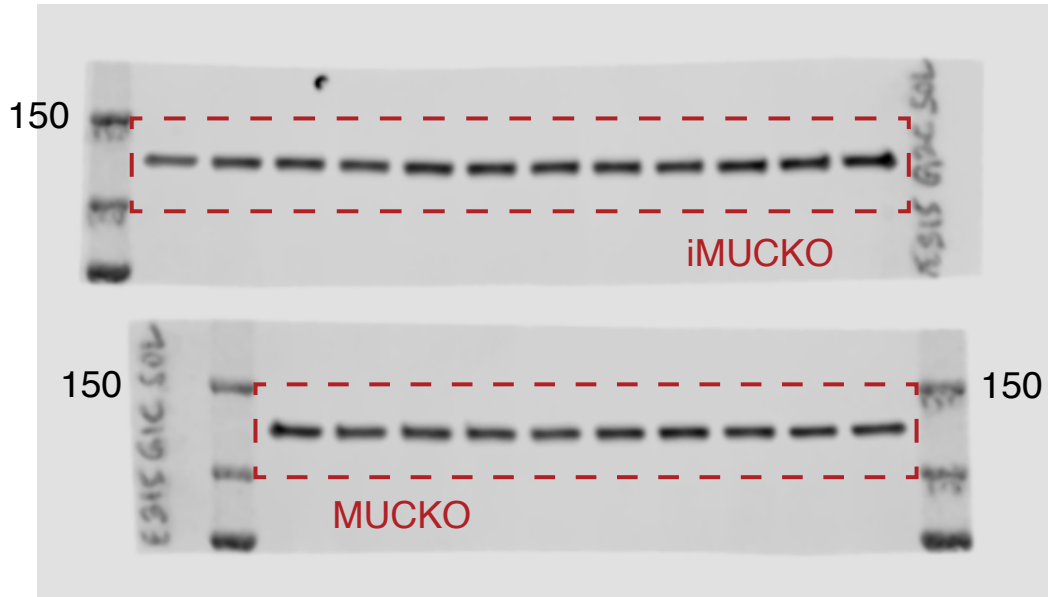

MUCKO western blots horizontally flipped in the main figure to match sample order; KO samples were loaded before controls.

Extended Data Fig. 6B+E

p-Thr172 AMPKα

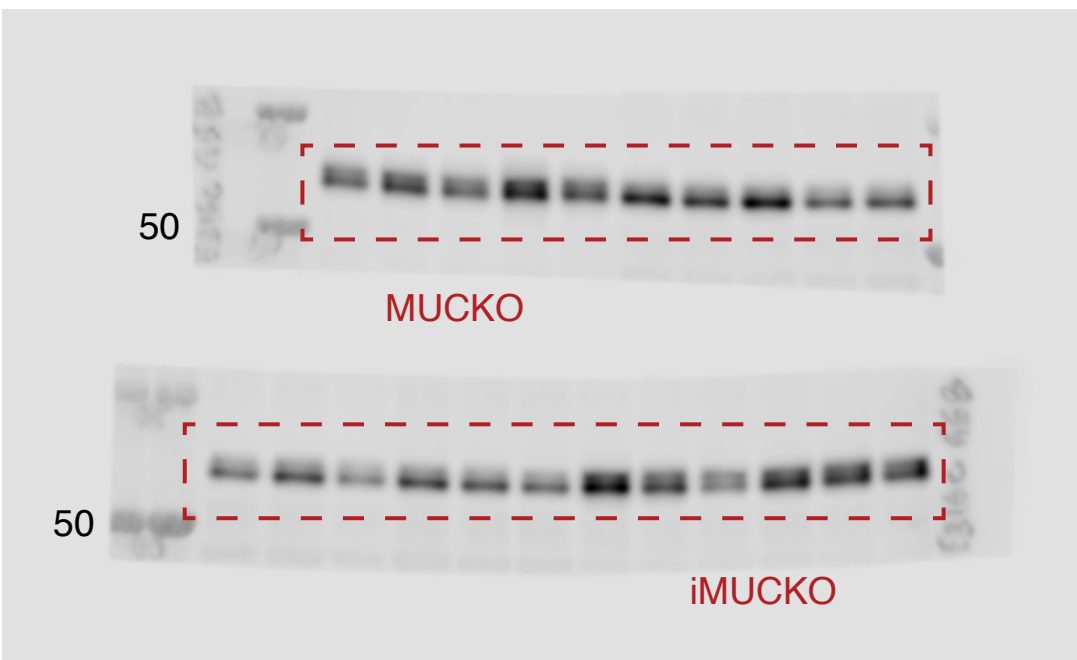

AMPKα

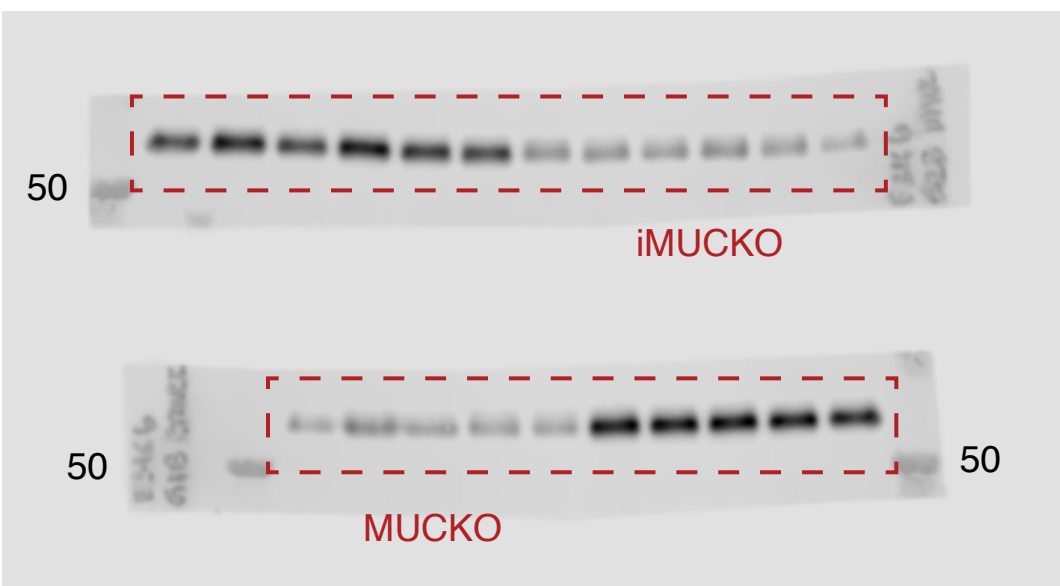

p-Ser212 ACC2

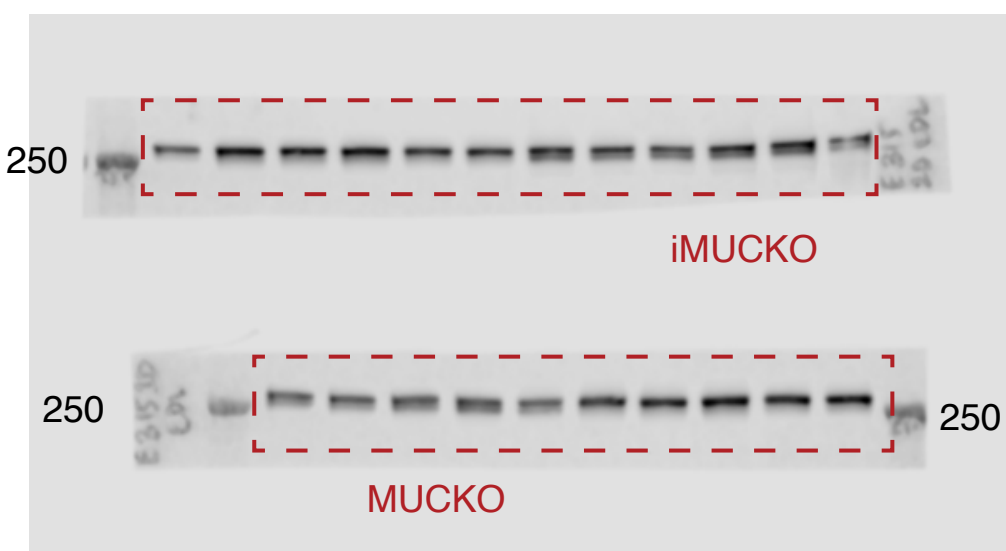

ACC2

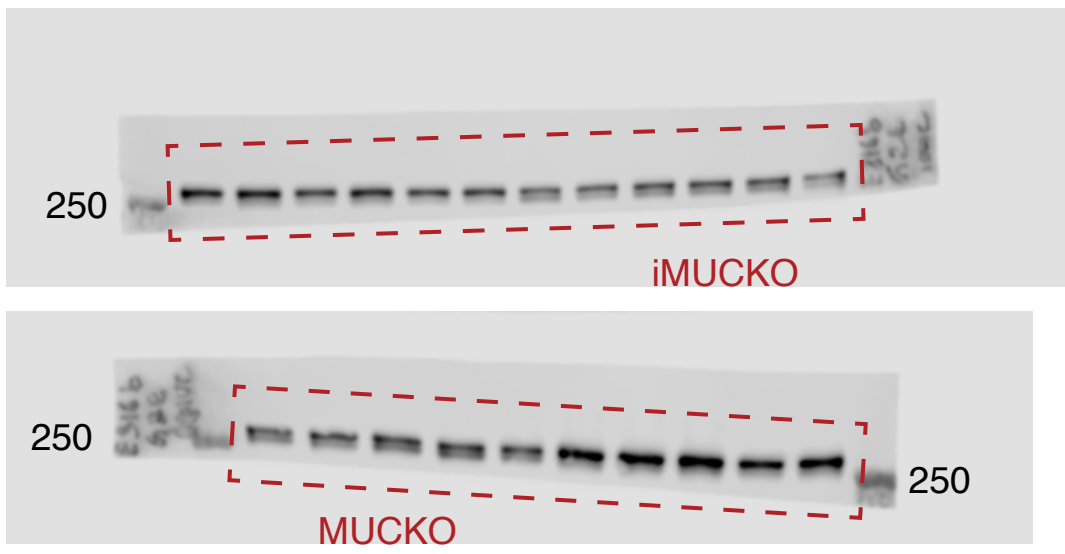

Vinculin

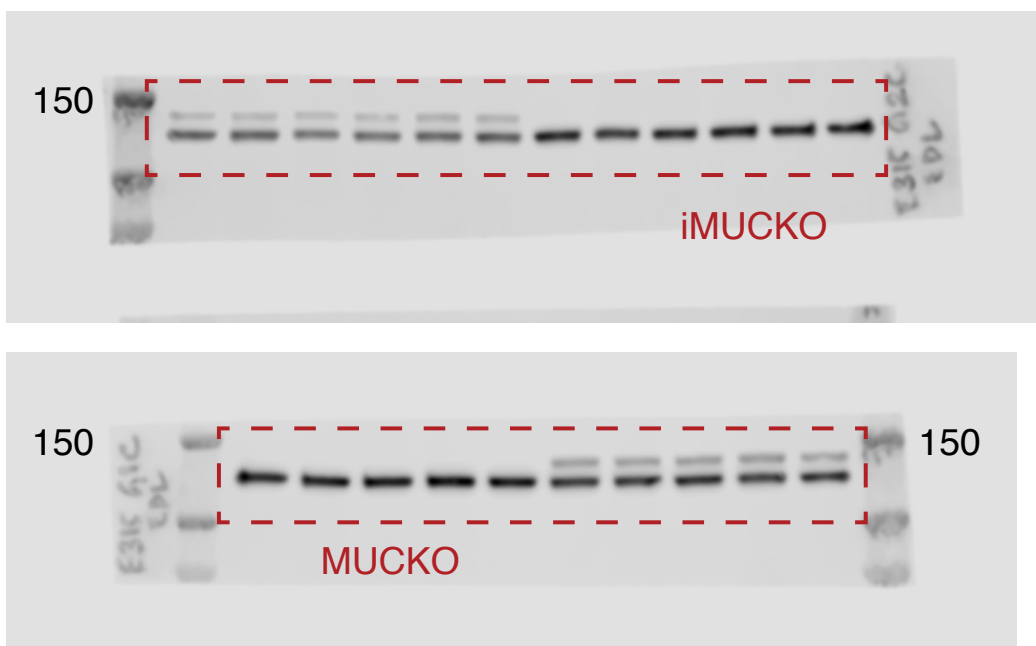

MUCKO western blots horizontally flipped in the main figure to match sample order; KO samples were loaded before controls.

Figure 3D

MHC I

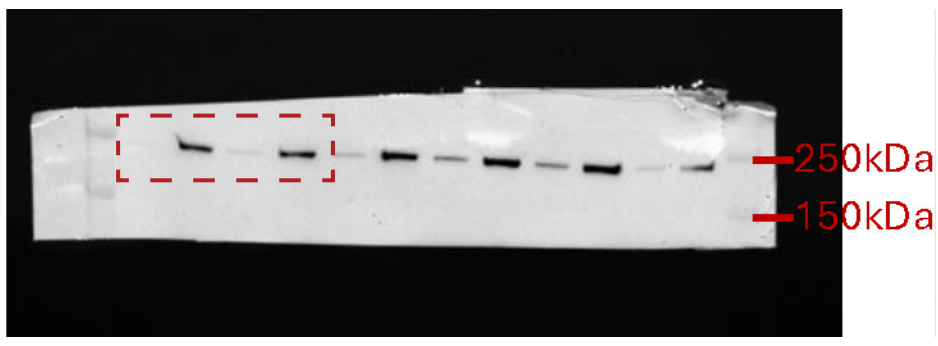

MHC IIa

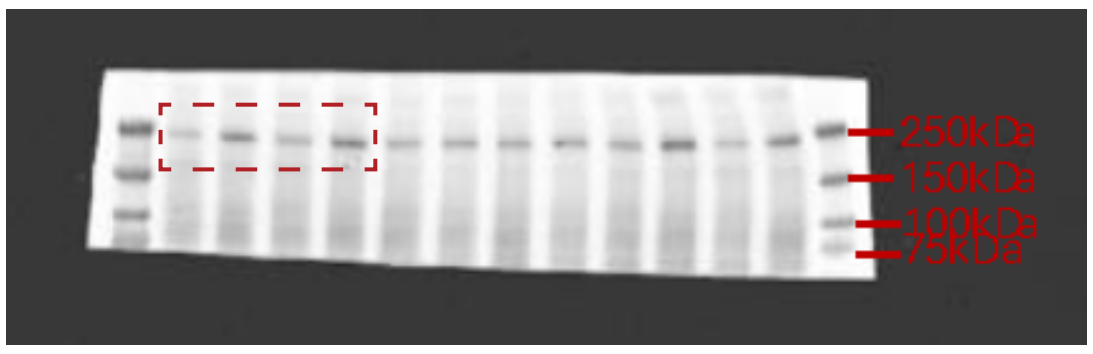

MHC IIx

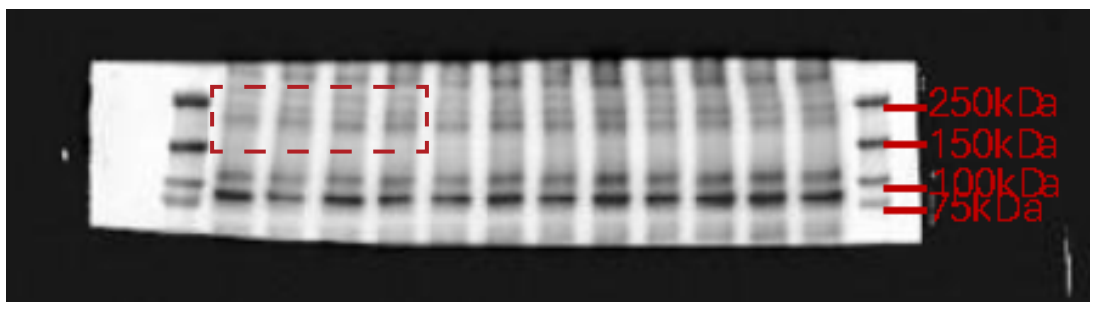

MHC IIb

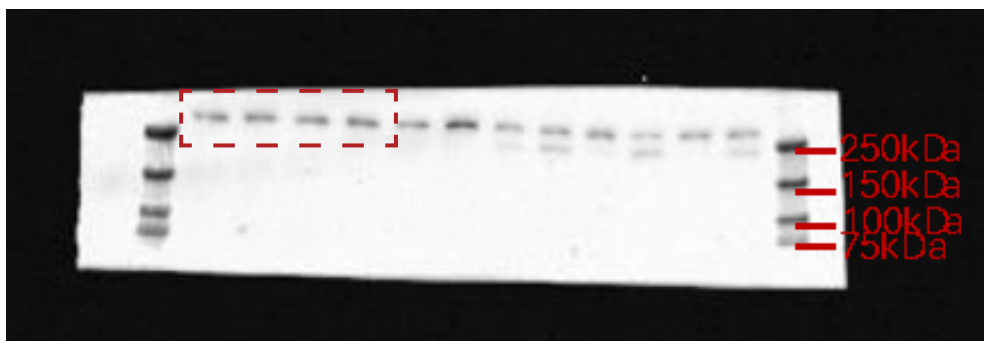

GAPDH

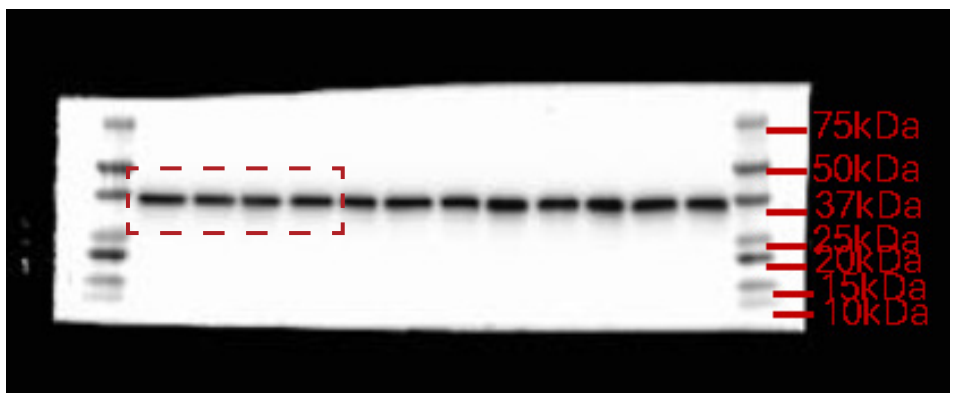

Figure 3E

MHC I

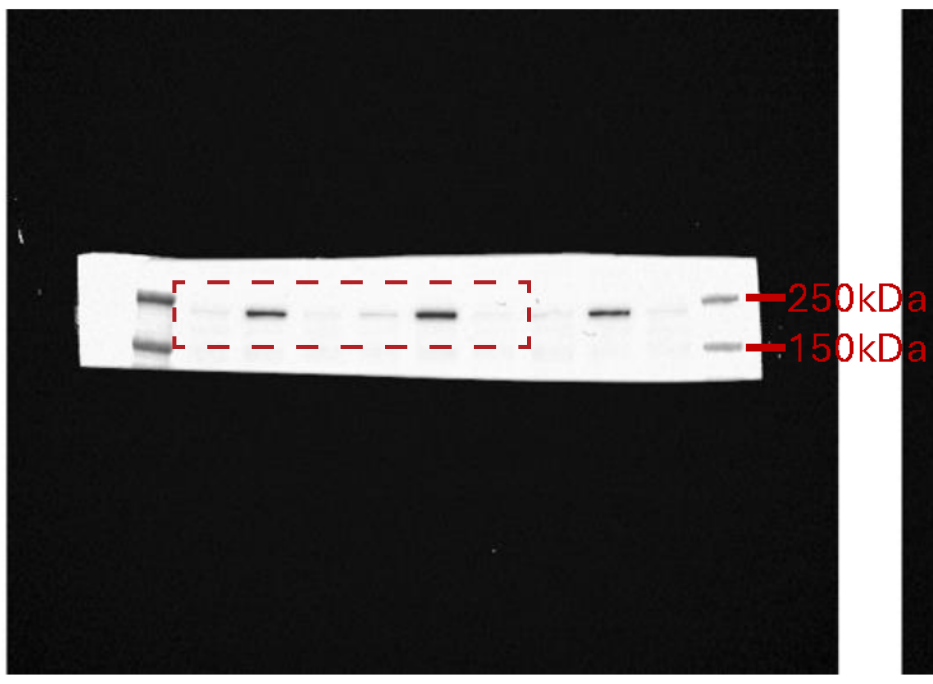

MHC IIb

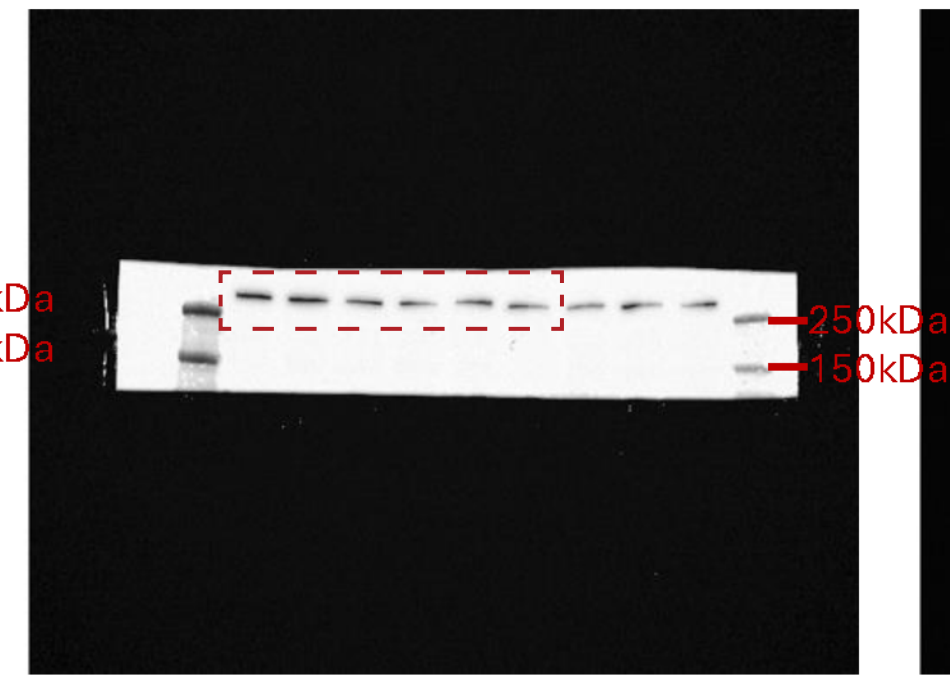

GAPDH

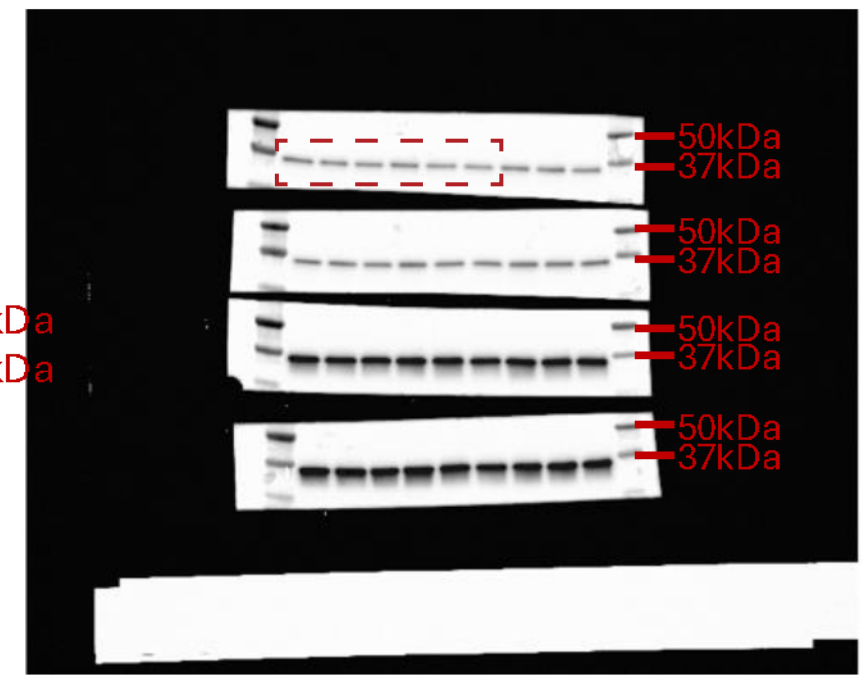

Figure 3F

MHC I

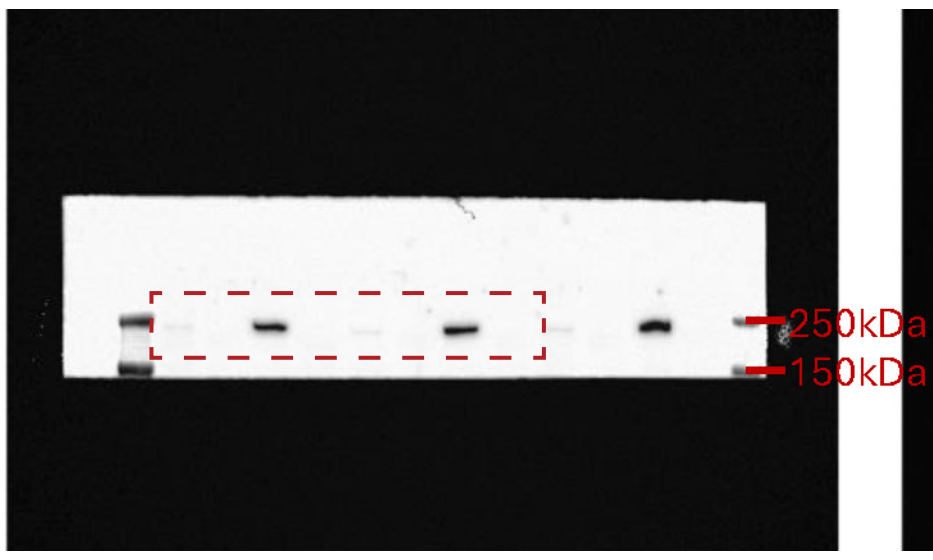

MHC IIb

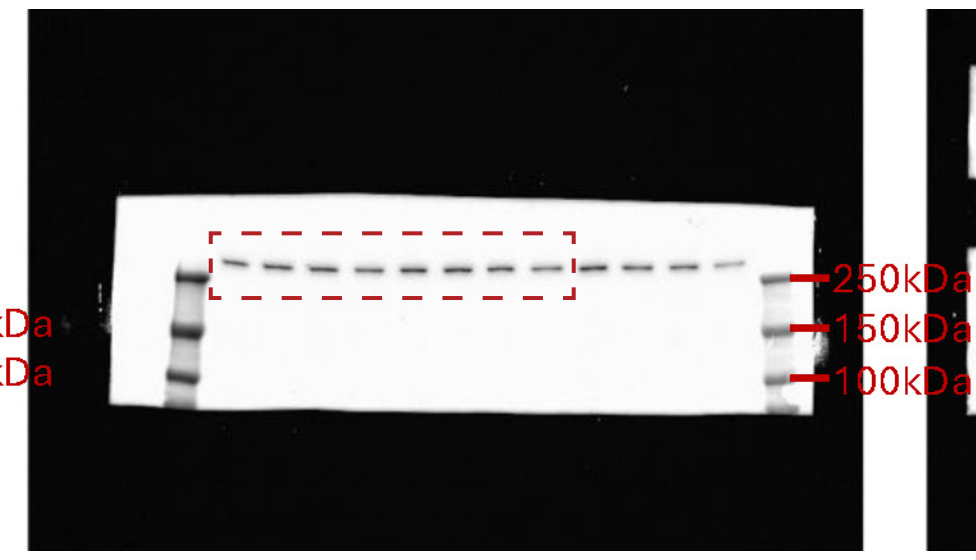

GAPDH

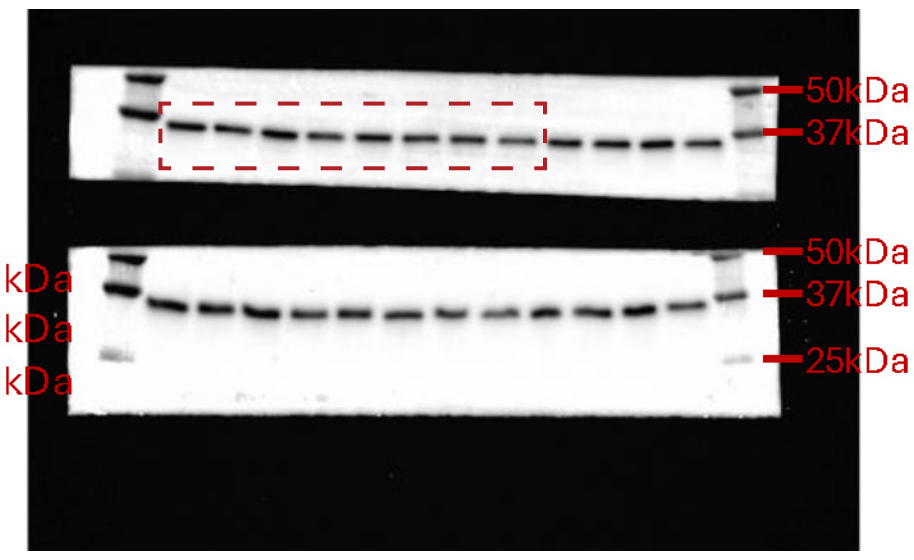

Figure 5K

MHC I

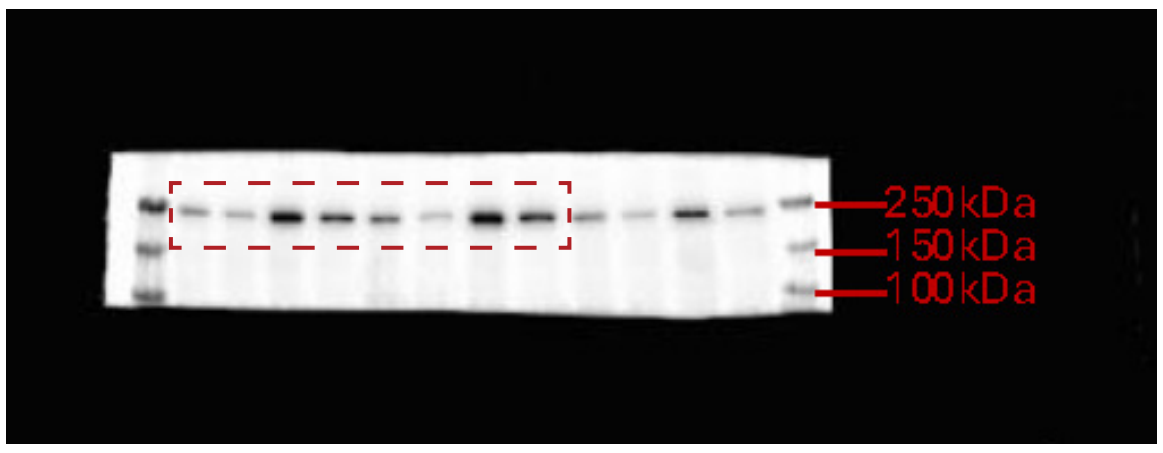

MHC IIb

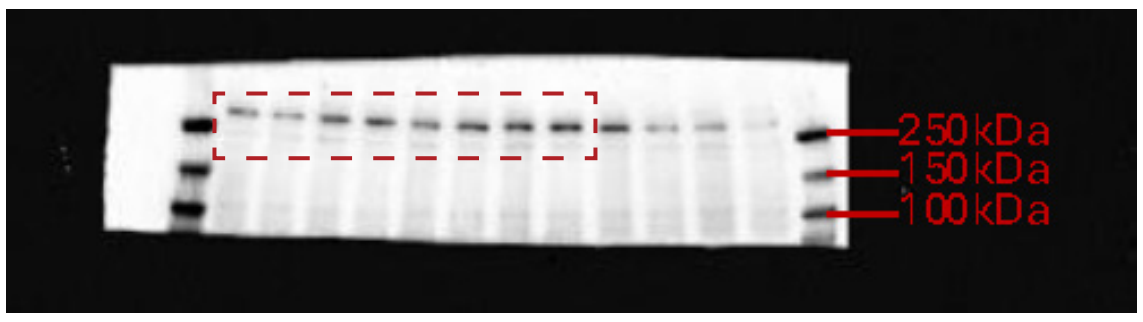

GAPDH

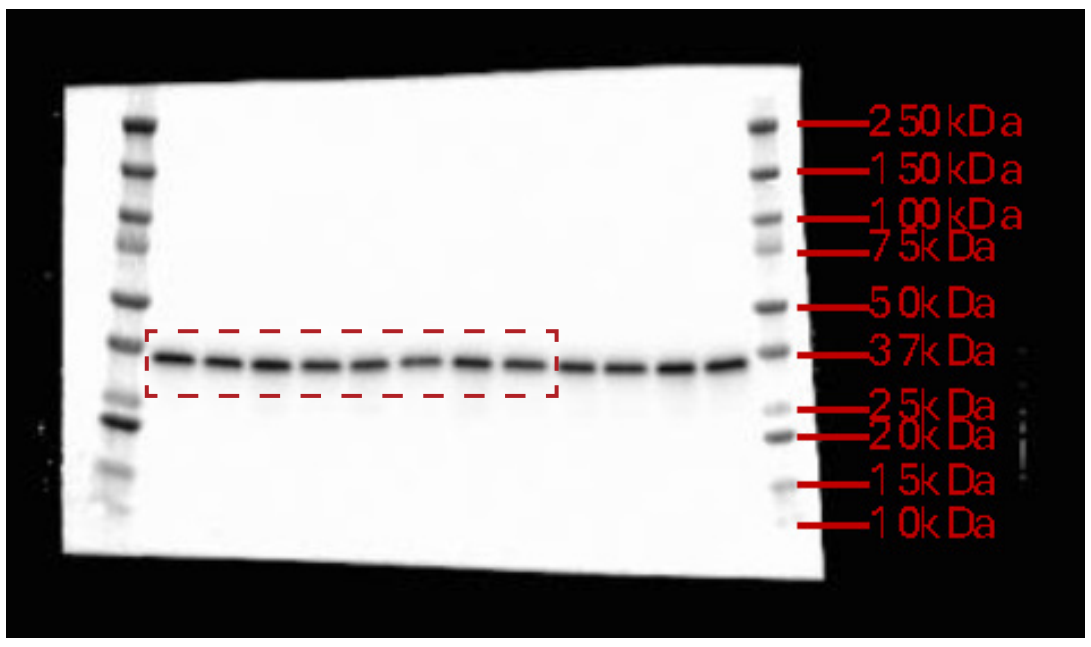

Extended Data Fig. 4C

MHC IIa

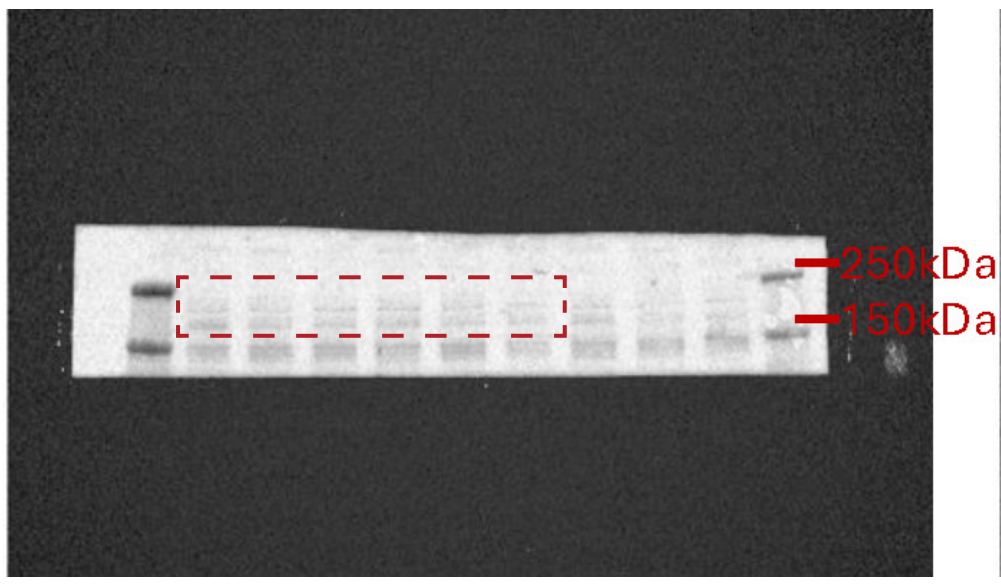

MHC IIx

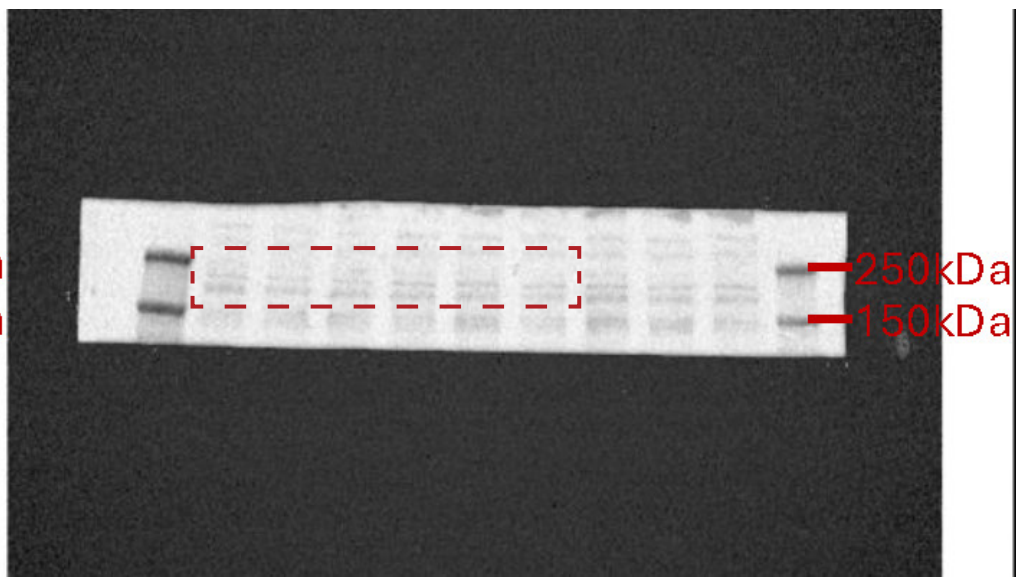

GAPDH

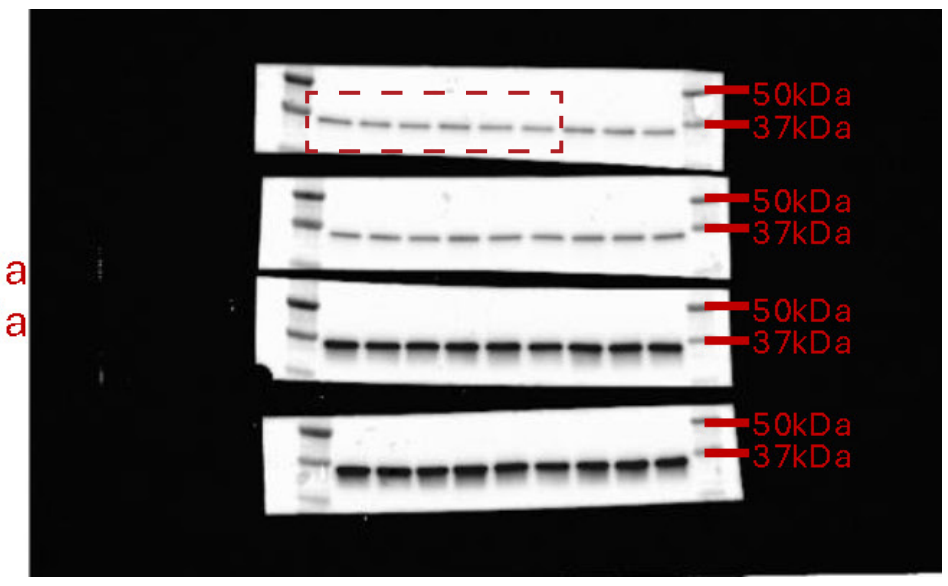

Extended Data Fig. 4D

MHC IIa

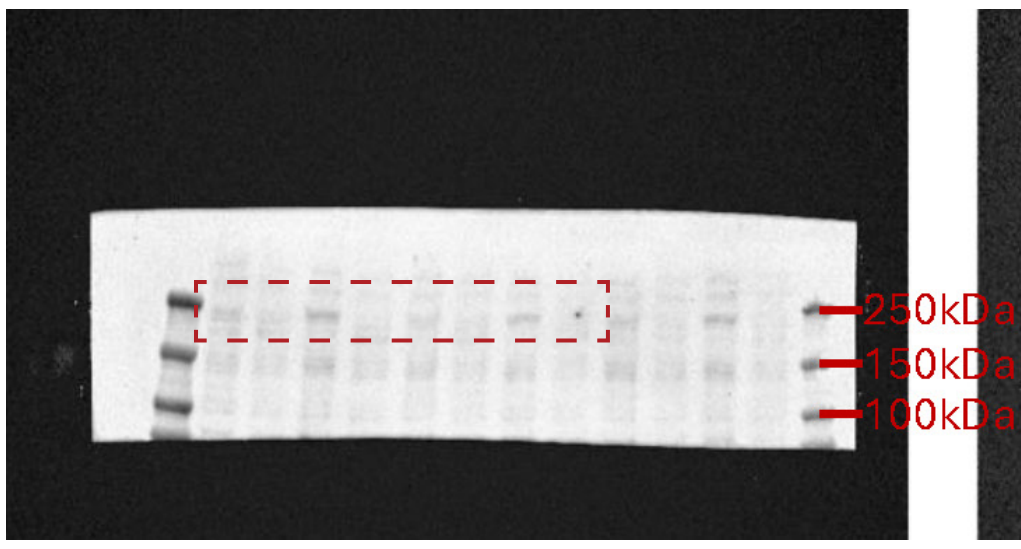

MHC IIx

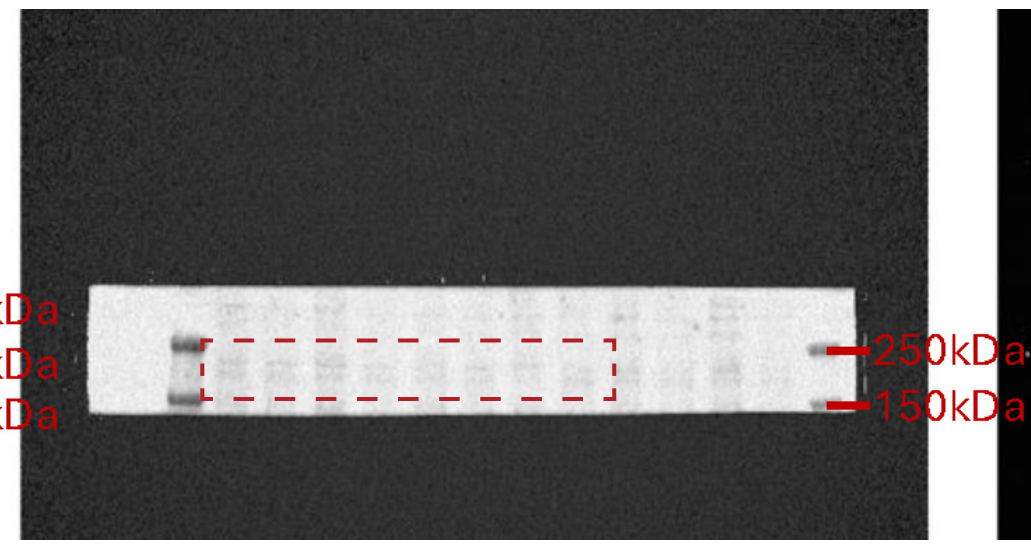

GAPDH

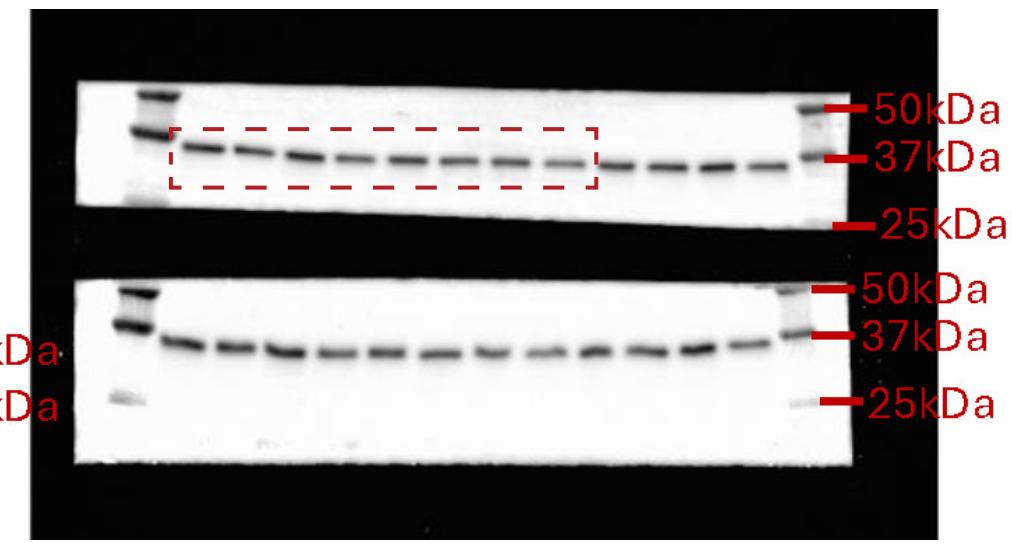

Extended Data Fig. 4D

Myogenin

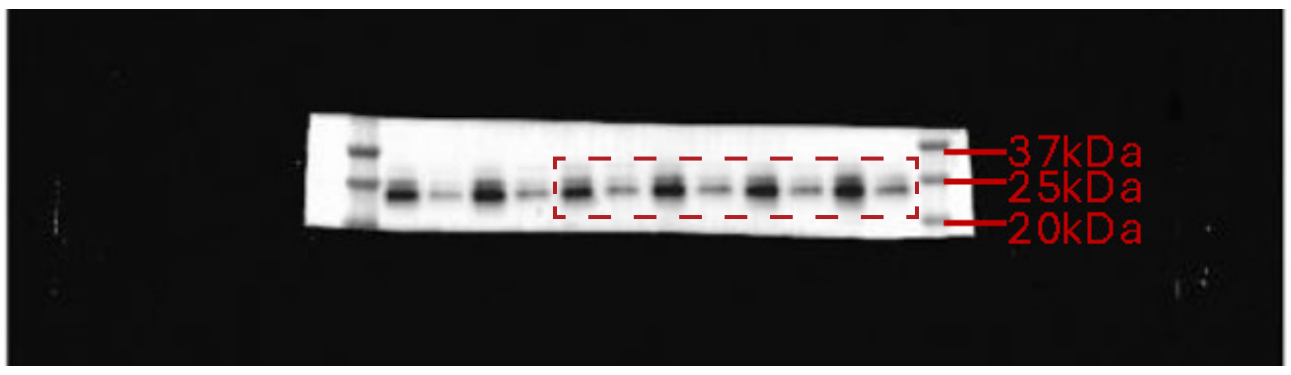

GAPDH

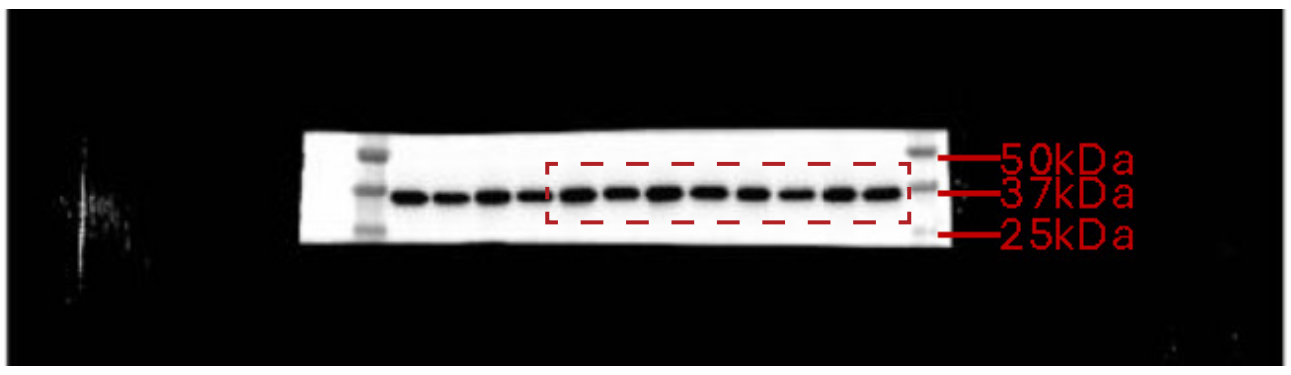

Extended Data Fig. 4F

MHC I

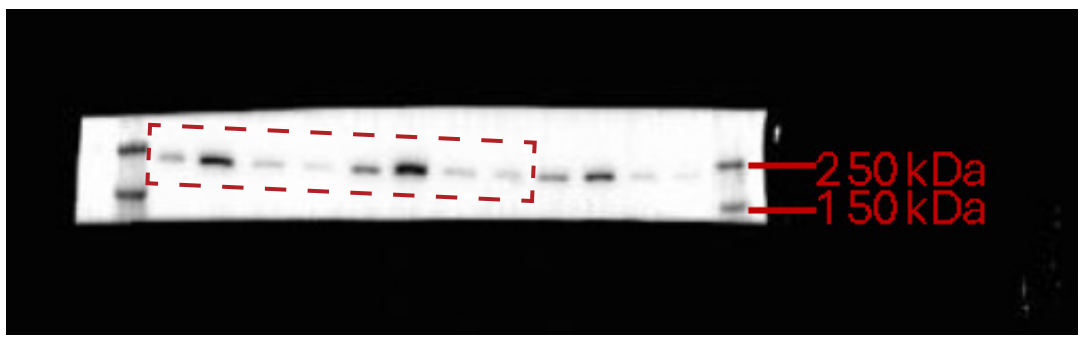

MHC IIa

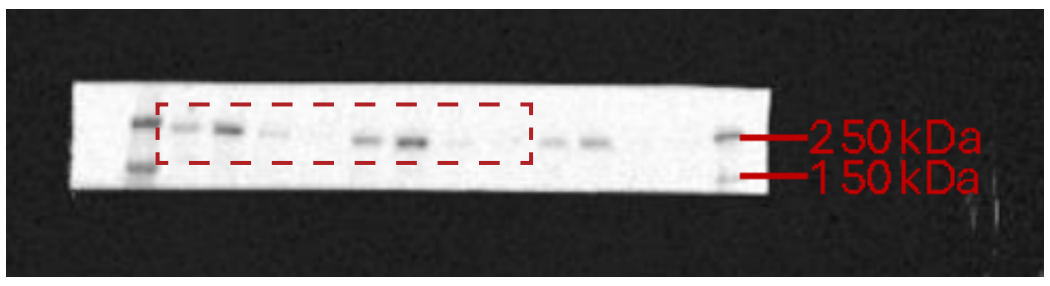

MHC IIx

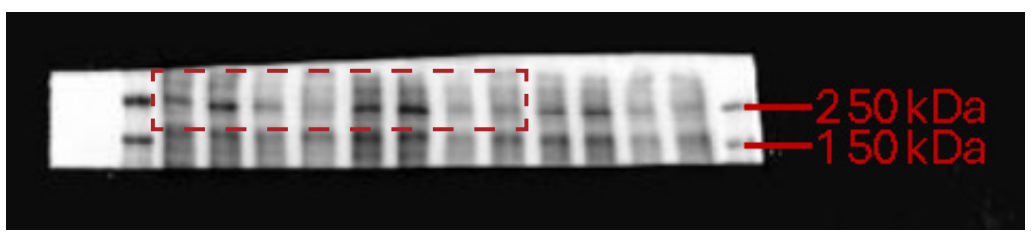

MHC IIb

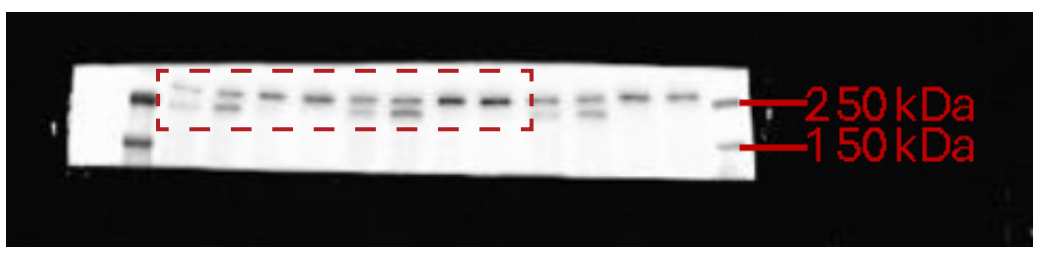

GAPDH

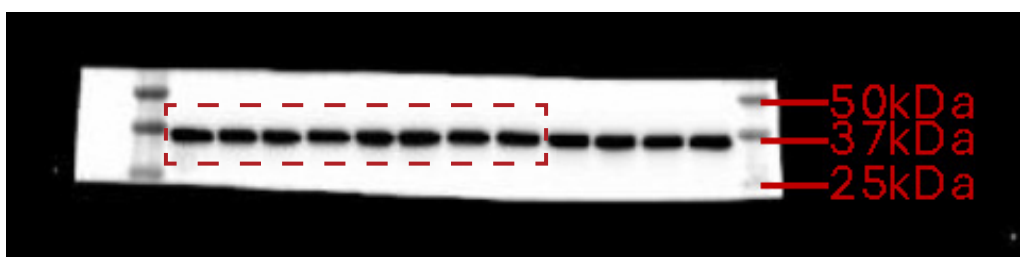

Supplement: Supplementary file 2 [file 43587_2026_1227_MOESM7_ESM.pdf]
